# Supplementary material for: Diastereoselective Synthesis of the Borylated d-Galactose Monosaccharide 3-Boronic-3-Deoxy-d-Galactose and Biological Evaluation in Glycosidase Inhibition and in Cancer for Boron Neutron Capture Therapy (BNCT)
Source: Molecules. 2023 May 24;28(11):4321. doi: 10.3390/molecules28114321 (PMC10254170; doi:10.3390/molecules28114321)
Supplement: Supplementary file 1 [file molecules-28-04321-s001.zip › molecules-2379987-supplementary.pdf]

Supplementary Information

Diastereoselective Synthesis of the Borylated D-Galactose Monosaccharide 3-Boronic-3-Deoxy-D-Galactose and Biological Evaluation in Glycosidase Inhibition and in Cancer for Boron Neutron Capture Therapy (BNCT)

Michela I. Simone [1,2]

[1] Discipline of Chemistry, University of Newcastle, Callaghan, NSW 2308, Australia; michela_simone@yahoo.co.uk or michela.simone@csiro.au
[2] Newcastle CSIRO Energy Centre, 10 Murray Dwyer Circuit, Mayfield West, NSW2304, Australia

Index

1. **Further Synthetic Details**
1.1. **About the chemical stability of alkene 3**.
1.2. **Hydroboration/borane-trapping optimisation**
1.3. **Purification protocol optimisation (Table S2)**
2. **NMR Spectra**

1. Further Synthetic Details

1.1. About the chemical stability of alkene 3.

It was noticed alkene **3** reacted overtime, when stored at room temperature. Storage in freezing temperature was required and at -25°C the alkene survived unchanged for at least 12 months. Alkene **3** was also found to quickly react in the presence of chloroform. This was noticed during overnight NMR data acquisition. Hence, all data collections measured in solution were carried out in acetone or deuterated acetone, in which the alkene intermediate **3** displays stability over the data collection time.

1.2. Hydroboration/borane-trapping optimisation

The trapping of the borane with an organic nucleophile was a challenging transformation. However, it was possible to optimise this reaction and to develop a one-pot procedure with the hydroboration step (see video in Supplementary Information). The trapping was investigated with several organic nucleophiles.

Trapping with methanol. Methanol was initially used hoping that an acidic wash would have transformed *in situ* the boronic methyl ether into the corresponding boronic acid. It would have been advantageous to characterise the resulting doubly acetonide-protected boronic acid product and confirm structural integrity before global deprotection. It was however difficult to isolate pure product *via* this route. Methanol trapping was followed by coevaporation with methanol three times. Partitioning in EtOAc/HCl (1 M) afforded the boronic acid product in both layers. Trapping with methanol and purification on silica gel by flash column chromatography afforded – as expected - several products, inclusive of a diastereoisomerically pure C-3 saturated 3-deoxy-1,2:5,6-di-*O*-isopropylidene-α-D-*xylo*-hexose [δ_H ($CDCl_3$, 400 MHz): 5.80 (1H, d, $J_{H\text{-}1,H\text{-}2}$ 3.8 Hz, H-1), 4.73 (1H, ddd, $J_{H\text{-}2,H\text{-}3}$ 6.1 Hz, $J_{H\text{-}2,H\text{-}1}$ 3.8 Hz, $J_{H\text{-}2,H\text{-}3'}$ 1.3 Hz, H-2),

4.44 (1H, dt, $J_{H-5,H-4}$ 8.2 Hz, $J_{H-5,H-6}$ 6.8 Hz, $J_{H-5,H-6'}$ 6.8 Hz, H-5), 4.05 (1H, dd, $J_{H-6',H-6}$ 8.2 Hz, $J_{H-6',H-5}$ 6.6 Hz, H-6′), 3.61 (1H, dd, $J_{H-6,H-6'}$ 8.2 Hz, $J_{H-6,H-5}$ 6.9 Hz, H-6), 4.11 (1H, td, $J_{H-4,H-3'}$ 4.0 Hz, $J_{H-4,H-5}$ 8.4 Hz, $J_{H-4,H-3}$ 4.0 Hz, H-4), 2.21 (1H, ddd, $J_{H-3,H-3'}$ 14.4 Hz, $J_{H-3,H-4}$ 8.5 Hz, $J_{H-3,H-2}$ 6.2 Hz, H-3), 1.82 (1H, ddd, $J_{H-3',H-3}$ 14.2 Hz, $J_{H-3',H-4}$ 4.1 Hz, $J_{H-3',H-2}$ 1.1 Hz, H-3′), 1.57, 1.45, 1.37, 1.33 (12H, 4 x s, 4 x CH_3). δ_C ($CDCl_3$, 100 MHz): 112.9, 110.0 (2 x C_{quat} acetonides), 106.5 (C-1), 81.5 (C-4), 80.5 (C-2), 77.7 (C-5), 66.1 (C-6), 33.6 (C-3), 27.4, 26.8, 26.3, 25.3 (4 x CH_3)] (Supplementary Information, Figure S7) and whose characterisation data matched the literature[24-26] (R_f 0.80 in EtOAc as a white crystalline solid) and partially 5,6- and 1,2:5,6-deprotected boron-bearing monosaccharide derivatives. No signal was observed in the ^{11}B-NMR spectrum.

Other side-products obtained during the optimisation process included oxidised compounds from the borane intermediate, such as:
1,2:5,6-Di-*O*-isopropylidene-α-D-gulofuranose: δ_H ($CDCl_3$, 400 MHz): 5.78 (1H, d, $J_{H-1,H-2}$ 4.0 Hz, H-1), 4.66 (1H, dd, $J_{H-2,H-3}$ 6.3 Hz, $J_{H-2,H-1}$ 4.1 Hz, H-2), 4.47 (1H, dt, $J_{H-5,H-4}$ 8.4 Hz, $J_{H-5,H-6}$ 7.1 Hz, $J_{H-5,H-6'}$ 7.0 Hz, H-5), 4.25-4.19 (2H, m, H-3 and H-6′), 3.90 (1H, dd, $J_{H-4,H-5}$ 8.5 Hz, $J_{H-4,H-3}$ 5.7 Hz, H-4), 3.72 (1H, dd, $J_{H-6,H-6'}$ 8.5 Hz, $J_{H-6,H-5}$ 7.3 Hz, H-6), 2.65 (1H, d, $J_{OH-3,H-3}$ 6.5 Hz, OH-3), 1.63, 1.45, 1.43, 1.38 (12H, 4 x s, 4 x CH_3). δ_C ($CDCl_3$, 100 MHz): 115.2, 109.4 (2 x C_{quat} acetonides), 105.5 (C-1), 84.5 (C-4), 80.0 (C-2), 75.7 (C-5), 69.8 (C-3), 66.5 (C-6), 27.3 (2 x CH_3), 26.8, 25.4 (2 x CH_3)] (Supplementary Information, Figure S2) and whose characterisation data matched the literature (Table 1, **entry 4**).
1,2:5,6-Di-*O*-isopropylidene-α-D-galactofuranose: δ_H ($CDCl_3$, 400 MHz): 5.85 (1H, d, $J_{H-1,H-2}$ 3.8 Hz, H-1), 4.52 (1H, d, $J_{H-2,H-1}$ 3.8 Hz, H-2), 4.33 (1H, app-q, J 6.9 Hz, H-5), 4.08-4.05 (1H, app-s, H-3), 4.05 (1H, dd, partially obscured, J 6.7 Hz, H-6), 3.84 (1H, dd, $J_{H-4,H-5}$ 7.1 Hz, J 4.3 Hz, H-4), 3.81 (1H, dd, J 6.9 Hz, H-6′), 3.20-3.00 (1H, br s, OH), 1.52, 1.42, 1.35, 1.32 (12H, 4 x s, 4 x CH_3) and whose characterisation data matched the literature (Table 1, **entry 3**).

Table S1. (*top*) Structures, (*middle*) ^{1}H- and (*bottom*) ^{13}C-NMR data in $CDCl_3$ for all stereoisomeric 1,2:5,6-di-*O*-isopropylidene-α-D-aldohexofuranosides at C-3 and C-4: 1,2:5,6-di-*O*-isopropylidene-α-D-allofuranose (**entry 1**), 1,2:5,6-di-*O*-isopropylidene-α-D-galactofuranose (**entry 2**), 1,2:5,6-di-*O*-isopropylidene-α-D-glucofuranose (**entry 3**), 1,2:5,6-di-*O*-isopropylidene-α-D-gulofuranose (**entry 4**). Chemical shifts are in ppm and *J* coupling values in Hz.

**Entry**	**1** [27-29]	**2** [30-32]	**3** [27-29, 33]	**4**
**Structure**	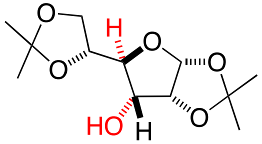 **D-Allo**	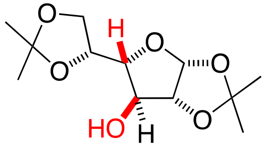 **D-Galacto**	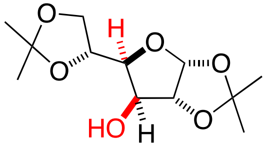 **D-Gluco**	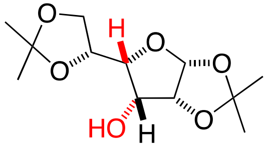 **D-Gulo**

**Entry**	**H-1**	**H-2**	**H-3**	**H-4**	**H-5**	**H-6**	**H-6'**	**OH**	**4 x CH$_3$**	**References**
**1**	5.80, d<br>$J_{H-1,H-2}$ 3.8	4.59, dd<br>$J_{H-2,H-3}$ 5.1<br>$J_{H-2,H-1}$ 4.0	4.08-3.97, m		4.29, ddd<br>$J_{H-5,H-6/H-4}$ 6.6<br>$J_{H-5,H-6'}$ 4.9	4.08-3.97, m	3.80,dd<br>$J_{H-6',H-6}$ 8.5<br>$J_{H-6',H-5}$ 4.8	2.55, d<br>$J_{OH,H-3}$ 8.4	1.56, 1.45, 1.36, 1.35	[28]
	5.82, d<br>$J_{H-1,H-2}$ 3.8	4.62, dd<br>$J_{H-2,H-1}$ 3.9	4.11-4.00, m		4.31, m	4.11-4.00, m	3.82, dd<br>$J_{H-6',H-6}$ 8.4<br>$J_{H-6',H-5}$ 4.8		1.58 ,1.47, 1.38	Our data§
**2**	5.80, d<br>$J_{H-1,H-2}$ 4.2	4.49, d<br>$J_{H-2,H-1}$ 4.2	4.05-3.98, m	3.81-3.75, m	4.30, m	4.05-3.98, m	3.81-3.75, m		1.48, 1.39, 1.31, 1.28	[34]
	5.85, d<br>$J_{H-1,H-2}$ 3.8	4.52, d<br>$J_{H-2,H-1}$ 3.8	4.08-4.05, app-s	3.84, dd^{ϖ}<br>$J_{H-4,H-5}$ 7.1,<br>J 4.3	4.33, app-q<br>J 6.9	4.05, dd^{ϖ}<br>J 6.7$^{\phi}$	3.81, dd<br>J 6.9$^{\phi}$	3.20-3.00, br s	1.52, 1.42, 1.35, 1.32	Our data
**3**	5.94, d<br>$J_{H-1,H-2}$ *3.6*	4.53, d<br>$J_{H-2,H-1}$ 3.6	4.37-4.31, m	4.07, dd<br>J 7.7<br>J 2.9	4.37-4.31, m	4.17, dd<br>$J_{H-6,H-6'}$ 8.6<br>$J_{H-6,H-5}$ 6.3	3.98, dd<br>$J_{H-6',H-6}$ 8.6<br>$J_{H-6',H-5}$ 5.4	2.60-2.54, br s	1.49, 1.44, 1.36, 1.32	Our data
**4**	5.73, d<br>$J_{H-1,H-2}$ 4.1	4.61, dd<br>$J_{H-2,H-1}$ 4.1<br>$J_{H-2,H-3}$ 6.3	4.22-4.14, m	3.85, dd<br>$J_{H-4,H-5}$ 8.7<br>$J_{H-2,H-3}$ 5.9	4.44, dt<br>$J_{H-5,H-4}$ 8.6<br>$J_{H-5,H-6/6'}$ 7.0	4.22-4.14, m	3.66, dd<br>$J_{H-6,H-5}$ 8.6<br>$J_{H-6,H-6'}$ 7.4	2.68, d<br>$J_{OH,H-3}$ 6.4	1.58, 1.40, 1.37, 1.33	[35]
	5.78, d<br>$J_{H-1,H-2}$ 4.0	4.66, dd<br>$J_{H-2,H-3}$ 6.3<br>$J_{H-2,H-1}$ 4.1	4.25-4.19, m	3.90, dd<br>$J_{H-4,H-5}$ 8.5<br>$J_{H-4,H-3}$ 5.7	4.47, dt<br>$J_{H-5,H-4}$ 8.4<br>$J_{H-5,H-6}$ 7.1<br>$J_{H-5,H-6'}$ 7.0	4.25-4.19, m	3.72, dd<br>$J_{H-6,H-6'}$ 8.5<br>$J_{H-6,H-5}$ 7.3	2.65, d<br>$J_{OH-3,H-3}$ 6.5	1.63, 1.45, 1.43, 1.38	Our data

**Entry**	**C-1**	**C-2**	**C-3**	**C-4**	**C-5**	**C-6**	**2 x C$_{quat}$**	**4 x CH$_3$**	**References**
**1**	103.9	79.7	75.6	79.1	72.4	65.8	*NA*	*NA*	[36, 37]
	104.0	79.6	*72.4*#	79.1	*75.4*#	65.7	112.6, 109.6	26.4, 25.3	[38]
	103.7	79.6	78.8	75.5	72.4	65.7	112.6, 109.6	26.5, 26.4, 26.2, 25.2	[28]

	104.0	79.8	79.0	75.6	72.5	65.9	112.9, 109.8	26.6, 26.5, 26.3, 25.3	Our data[§]
**2**	104.9	87.6	76.1	85.9	75.3	65.6	113.6, 109.9	27.4, 26.8, 26.5, 25.2	[34]
	105.1	87.7	76.1	86.5	75.7	65.8	113.5, 110.0	27.4, 26.7, 26.6, 25.4	Our data
**3**	105.4	85.2	75.0	81.3	73.3	67.7	*NA*	*NA*	[36]
	104.5	85.0	73.1	80.9	72.2	66.0	110.5, 107.8	26.5, 26.0, 26.0, 25.2	[39]
	104.9	84.6	74.5	81.0	72.8	67.3	111.4, 109.2	26.5, 25.9, 25.0	[38]
	104.5	84.4	74.5	80.4	72.8	67.0	111.8, 109.0	25.9, 26.8, 26.2, 25.2	Our data.
**4**	105.5	80.0	69.8	84.5	75.7	66.5	115.2, 109.4	27.3, 26.8, 25.4	Our data

Legend: [#]Data for these swapped. [§]Data collected by our laboratory in other projects. [ϕ]Other *J* coupling is not discernible due to overlap. [ϖ]Partially obscured signal. *NA* = not available.

Trapping with a 1,2-diol. It was reasoned this type of trapping agent should afford a more stable boronate ester intermediate, but it should have still been possible to produce the corresponding boronic acid *in situ via* an acidic treatment that wouldn't also remove the acetonide protecting group. Trapping with esculetin and molecular sieves in DCM at r.t. did not afford the desired product, but an inseparable mixture. Trapping with ethylene glycol provided the desired C-3 boron glycol ester, however could not be purified via re/crystallisation. Isolation by flash column chromatography of the desired product (R_f 0.00-0.10) provided yields in the single digits, likely due to interactions between silica gel and B atoms, and, additionally, due to potential decomposition of the product during purification. To give the deprocted boronic acid 3-boronic-3-deoxy-1,2:5,6-di-*O*-isopropylidene-α-D-galactofuranose δ_H ($CDCl_3$, 400 MHz): 5.77 (1H, d, $J_{H\text{-}1,H\text{-}2}$ 3.9 Hz, H-1), 4.81 (1H, dd, $J_{H\text{-}2,H\text{-}3}$ 2.2 Hz, $J_{H\text{-}2,H\text{-}1}$ 3.7 Hz, H-2), 4.42 (1H, app-q, $J_{H\text{-}5,H\text{-}4}$ 7.1 Hz, H-5), 4.12 (1H, dd, $J_{H\text{-}4,H\text{-}5}$ 7.1 Hz, $J_{H\text{-}4,H\text{-}3}$ 5.7 Hz, H-4), 4.03 (1H, dd, $J_{H\text{-}6,H\text{-}6'}$ 8.2 Hz, $J_{H\text{-}6,H\text{-}5}$ 6.6 Hz, H-6), 3.71 (1H, dd, $J_{H\text{-}6',H\text{-}6}$ 8.1 Hz, $J_{H\text{-}6',H\text{-}5}$ 7.1 Hz, H-6'), 2.20-1.70 (2H, broad singlet, 2 x OH), 1.69 (1H, dd, $J_{H\text{-}3,H\text{-}4}$ 5.4 Hz, $J_{H\text{-}3,H\text{-}2}$ 1.9 Hz, H-3), 1.57, 1.44, 1.36, 1.32 (12Hs, 4 x s, 4 x CH_3); δ_c ($CDCl_3$, 100 MHz): 113.2, 110.1 (2 x C_{quat} acetonides), 106.6 (C-1), 83.2, 83.1 (C-4, C-2), 77.9 (C-5), 66.2 (C-6) 32.0-30.0 (broad, C-3), 27.7, 26.7 [2 x C], 25.5 (4 x CH_3)]; δ_B ($CDCl_3$, 128 MHz): 33.3. (Supplementary Information, Figure S1)

Removal of the excess borane through addition of excess glycol produced boronate-glycol educts that were difficult to remove completely through attempted recrystallisation, crystallisations or partitioning. For example, using solvent mixtures such as diethyl ether/hexane did not yield any crystalline product or precipitate. We noticed that these boronated educts and boronated monosaccharide derivative tend to be oils with a very large solubility window, ranging from very unipolar (hexane) to very polar (water, methanol).

Trapping of the borane with a more hindered 1,2-diol to form a more stable boronate educt was attempted with pinacol to give product **5** (Scheme 1). This reaction was successful, however purification produced a mixture of D-*galacto* and D-*allo* boronated products and side-products. The boronated products could not be synthesised or isolated reliably. Various purification methods were attempted including by flash column chromatography and prep t.l.c. (with the products were visualised through sprinkling of iodine-soaked silica gel on the plate). The side-products included the saturated 1,2:5,6-di-*O*-isopropylidene-3-deoxy derivative mentioned earlier and oxidised products, such as 1,2:5,6-di-*O*-isopropylidene-α-D-galactofuranose and 1,2:5,6-di-*O*-isopropylidene-α-D-allofuranose.

It was attempted to crystallise the excess pinacol out *via* a variety of solvent mixtures covering the polarity range, but unsuccessfully. Boiling off of the pinacol also proved challenging due to its high boiling point (172°C). A further purification protocol utilising a complexation resin[40] also did not afford pure product. Therefore it was deemed appropriate to change the trapping agent.

1.3. Purification protocol optimisation (Table 2)

In Purification 1, the reaction mixture (200 mg scale) was filtered to remove excess DEA, however the filtrate appeared to contain product **6** along with some remaining excess DEA. The filtrate was dried and triturated with diethyl ether. This showed a slightly more pure product **6** (the solid fraction), while ^{1}H-NMR indicated unidentified compound mixture in the filtrate. An additional trituration with PET ether/diethyl ether (5:1) removed impurities which ended in the filtrate, although the excess DEA remained still largely trapped in the solid fraction. Finally product **6** was dissolved in DCM and partitioned with HCl (0.1 M). The excess DEA was successfully removed and product **6** was the sole species migrating to the DCM layer (23% yield).

Table S2. Summary of the purification methods employed in the optimisation of the purification protocol to product **6.**

Purification	Purification Method	Outcome	Yield
1	Filtration of crude (crude contained **6**:DEA in 1.0:1.6 ratio)	Filtrate: **6** and DEA (1.0:0.8 ratio)<br>Solid: mainly DEA and unidentified compounds.	23%
	Diethyl ether trituration	Filtrate: unidentified compounds.<br>Solid: **6** and DEA (1.0: 0.6 ratio).	
	PET ether/diethyl ether (5:1) partition	Filtrate: unidentified compounds.<br>Solid: **6** and DEA (1.0: 0.6 ratio).	
	DCM/HCl (0.1 M) wash	DCM layer: pure **6**.<br>Aqueous layer: small amount of 6 and DEA (1.0:2.8 ratio).	
2	DCM/HCl (0.1 M) partition 1	DCM layer: **6** and DEA (1.0:1.9 ratio).	23%
	DCM/HCl (0.1 M) partition 2	DCM layer: **6** and DEA (1.0:0.7 ratio) and unidentified impurities.	
	DCM/HCl (0.1 M) partition 3	DCM layer: **6** and DEA (1.0:0.6 ratio) and unidentified compounds.	
	Diethyl ether trituration	Solid: pure **6**.<br>Filtrate: unidentified compounds.	
3	Filtration of crude	Filtrate: **6** and some DEA.<br>Solid: mainly DEA and unidentified compounds.	58%
	Diethyl ether trituration	Filtrate: unidentified compounds.<br>Solid: **6** and DEA.	
	DCM/HCl (0.1 M) partition	DCM layer: pure **6**.	

As the yield for purification method 1 was quite low, the second purification method focused on the successful DCM/HCl (0.1 M) partitioning. The reaction mixture (500 mg scale) was dried and then partitioned in DCM/HCl (0.1 M). Although the majority of the DEA was removed in this first partitioning, there was some remaining in the DCM layer along with unidentified compounds. After two additional HCl (0.1 M) washes, the DCM layer still contained unidentified compounds, although a majority of the DEA had been successfully removed. The DCM layer was dried, triturated with diethyl ether and filtered to give pure product **6** (23%) as a white solid, with the unidentified compounds ending up in the filtrate.

2. NMR Spectra

Figure S1. ^{1}H- (400 MHz), ^{13}C-NMR (100 MHz), DEPT (100 MHz), ^{11}B-NMR (128 MHz), COSY and HMBC spectra of 3-boronic-3-deoxy-1,2:5,6-di-*O*-isopropylidene-α-D-galactofuranose **8** in $CDCl_3$.

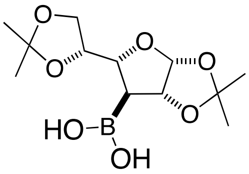

8
D-*Galacto*

^{1}H-NMR

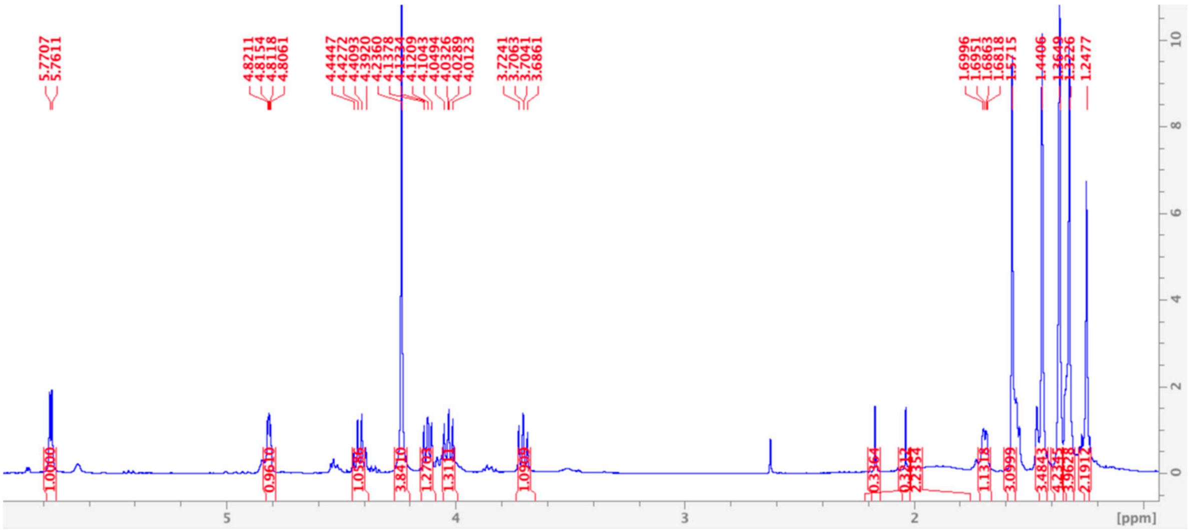

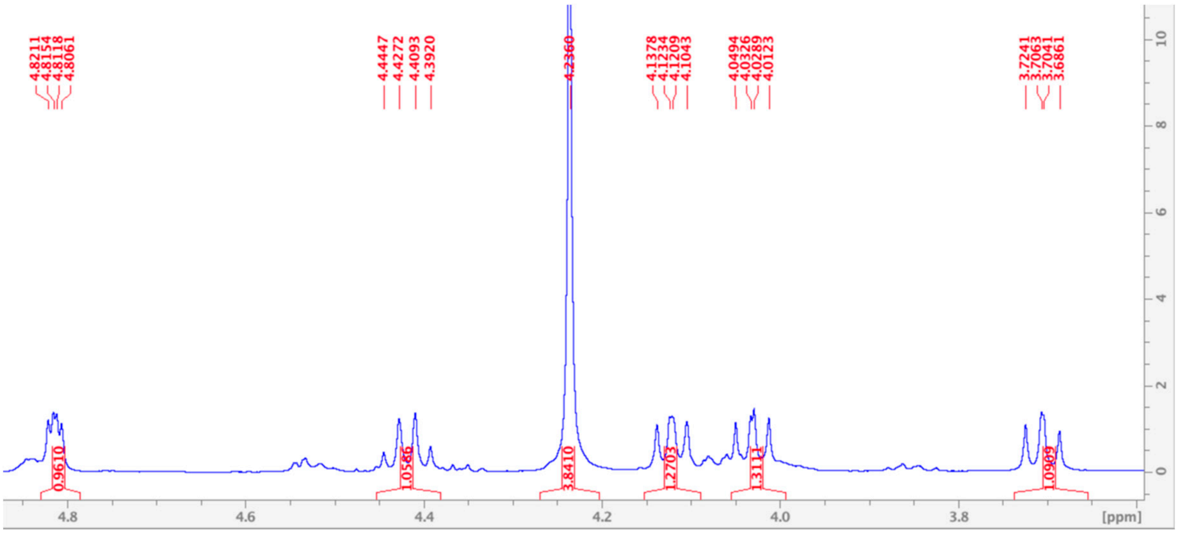

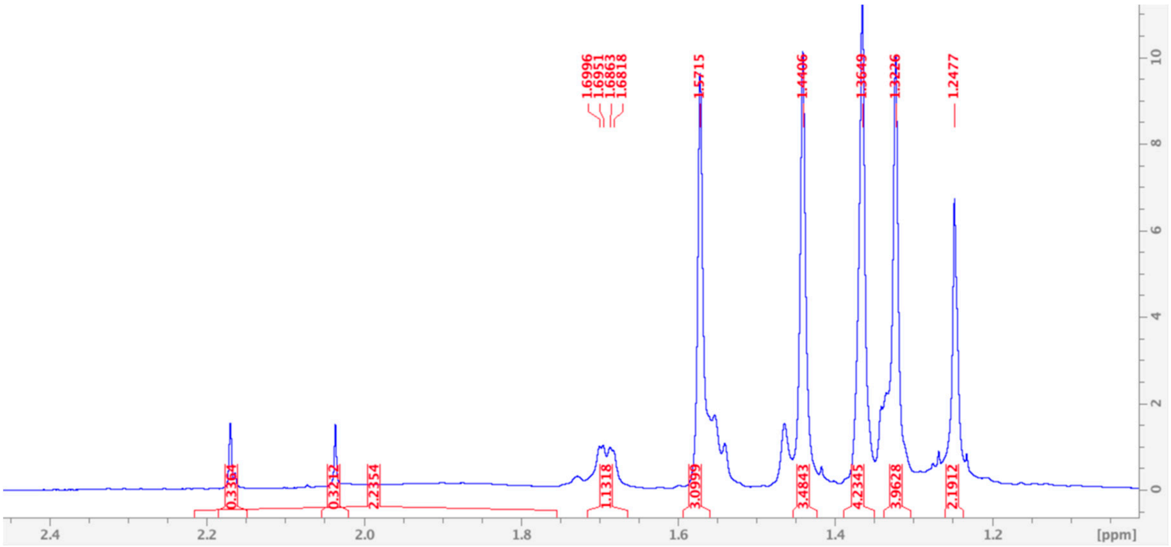

Note, impurity at 4.24 ppm and 1.25 ppm in the 1H-NMR spectrum, HMQC-coupled to signals at 62.3 ppm and 29.4 ppm in the 13C-NMR spectrum.

^{13}C-NMR

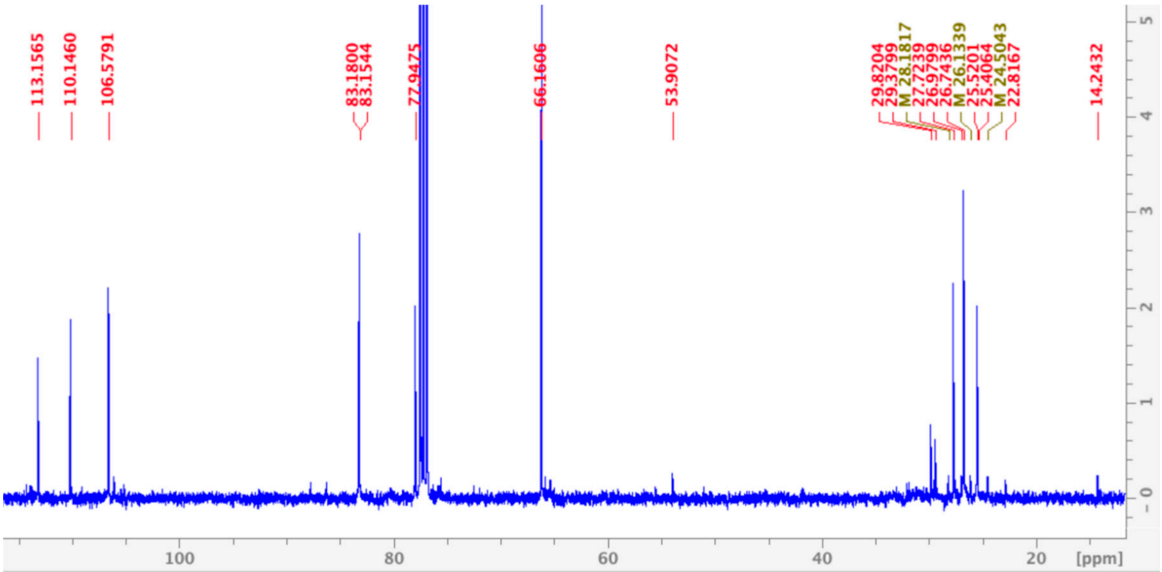

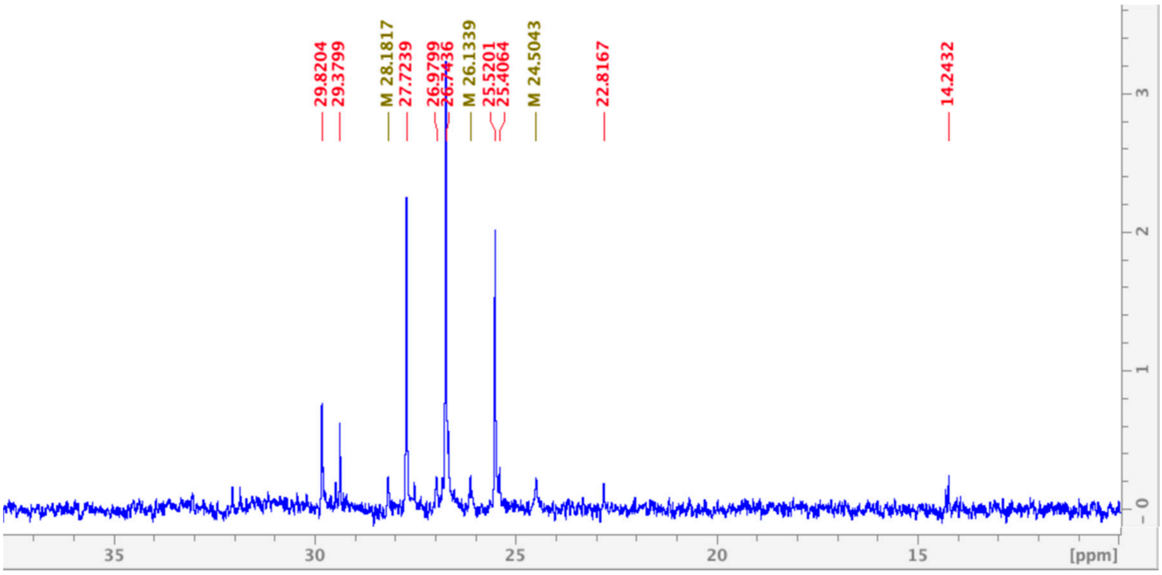

DEPT

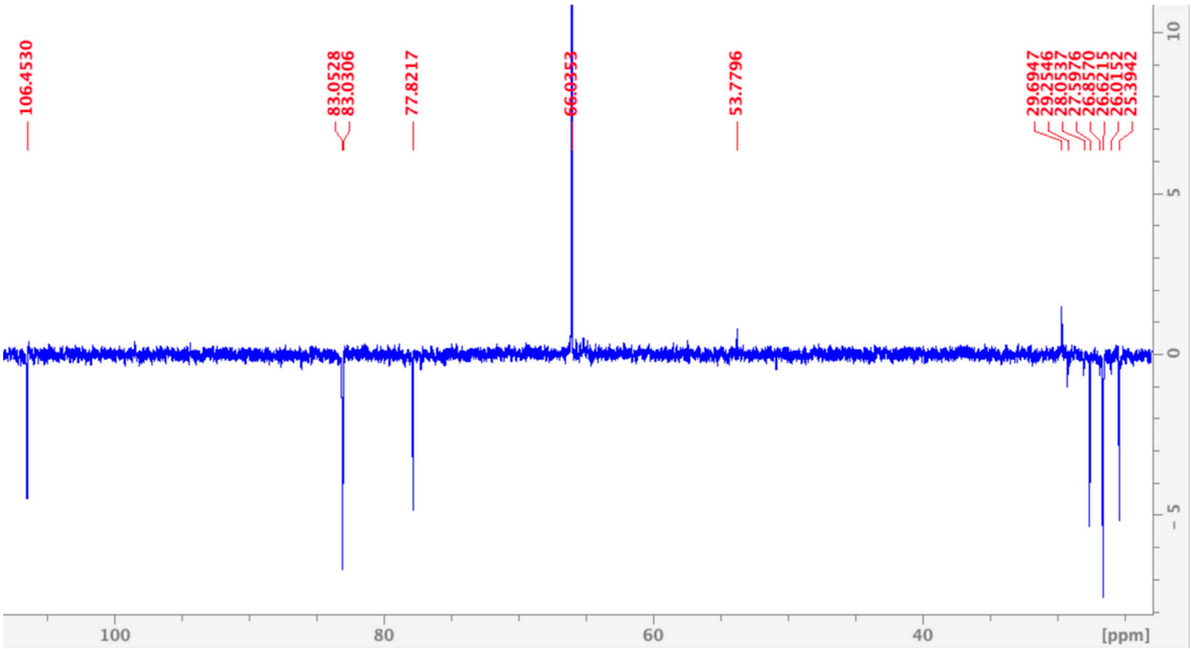
106.4530
83.0528
83.0306
77.8217
66.0353
53.7796
29.6947
29.2546
28.0537
27.5976
26.8570
26.6215
26.0152
25.3942
100
80
60
40
[ppm]
10
5
0
- 5

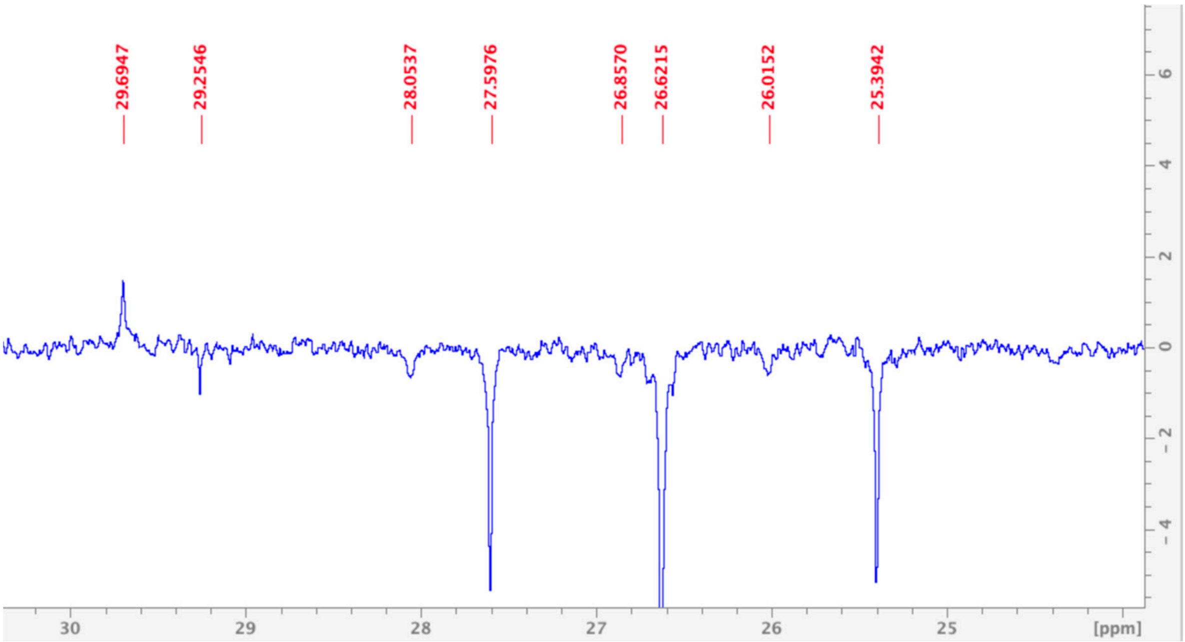
29.6947
29.2546
28.0537
27.5976
26.8570
26.6215
26.0152
25.3942
30
29
28
27
26
25
[ppm]
6
4
2
0
- 2
- 4

^{11}B-NMR

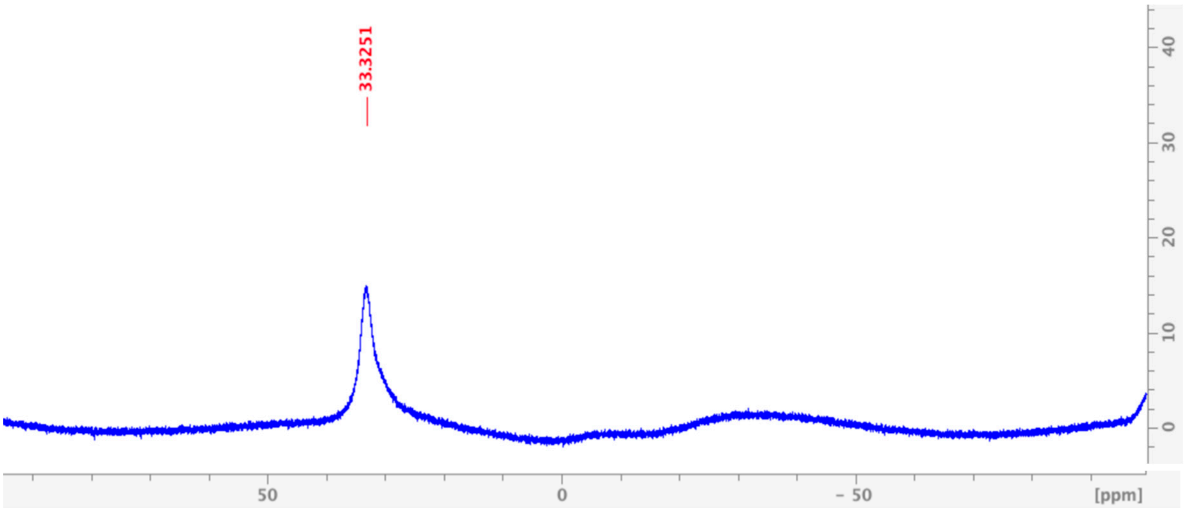

COSY

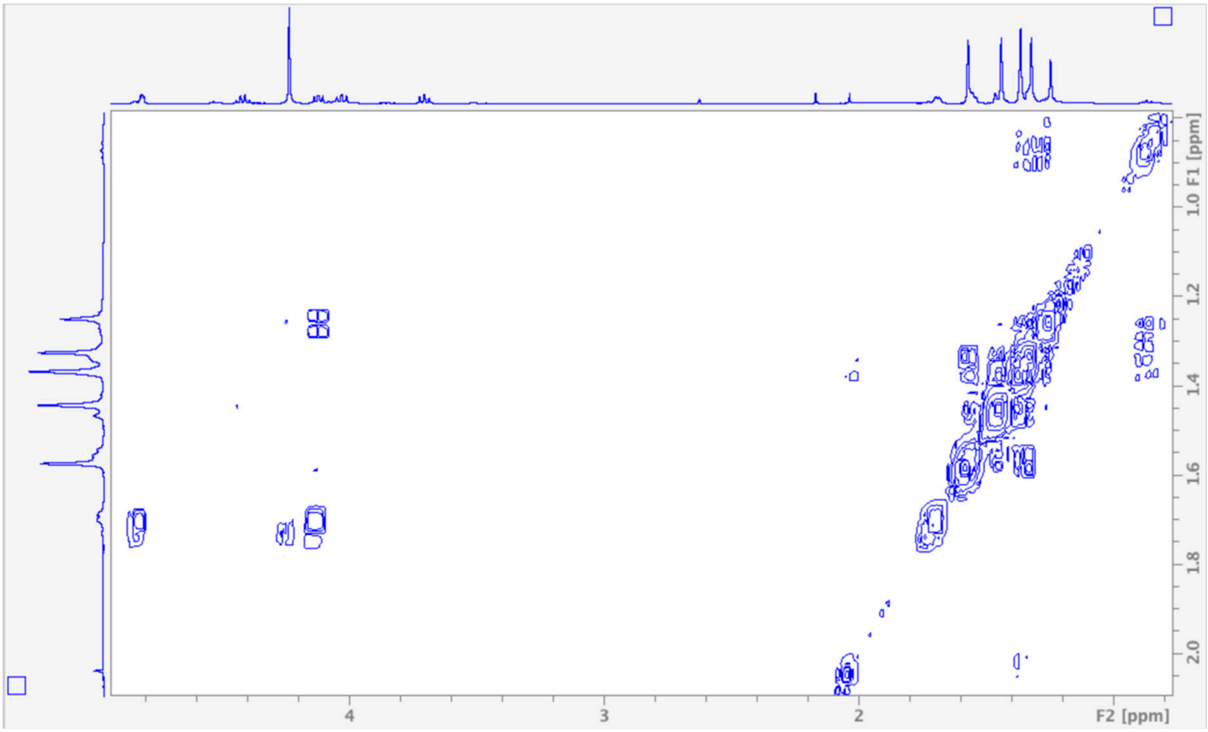

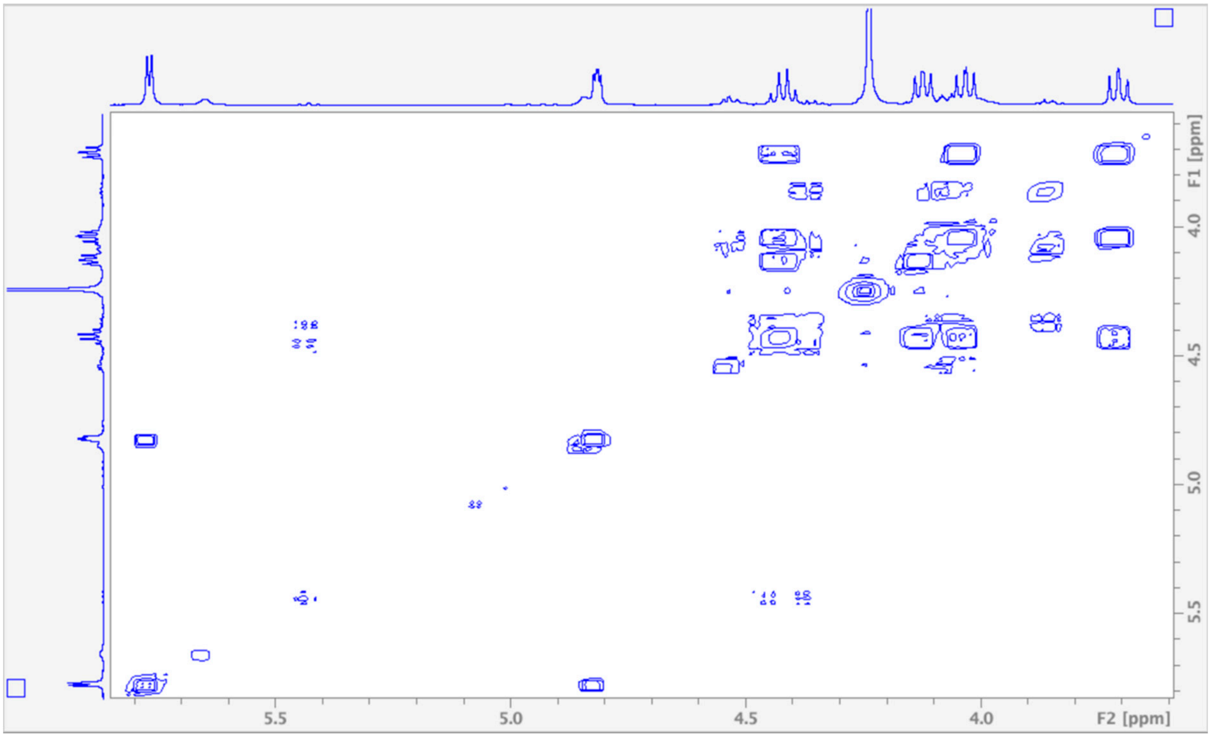
F1 [ppm]
4.0
4.5
5.0
5.5
5.5
5.0
4.5
4.0
F2 [ppm]

HMBC

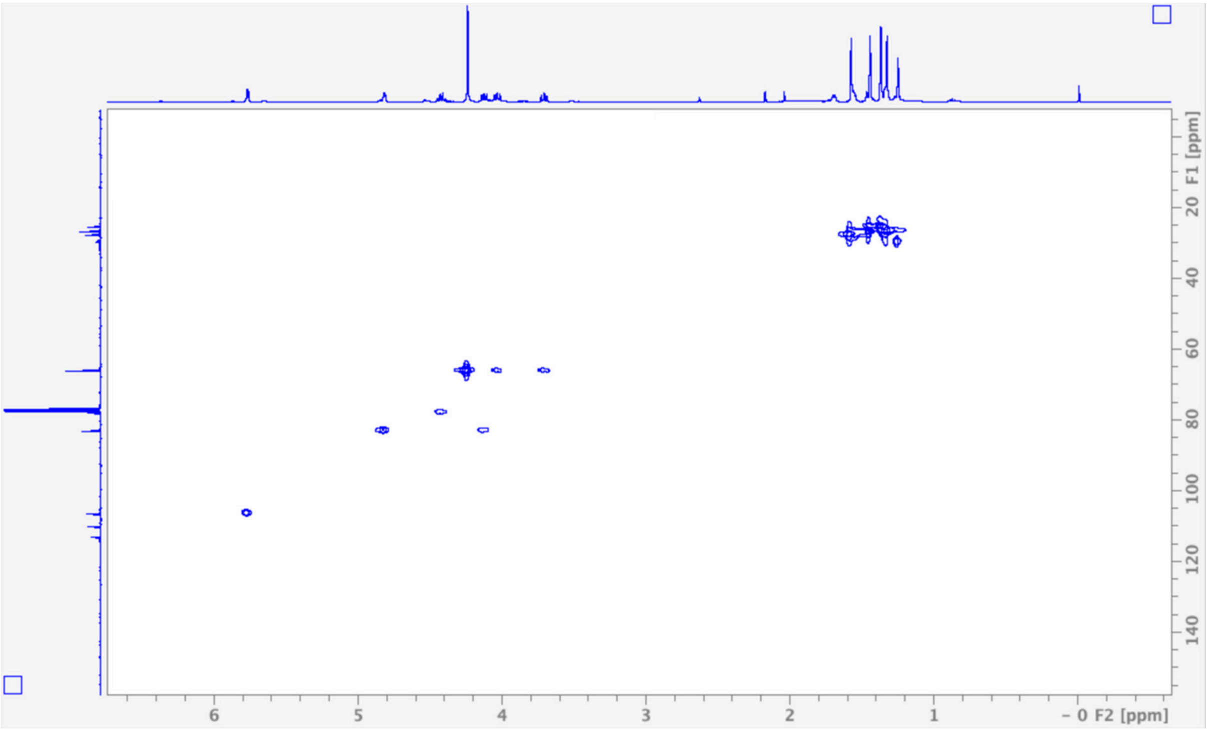
F1 [ppm]
20
40
60
80
100
120
140
6
5
4
3
2
1
0
F2 [ppm]

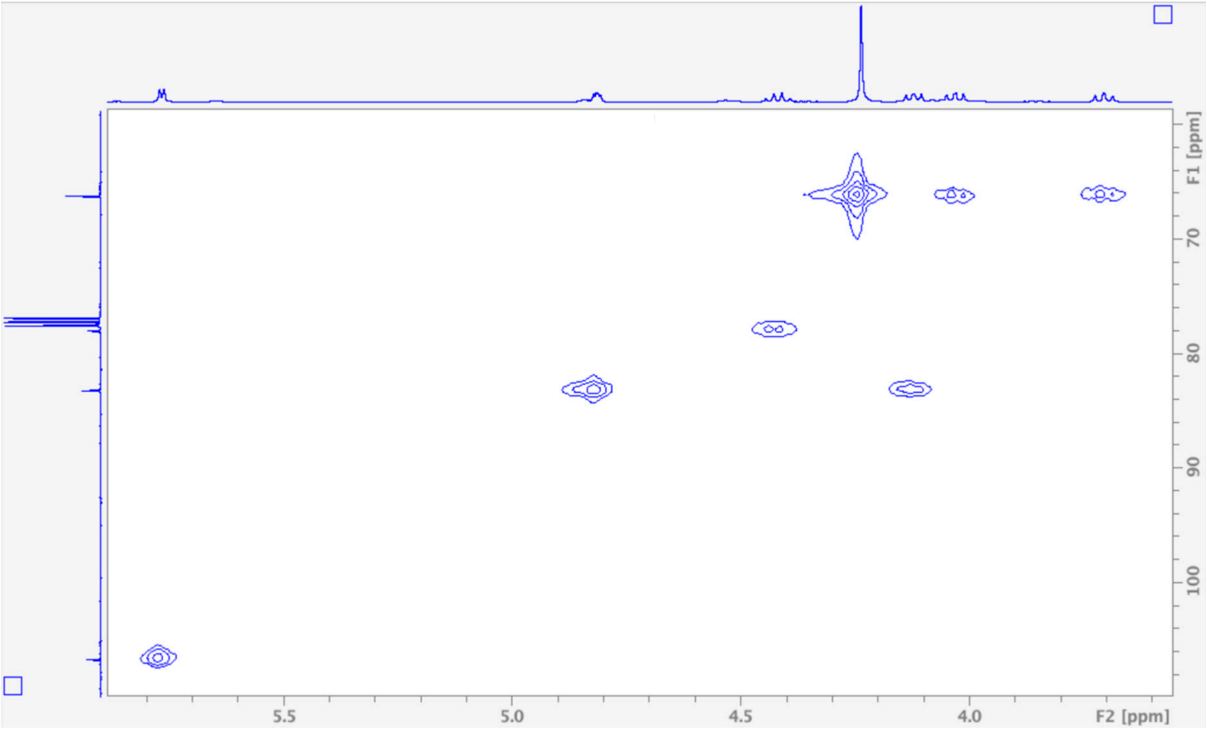
F1 [ppm]
70
80
90
100
5.5
5.0
4.5
4.0
F2 [ppm]

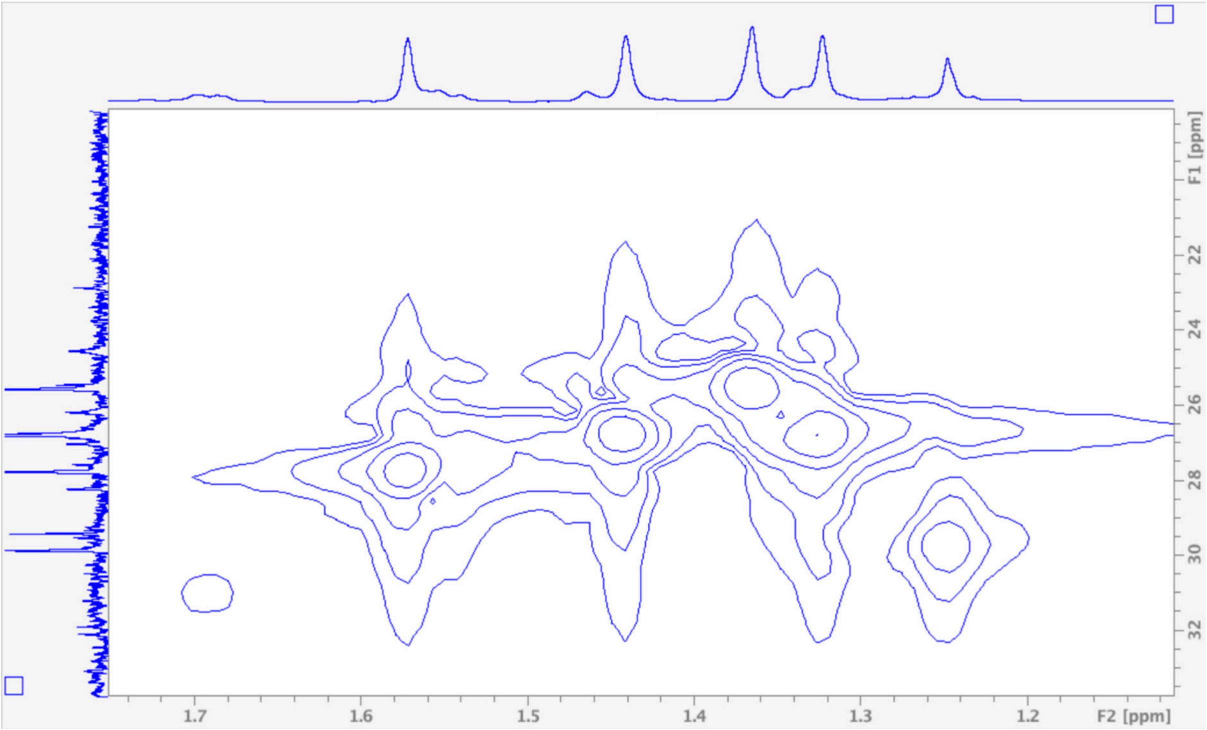
F1 [ppm]
22
24
26
28
30
32
1.7
1.6
1.5
1.4
1.3
1.2
F2 [ppm]

Figure S2. ^{1}H- (400 MHz), ^{13}C-NMR (100 MHz), DEPT (100 MHz), COSY and HSQC spectra of 1,2:5,6-Di-*O*-isopropylidene-α-D-gulofuranose in $CDCl_3$.

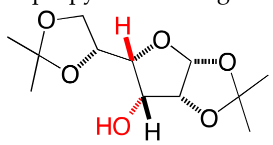

^{1}H-NMR

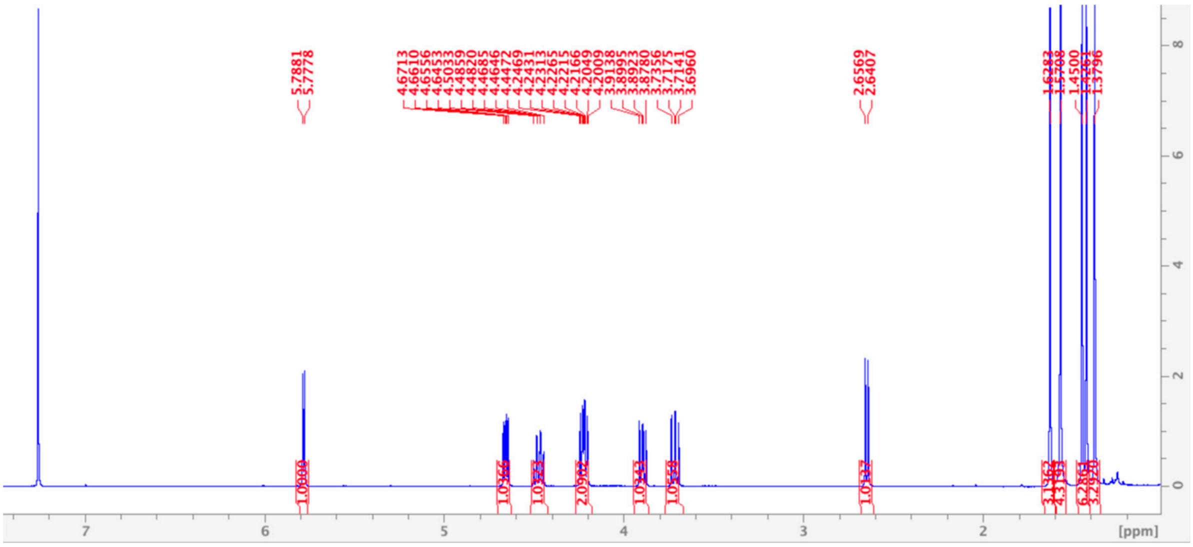

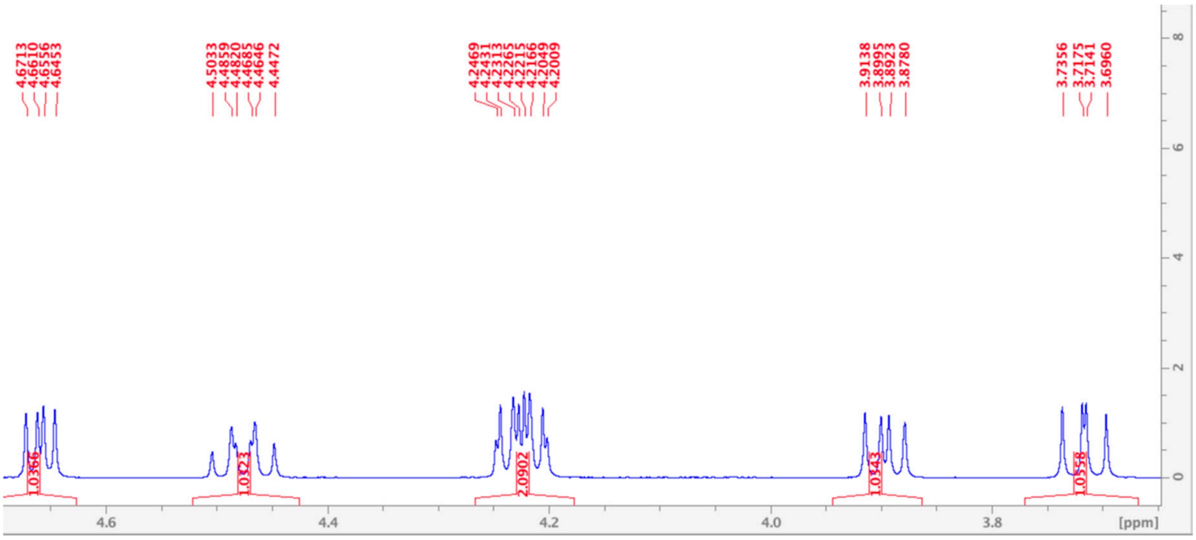

^{13}C-NMR

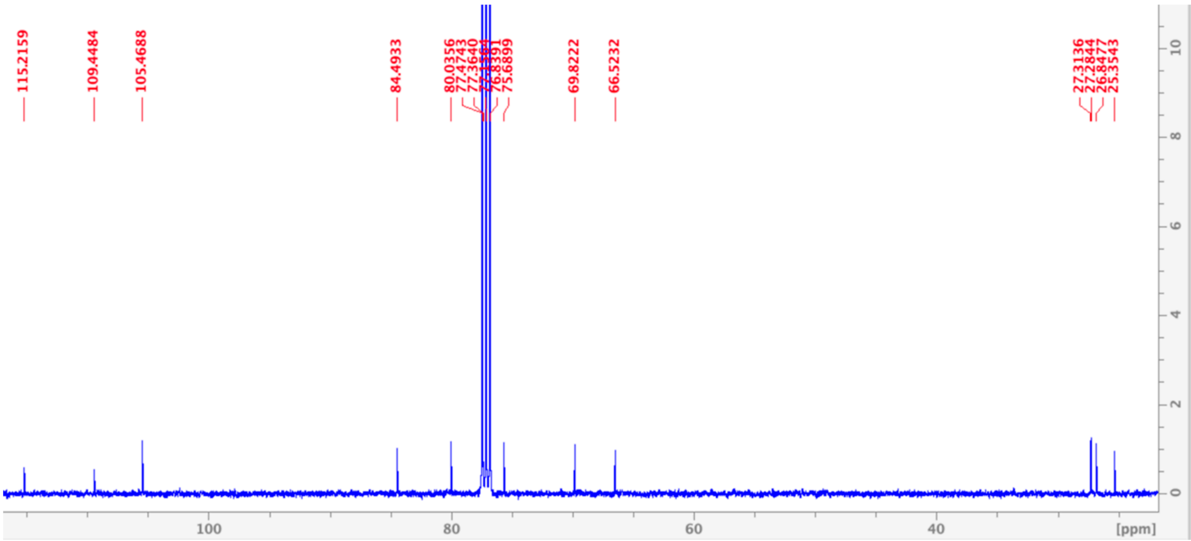
115.2159
109.4484
105.4688
84.4933
80.0356
77.4743
77.3640
69.8222
66.5232
27.3136
27.2844
26.8477
25.3543
[ppm]

DEPT

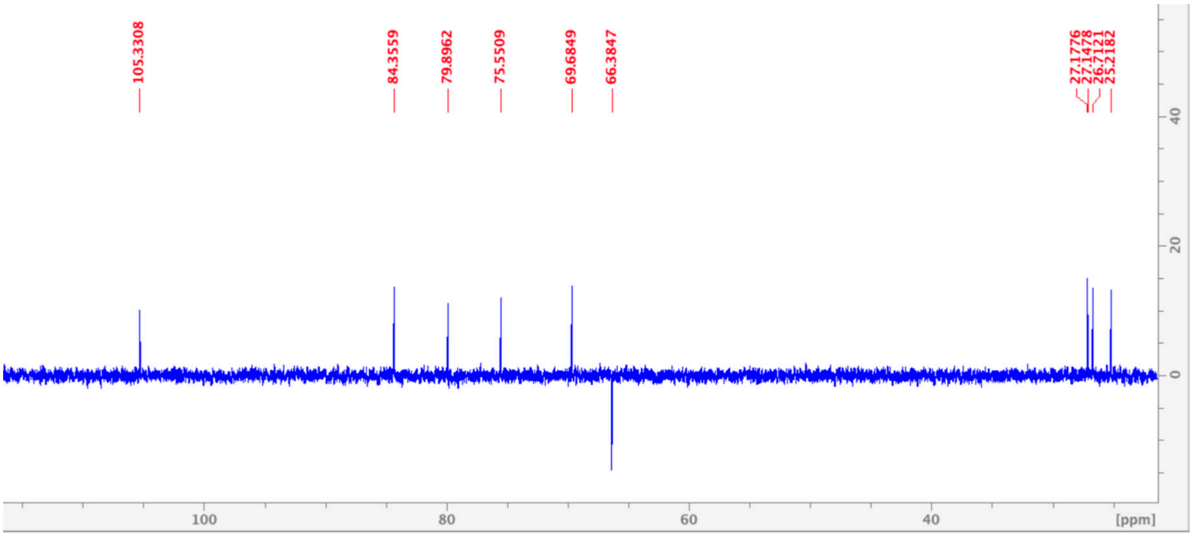
105.3308
84.3559
79.8962
75.5509
69.6849
66.3847
27.1776
27.1478
26.7121
25.2182
[ppm]

COSY

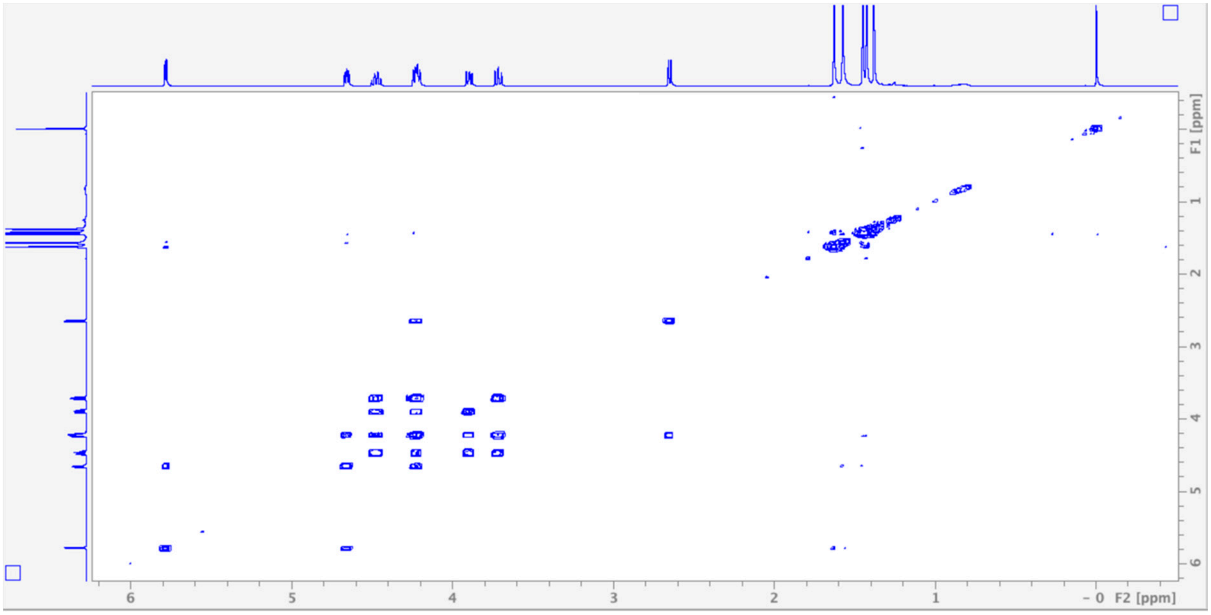

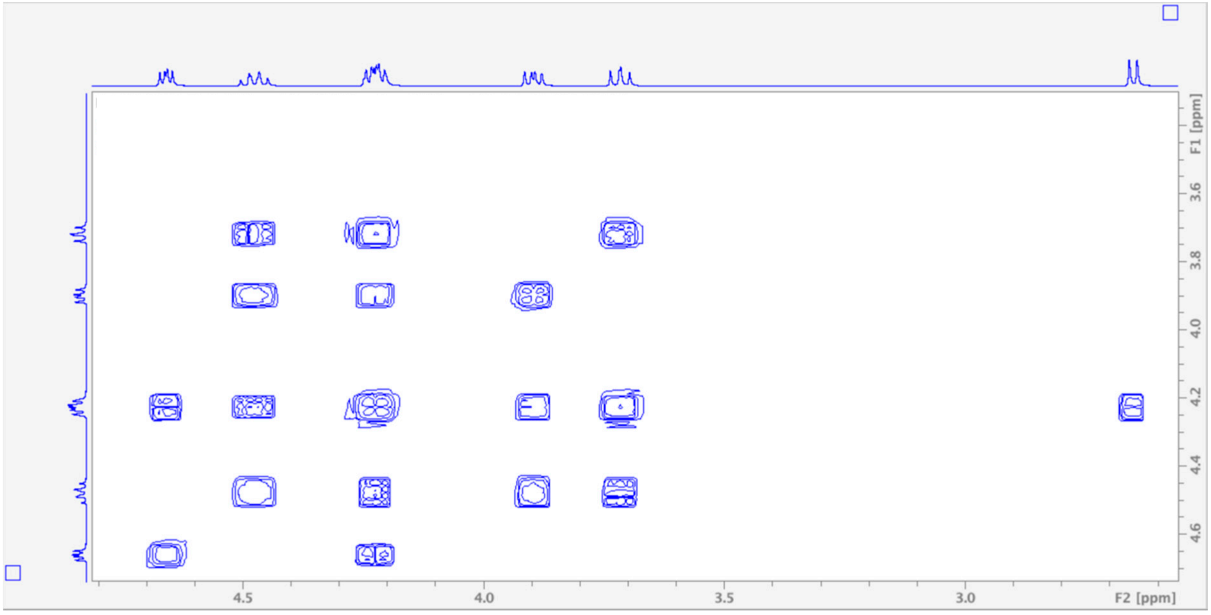

HSQC

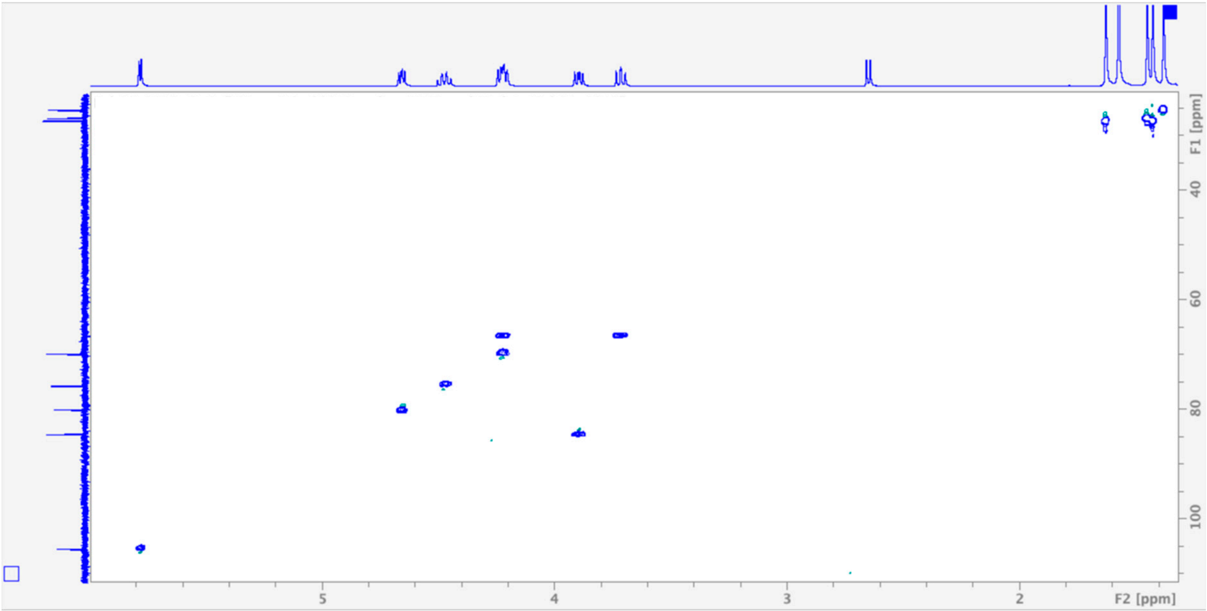

Figure S3. ^{1}H- (400 MHz), ^{13}C-NMR (100 MHz), DEPT (100 MHz), COSY, HMQC and HMBC spectra of 1,2:5,6-Di-*O*-isopropylidene-α-D-galactofuranose in $CDCl_3$.

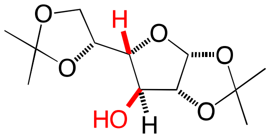

^{1}H-NMR

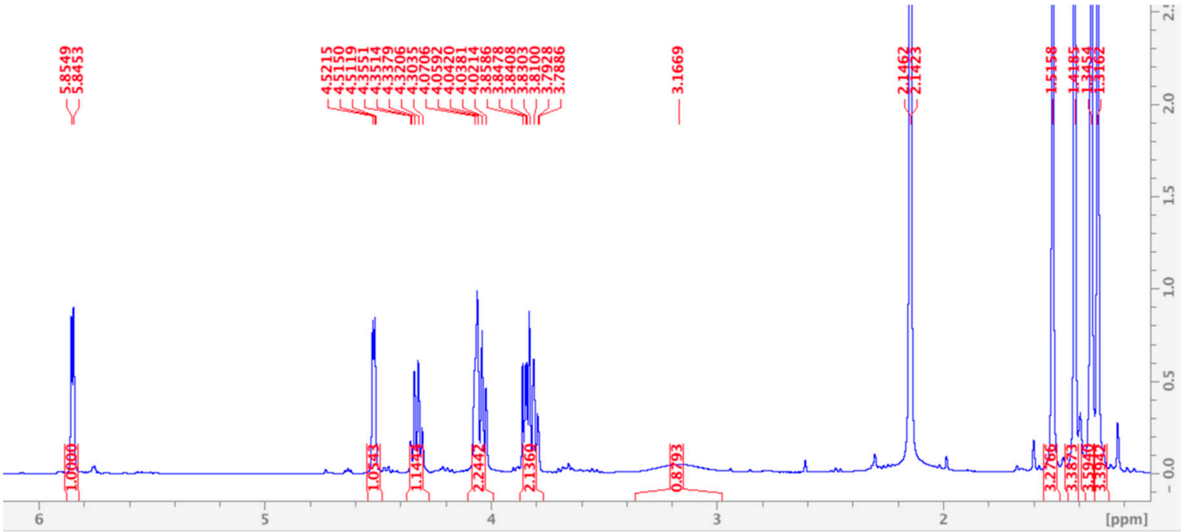

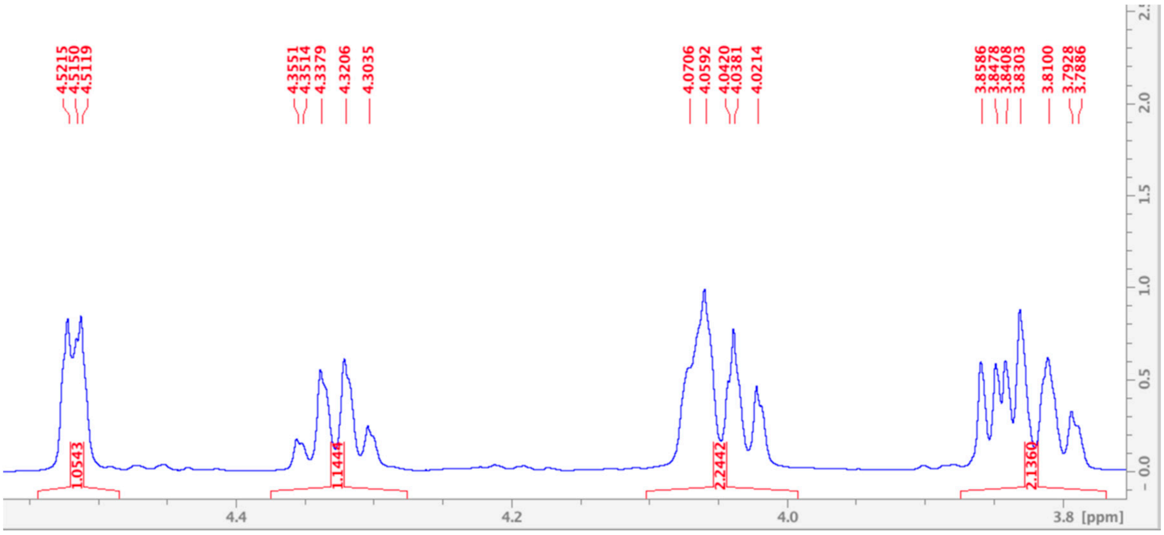

^{13}C-NMR

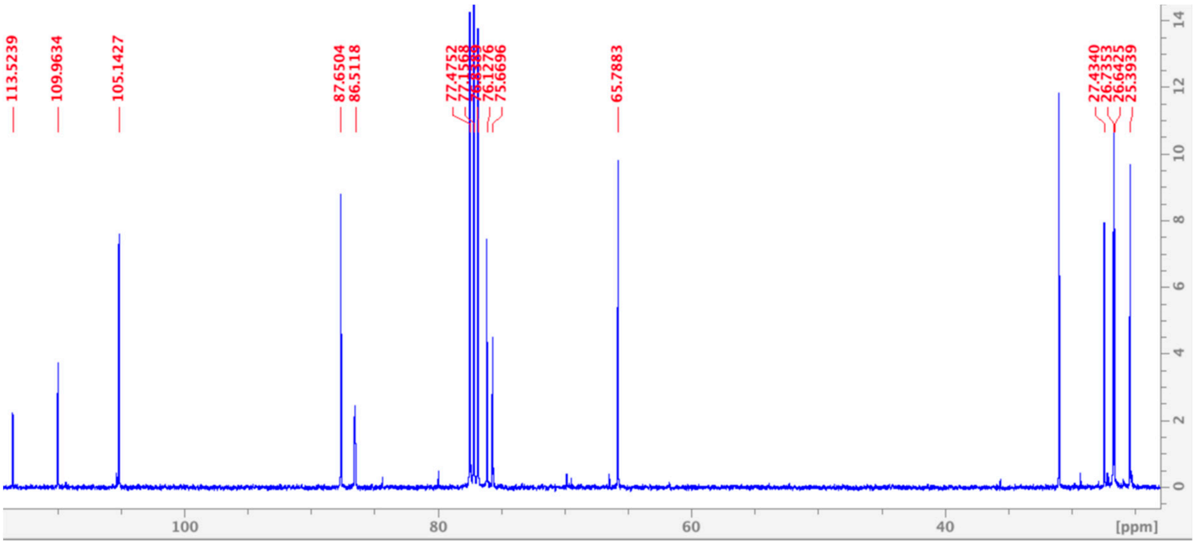
113.5239
109.9634
105.1427
87.6504
86.5118
77.4752
77.1568
76.8389
76.1276
75.6696
65.7883
27.4340
26.7353
26.6425
25.3939
100
80
60
40
[ppm]

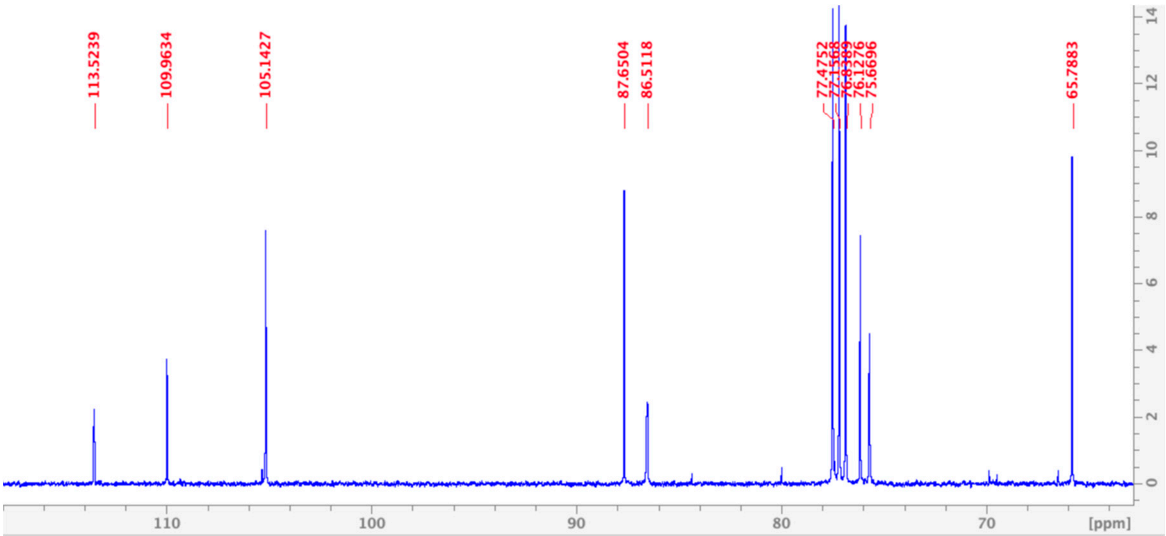
113.5239
109.9634
105.1427
87.6504
86.5118
77.4752
77.1568
76.8389
76.1276
75.6696
65.7883
110
100
90
80
70
[ppm]

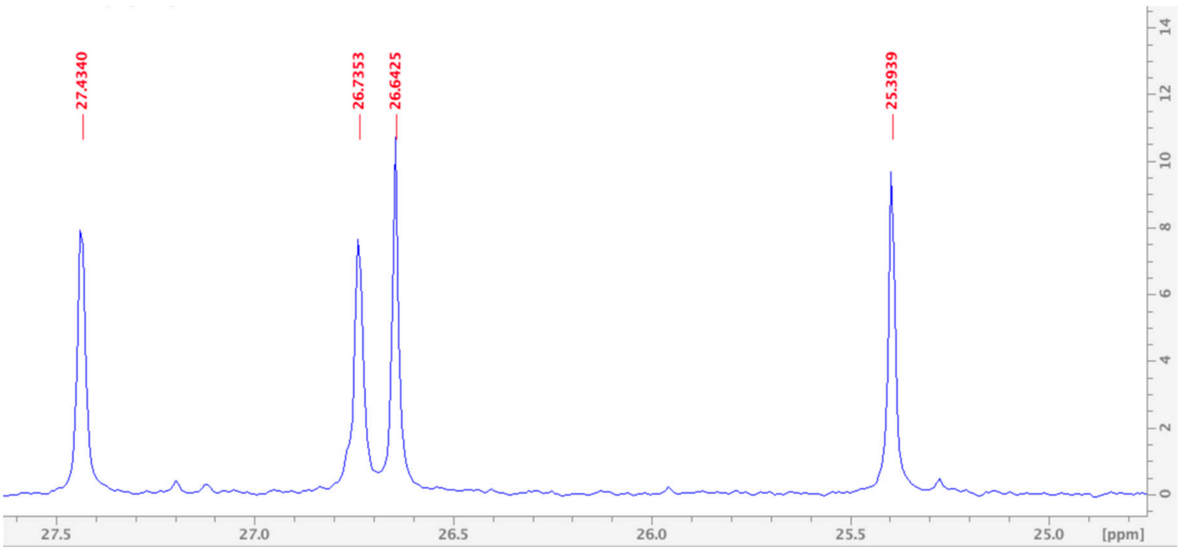
27.4340
26.7353
26.6425
25.3939
27.5
27.0
26.5
26.0
25.5
25.0
[ppm]

DEPT

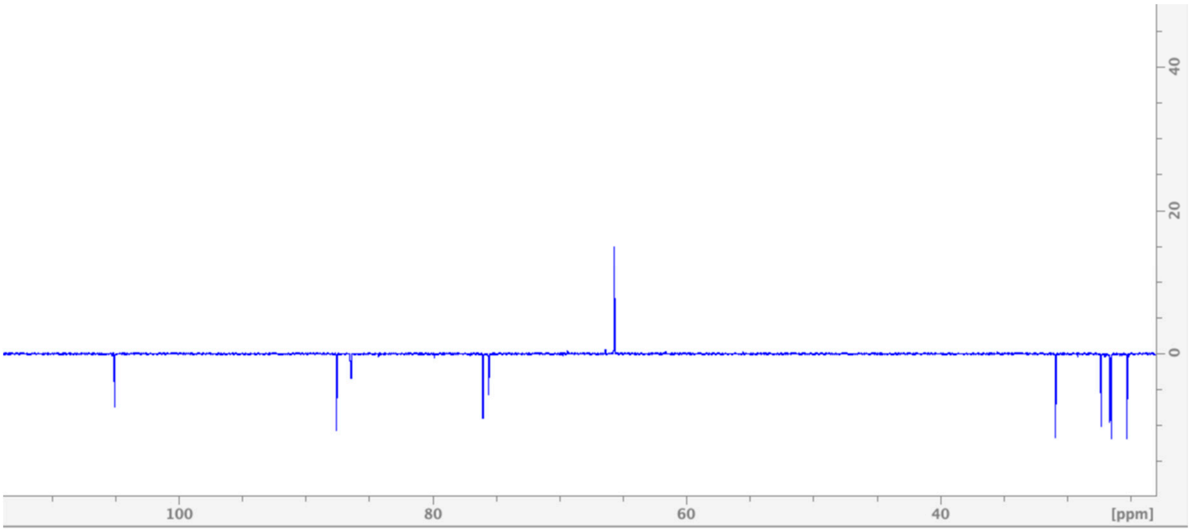

COSY

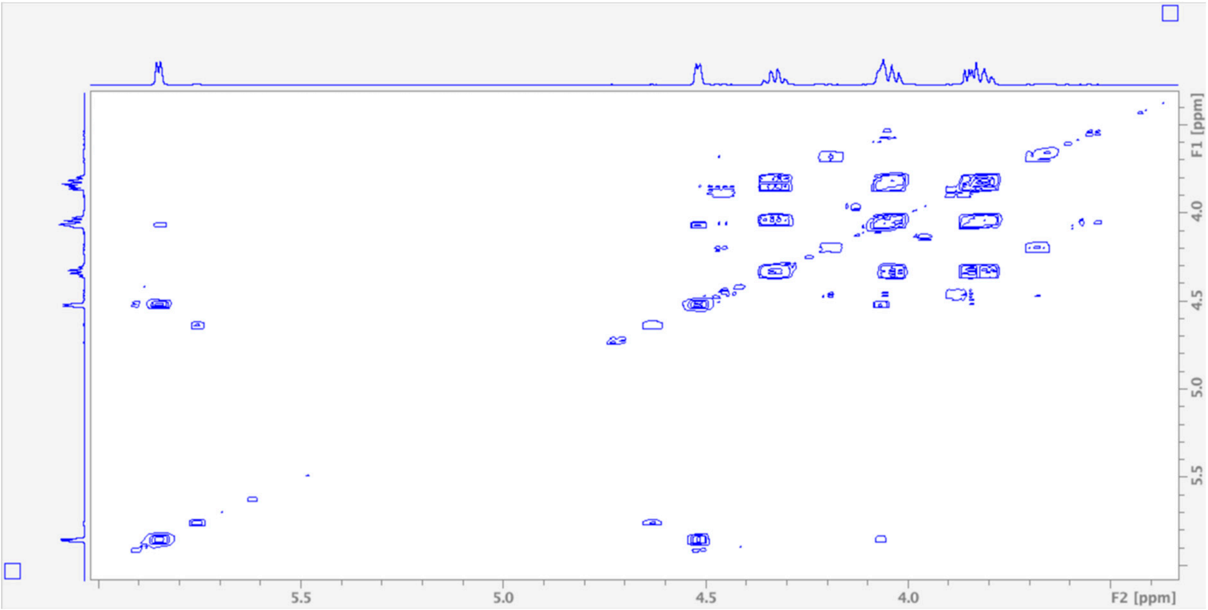

HMBC

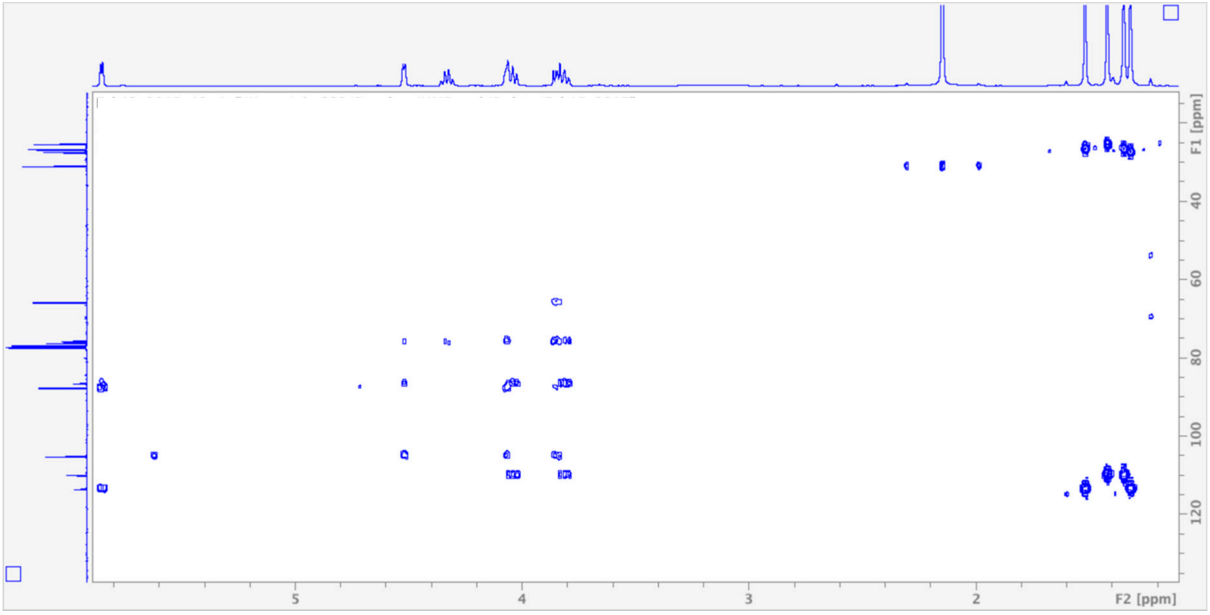
F1 [ppm]
40
60
80
100
120
5
4
3
2
F2 [ppm]

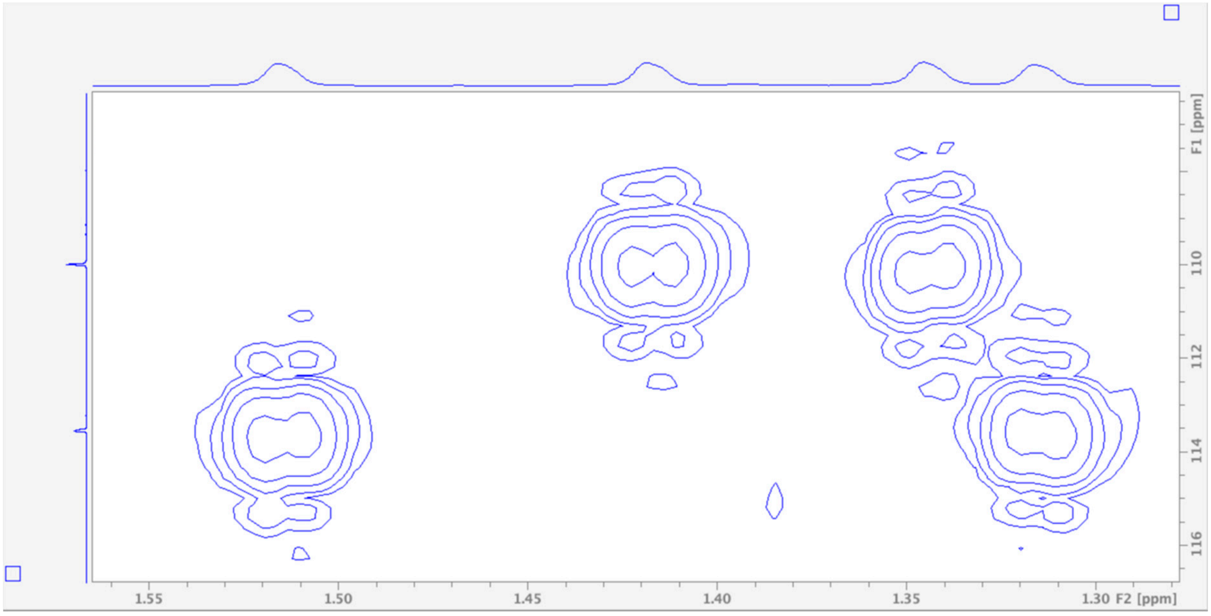
F1 [ppm]
110
112
114
116
1.55
1.50
1.45
1.40
1.35
1.30 F2 [ppm]

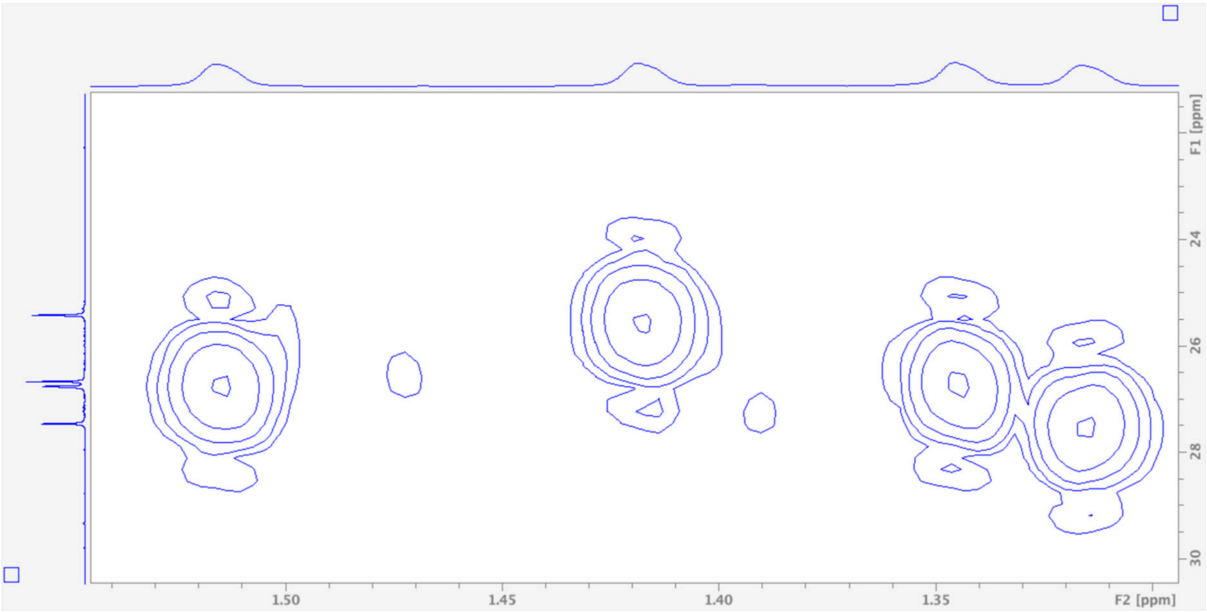

F1 [ppm]
24
26
28
30
1.50
1.45
1.40
1.35
F2 [ppm]

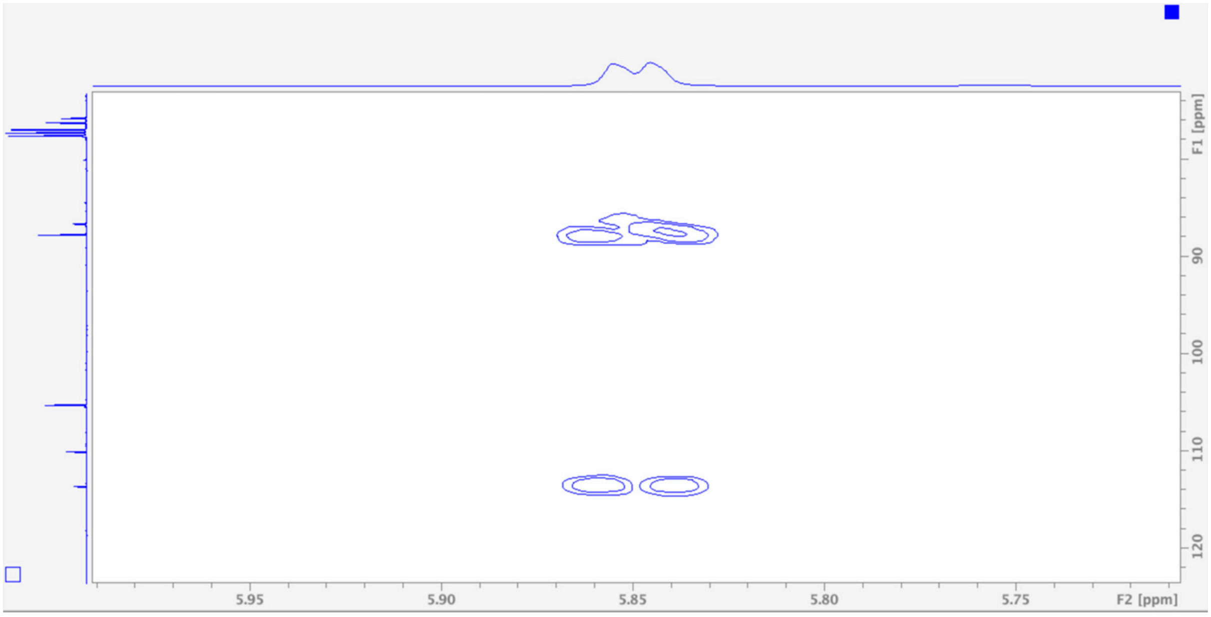

F1 [ppm]
90
100
110
120
5.95
5.90
5.85
5.80
5.75
F2 [ppm]

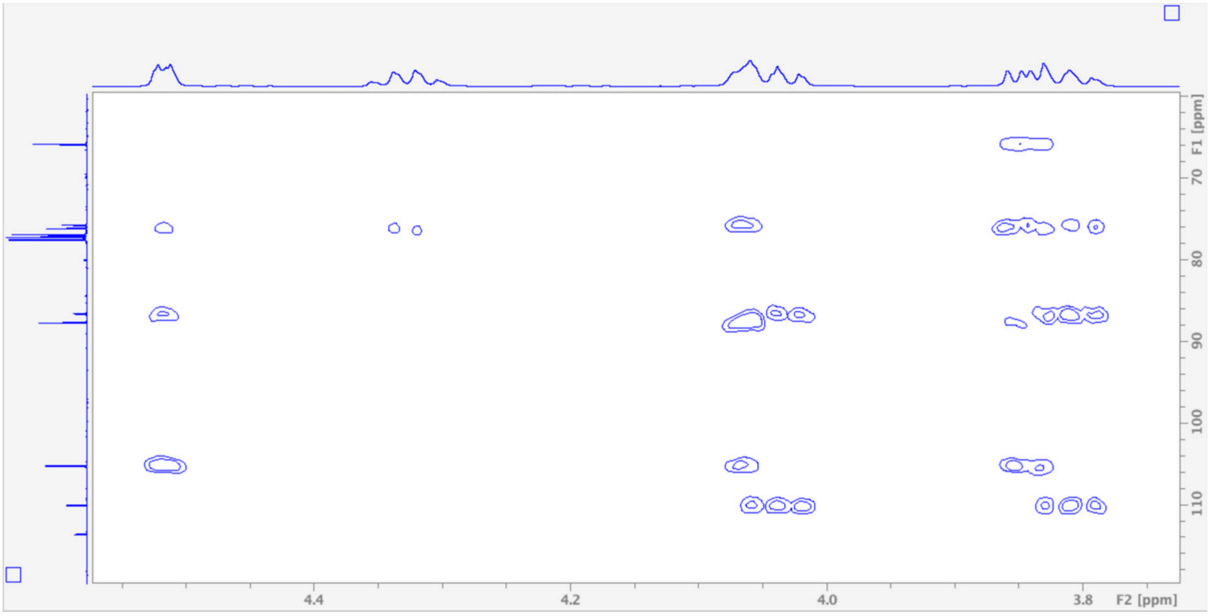
4.4
4.2
4.0
3.8
F2 [ppm]
F1 [ppm]
70
80
90
100
110

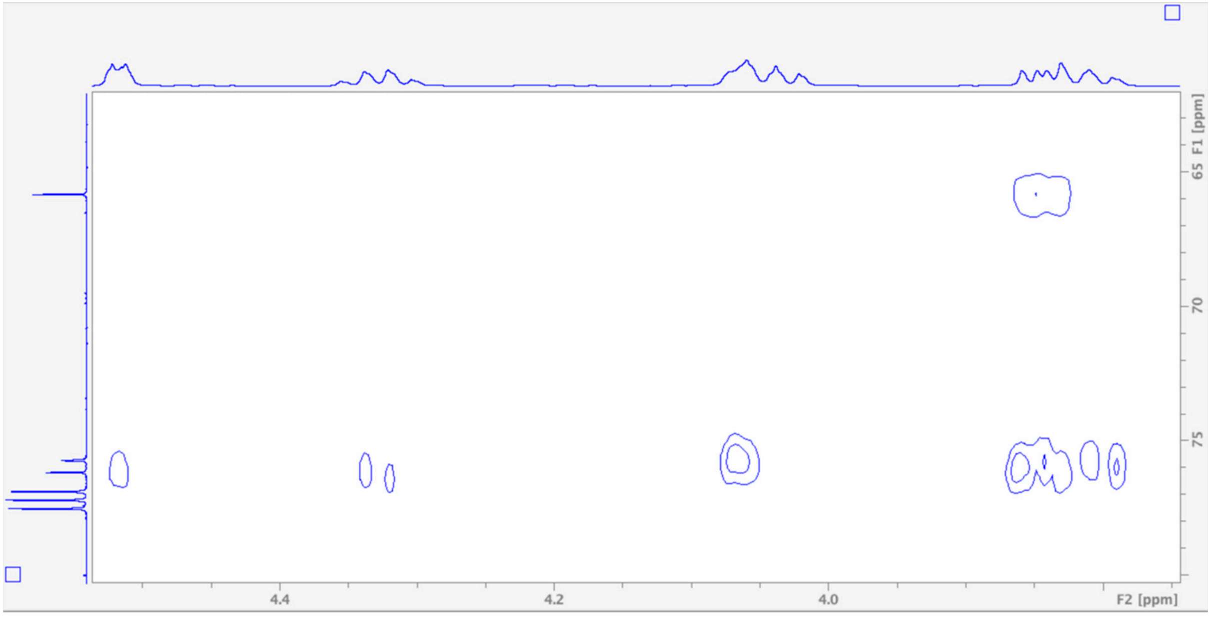
4.4
4.2
4.0
F2 [ppm]
F1 [ppm]
65
70
75

HMQC

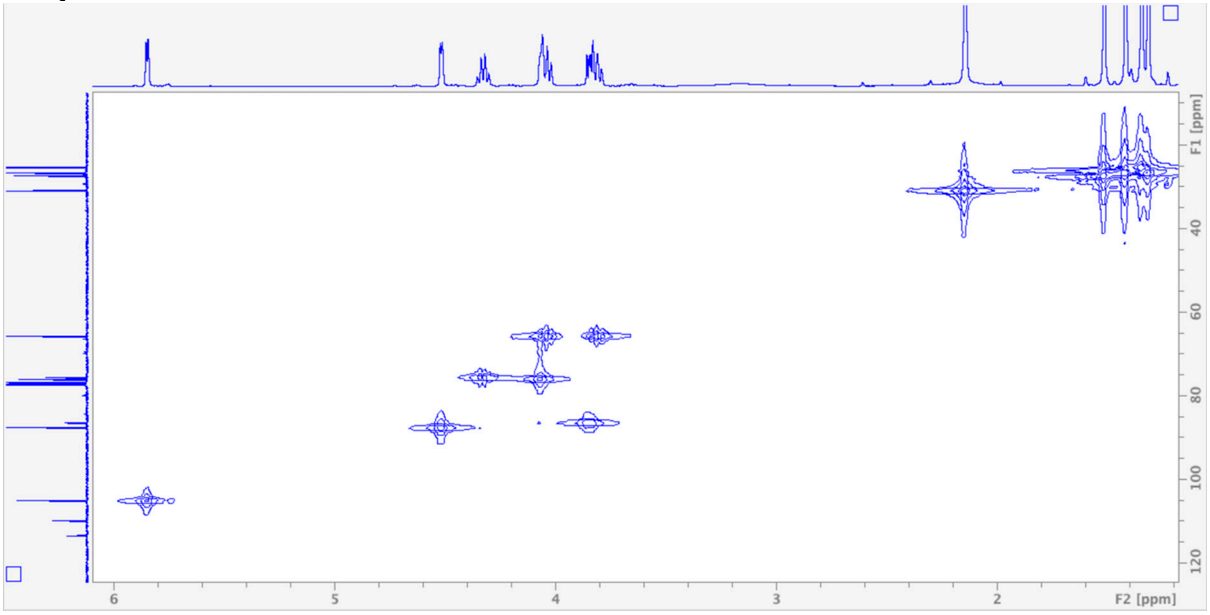

Figure S4. ^{1}H- (400 MHz) and ^{13}C-NMR (100 MHz) spectra of diethanolamine (DEA) in CD_3OD.

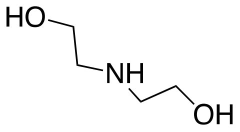

^{1}H-NMR

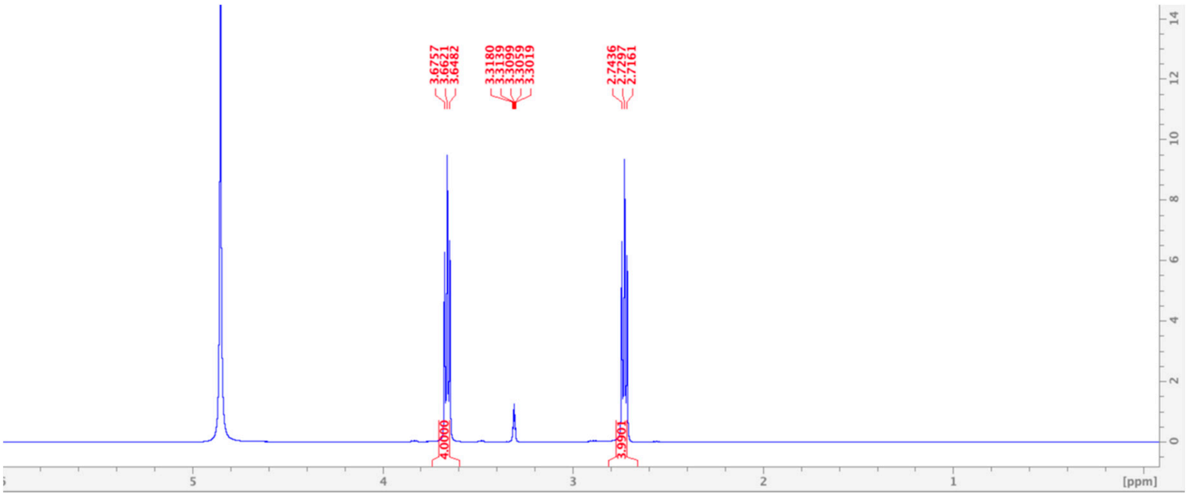

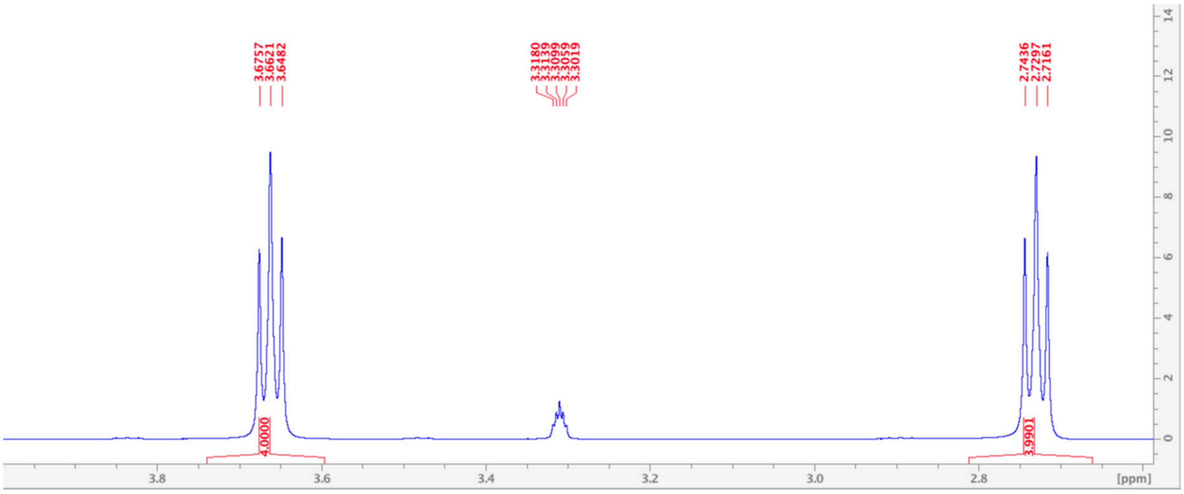

^{13}C-NMR

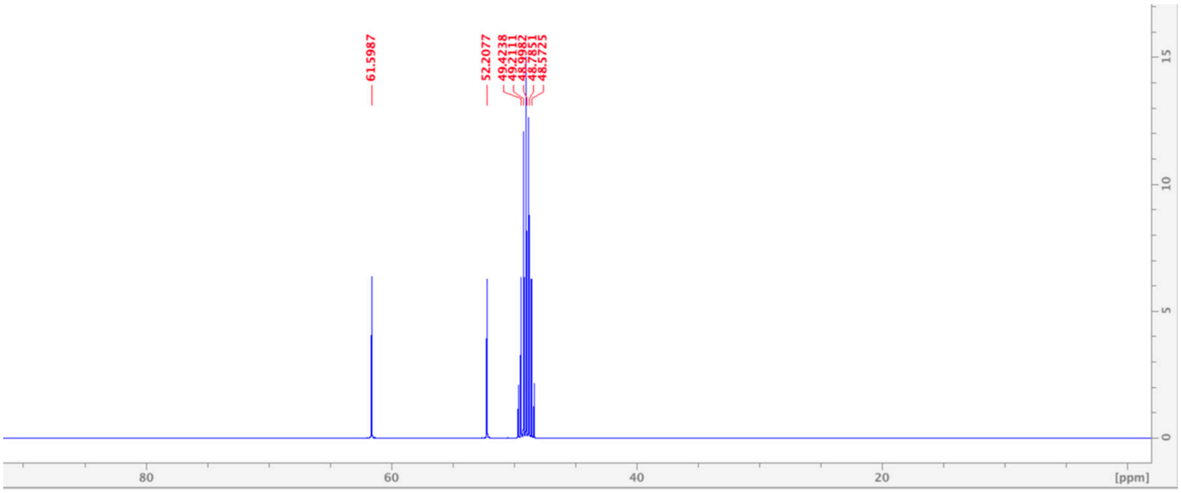
61.5987
52.2077
49.4238
49.2111
48.9982
48.7851
48.5725
80
60
40
20
[ppm]
0
5
10
15

Figure S5. ^{1}H- (400 MHz) and ^{13}C-NMR (100 MHz) spectra of 3-deoxy-1,2;5,6-di-*O*-isopropylidene-α-D-*erythro*-hex-3-enofuranose **3** in $CDCl_3$.

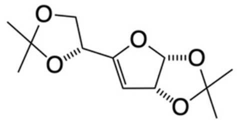

3

^{1}H-NMR

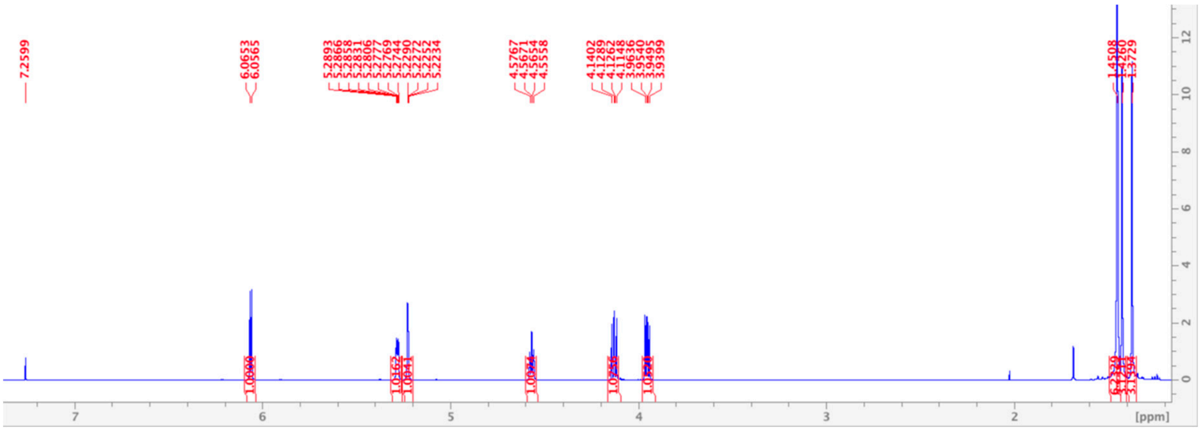

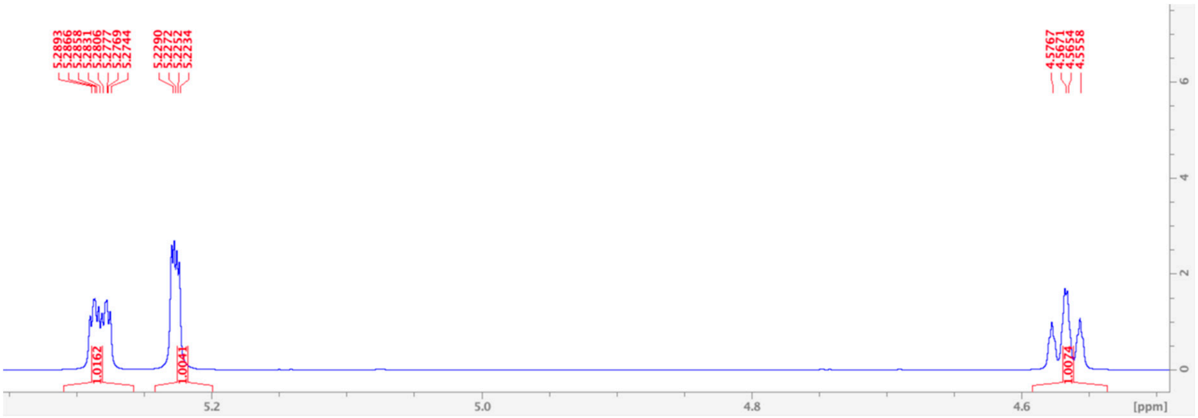

Figure S6. ^{1}H- (400 MHz) and ^{13}C-NMR (100 MHz) spectra of 3-deoxy-1,2;5,6-di-*O*-isopropylidene-α-D-*erythro*-hex-3-enofuranose **3** in acetone-d[6].

^{1}H-NMR

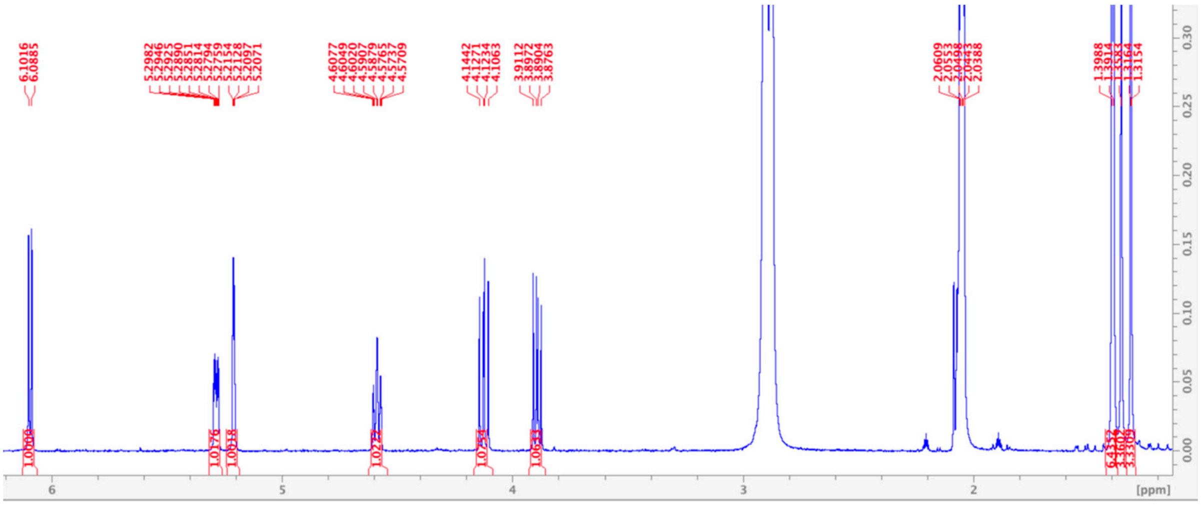

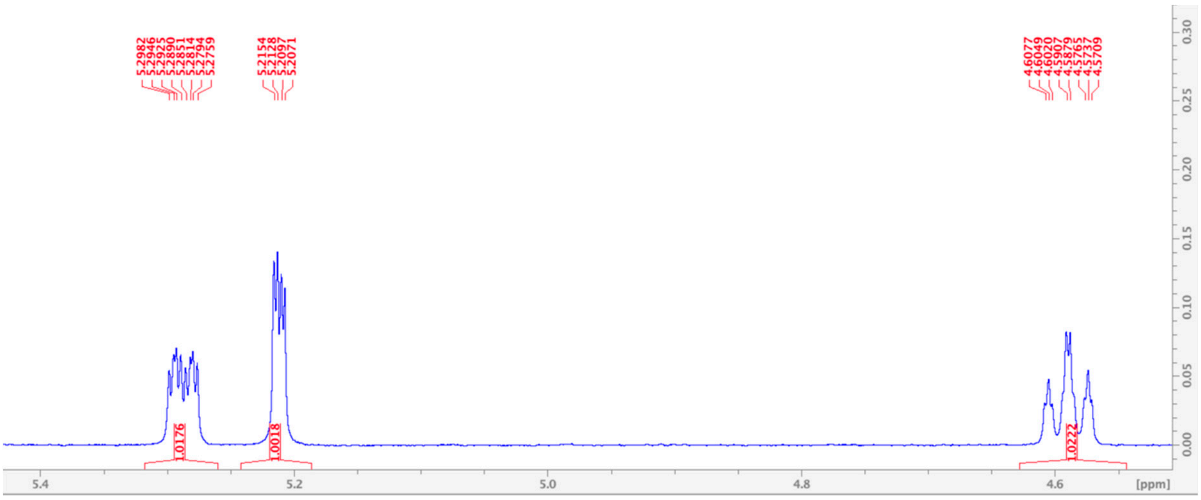

^{13}C-NMR

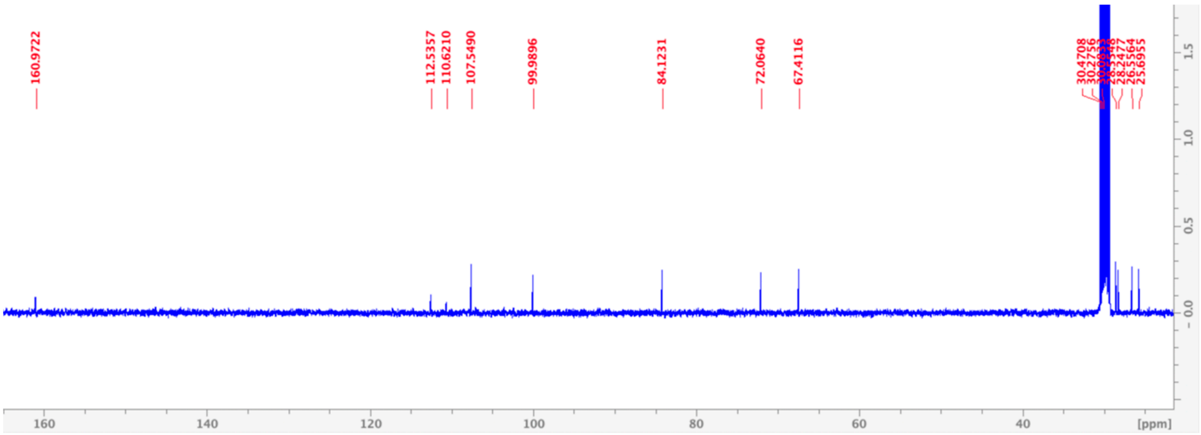

Figure S7. ^{1}H- (400 MHz), ^{13}C-NMR (100 MHz), COSY and HSQC spectra of 3-deoxy-1,2:5,6-di-*O*-isopropylidene-α-D-*xylo*-hexose in $CDCl_3$.

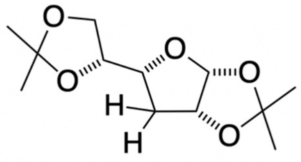

^{1}H-NMR

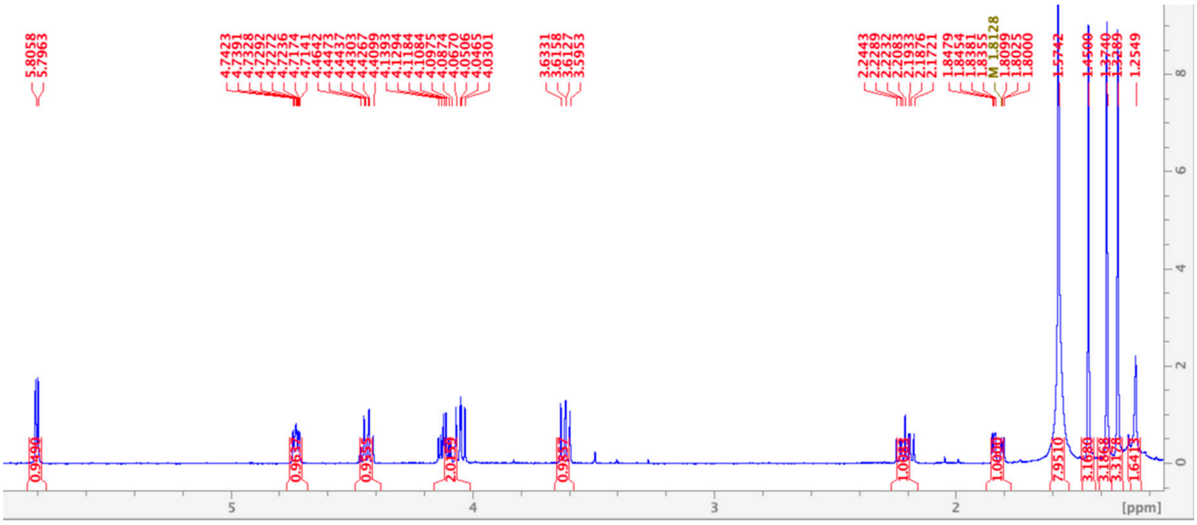

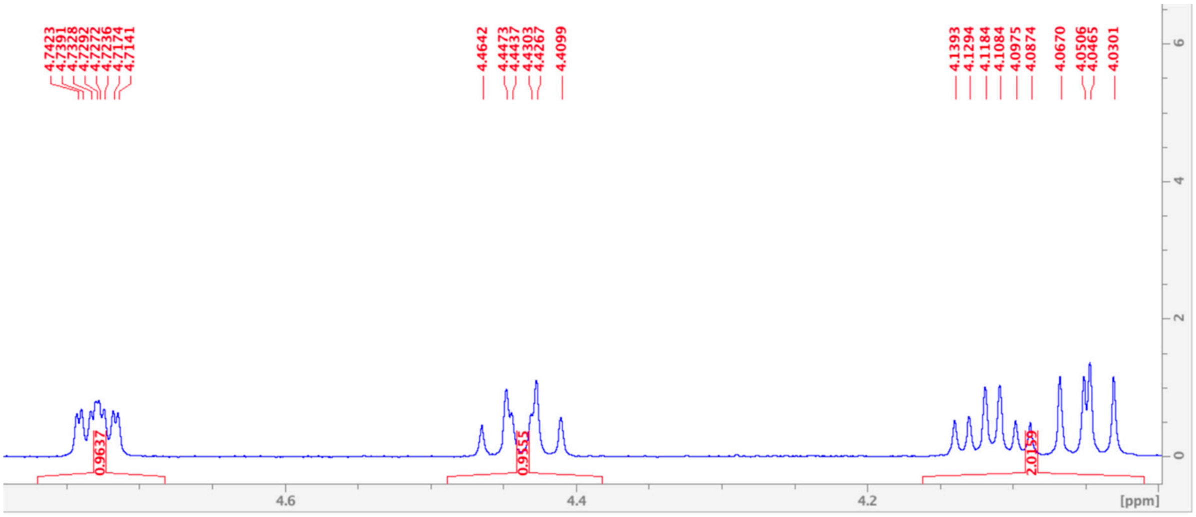

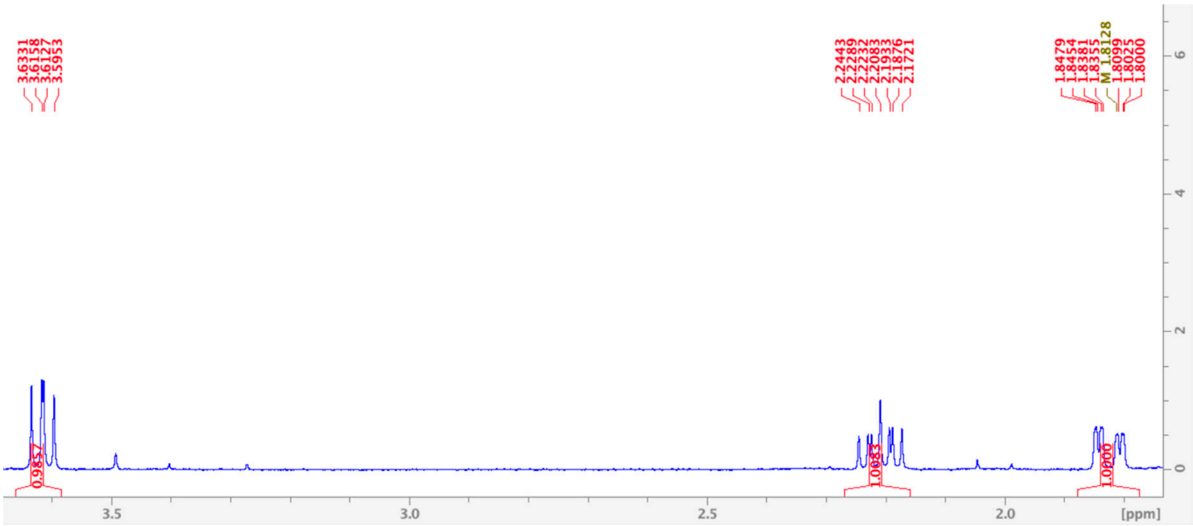
3.6331
3.6158
3.6127
3.5953
2.2443
2.2289
2.2232
2.2083
2.1933
2.1876
2.1721
1.8479
1.8454
1.8381
1.8355
M 1.8128
1.8099
1.8025
1.8000
0.9857
1.0083
1.0000
3.5
3.0
2.5
2.0
[ppm]

^{13}C-NMR

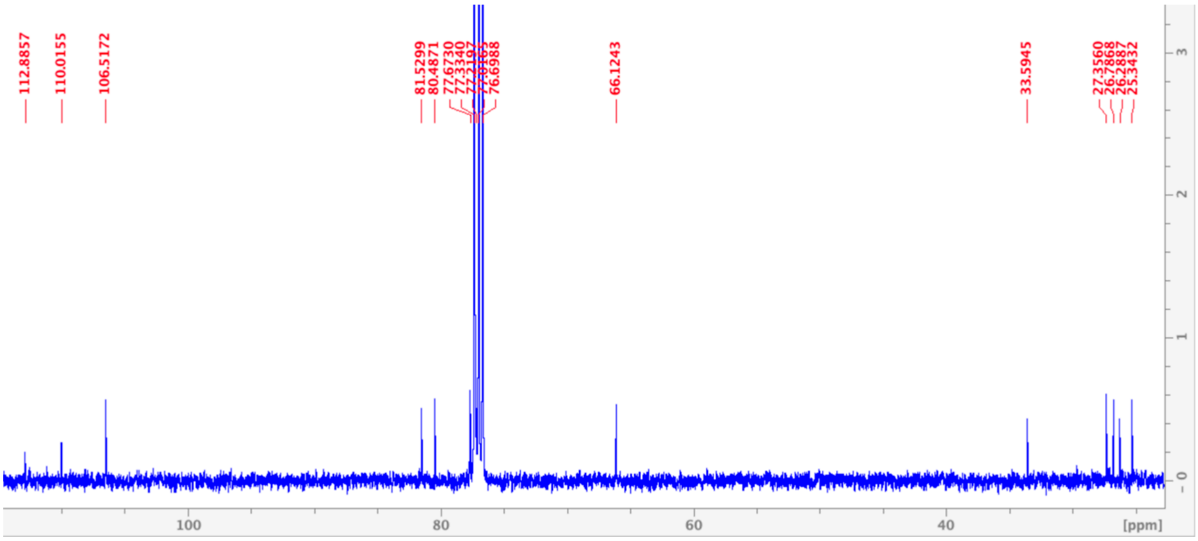
112.8857
110.0155
106.5172
81.5299
80.4871
77.6730
77.3340
66.1243
33.5945
27.3560
26.7868
26.2887
25.3432
100
80
60
40
[ppm]

COSY

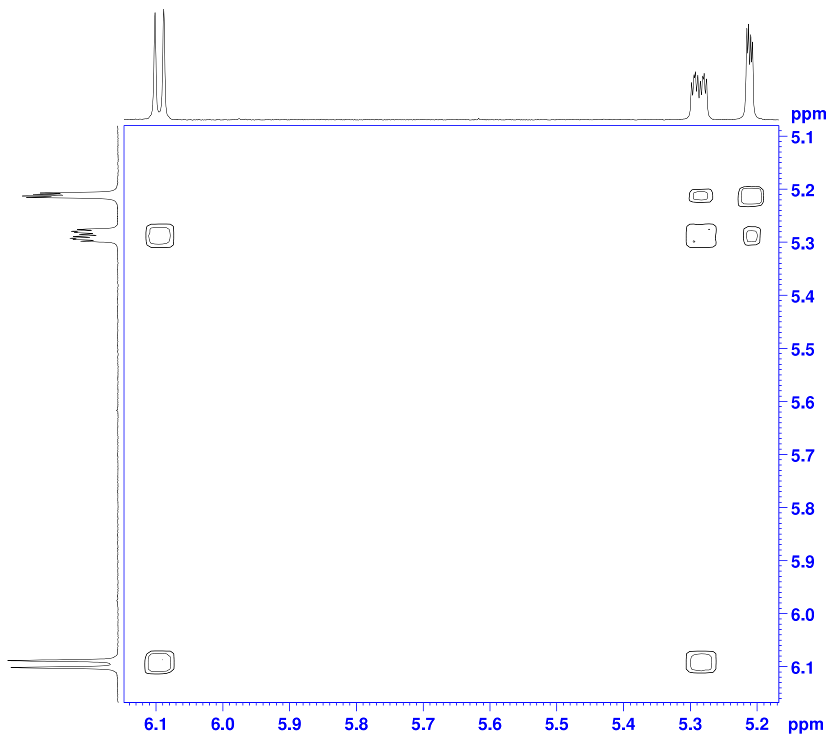
ppm
5.1
5.2
5.3
5.4
5.5
5.6
5.7
5.8
5.9
6.0
6.1
6.1 6.0 5.9 5.8 5.7 5.6 5.5 5.4 5.3 5.2 ppm

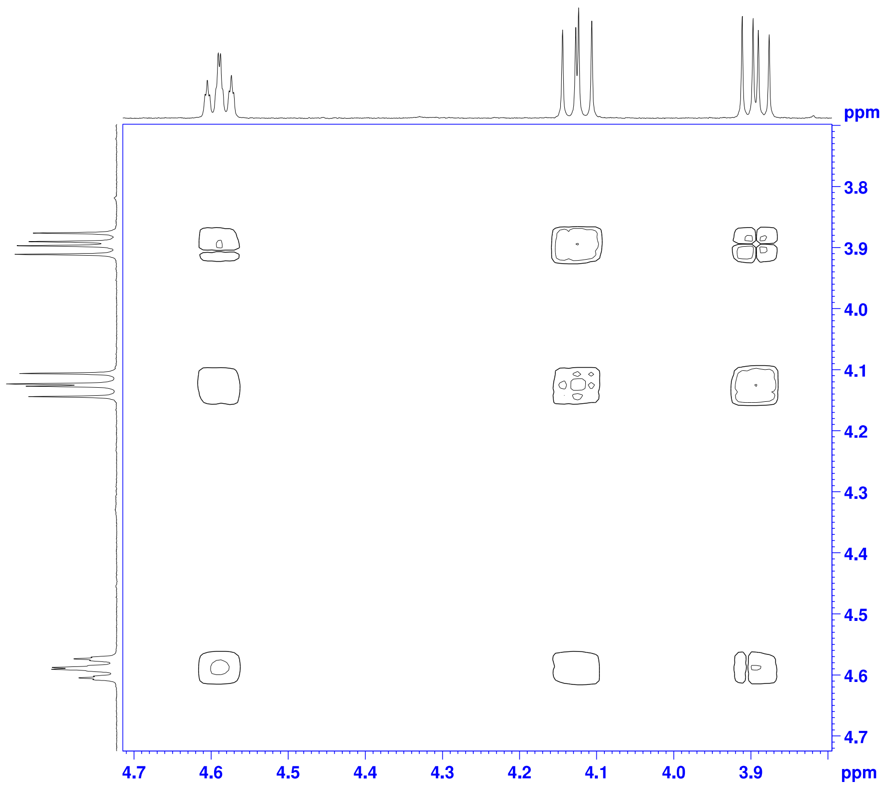
ppm
3.8
3.9
4.0
4.1
4.2
4.3
4.4
4.5
4.6
4.7
4.7 4.6 4.5 4.4 4.3 4.2 4.1 4.0 3.9 ppm

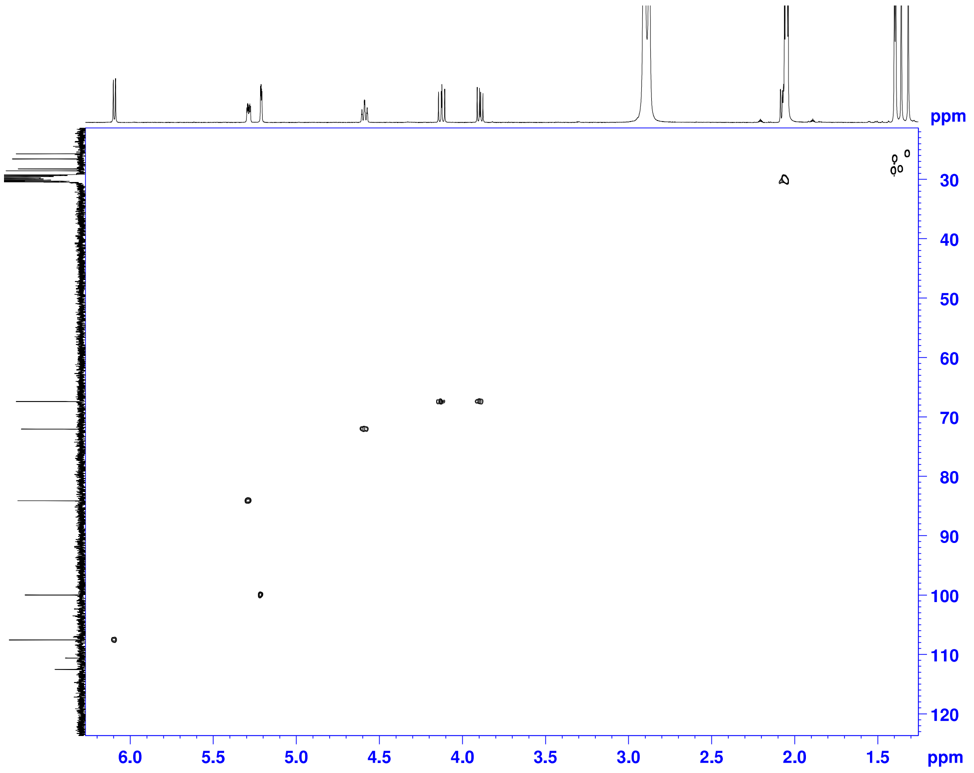

ppm
30
40
50
60
70
80
90
100
110
120
6.0
5.5
5.0
4.5
4.0
3.5
3.0
2.5
2.0
1.5
ppm

Figure S8. ^{1}H- (400 MHz) spectrum of 3-deoxy-3-boronodiethanolamine-1,2:5,6-di-*O*-isopropylidene-α-D-galactofuranose **6** in CD_3OD.

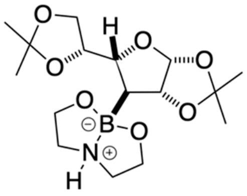

6
D-*Galacto*

^{1}H-NMR

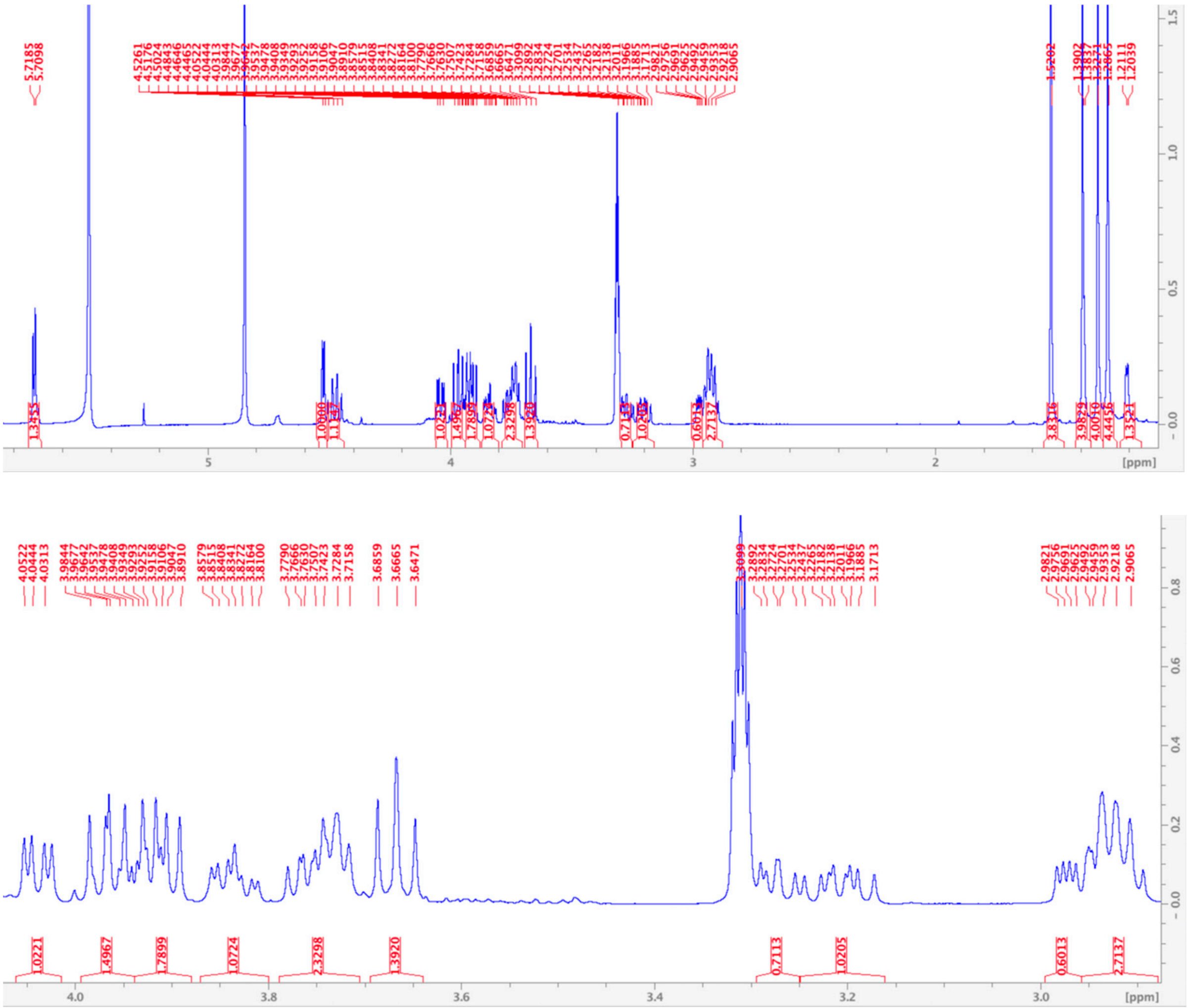

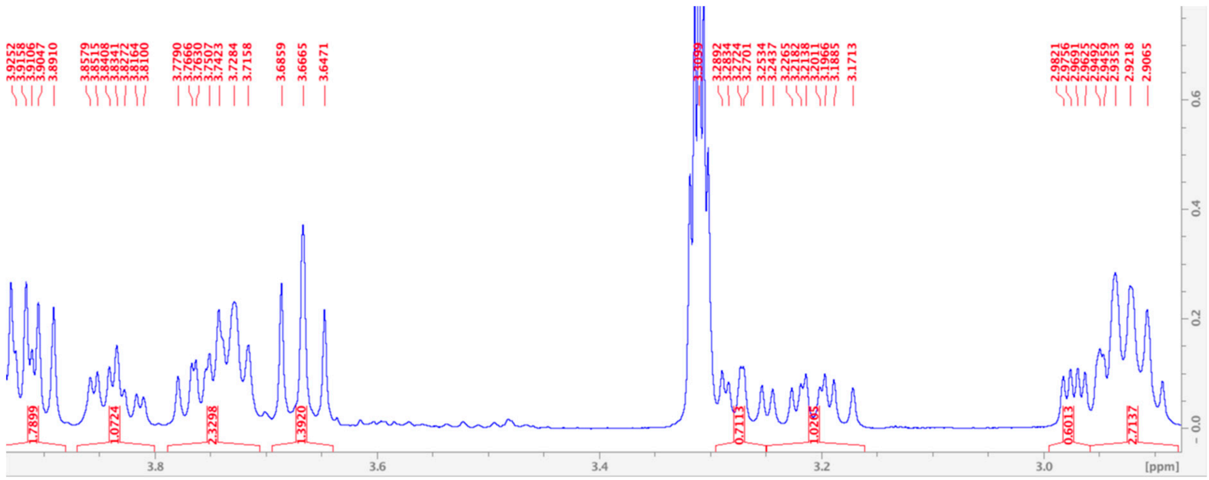
3.9252
3.9158
3.9106
3.9047
3.8910
3.8579
3.8515
3.8408
3.8341
3.8272
3.8164
3.8100
3.7790
3.7666
3.7630
3.7507
3.7423
3.7284
3.7158
3.6859
3.6665
3.6471
3.3099
3.2892
3.2834
3.2724
3.2701
3.2534
3.2437
3.2265
3.2182
3.2138
3.2011
3.1966
3.1885
3.1713
2.9821
2.9756
2.9691
2.9625
2.9492
2.9459
2.9353
2.9218
2.9065
1.7899
1.0724
2.3298
1.3920
0.7113
1.0285
0.6013
2.7137
3.8
3.6
3.4
3.2
3.0
[ppm]
0.0
0.2
0.4
0.6

Figure S9. ^{1}H- (400 MHz), ^{13}C-NMR (100 MHz), DEPT (100 MHz), ^{11}B-NMR (128 MHz), COSY, HSQC and HMBC spectra of 3-deoxy-3-boronodiethanolamine-1,2:5,6-di-*O*-isopropylidene-α-D-galactofuranose **6** in CD_3OD.

^{1}H-NMR

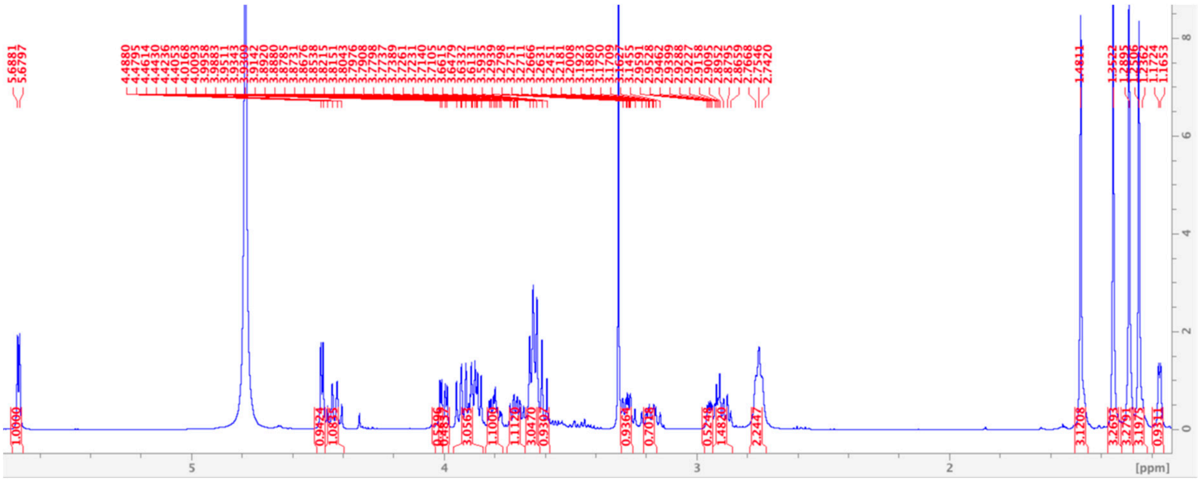

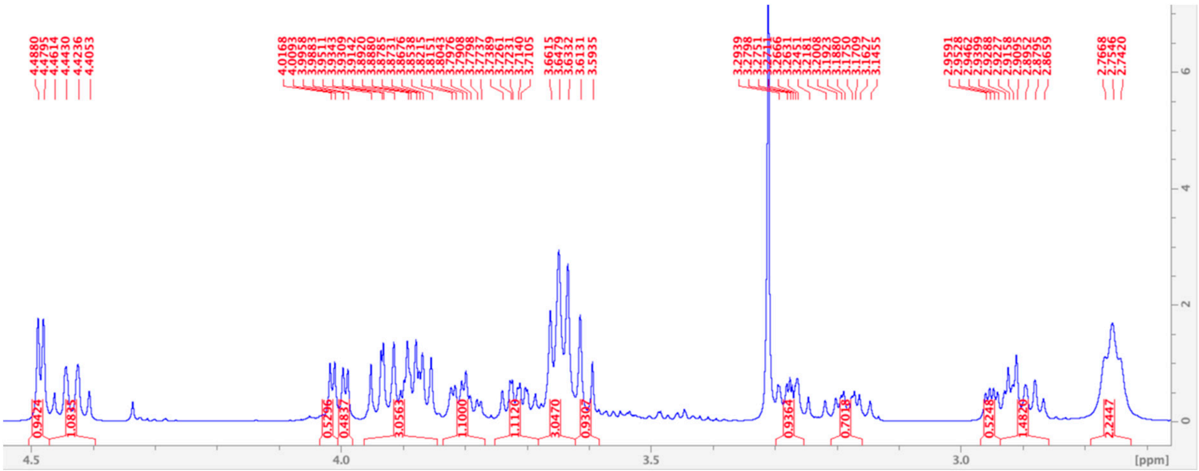

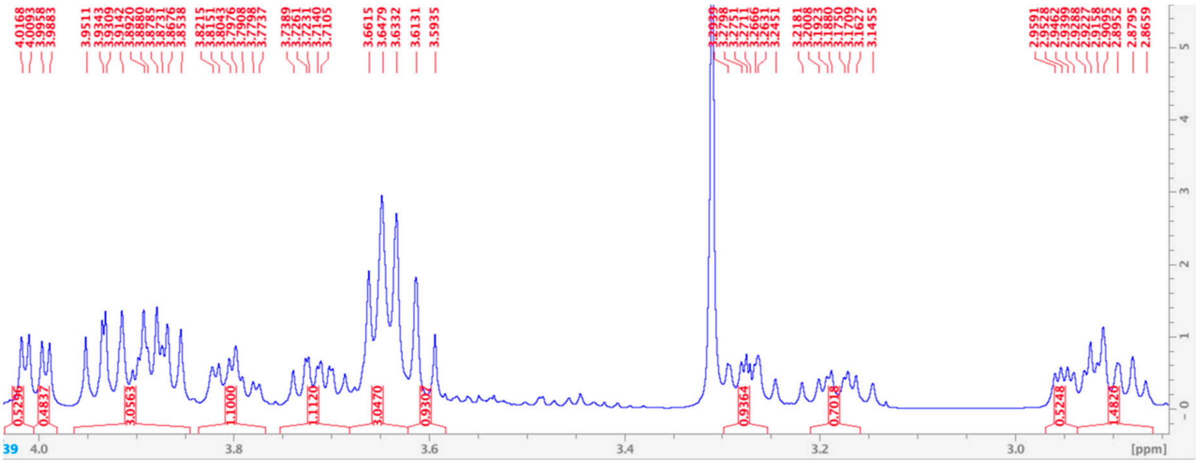

^{13}C-NMR

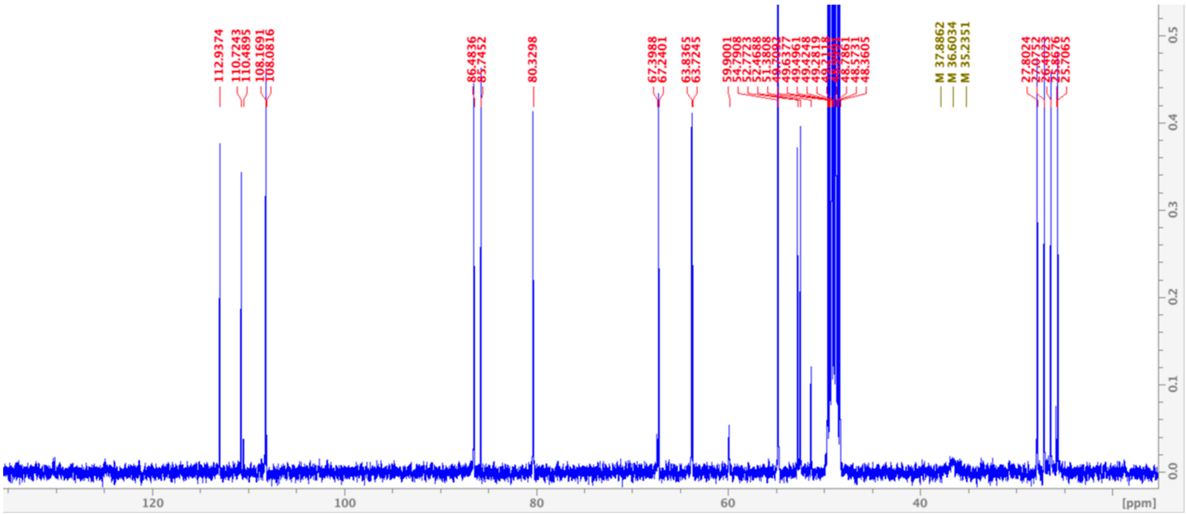

DEPT

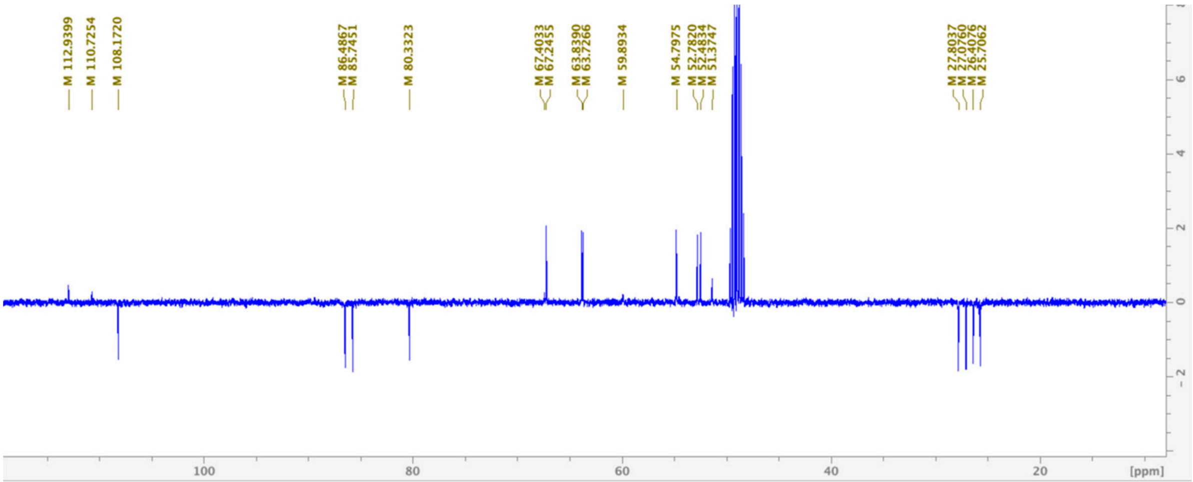

^{11}B-NMR

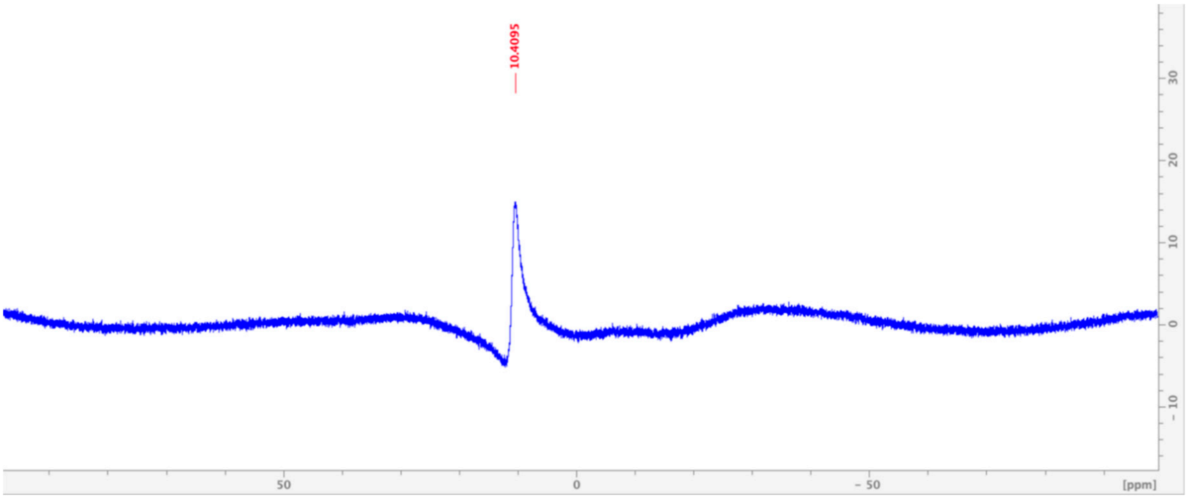

COSY

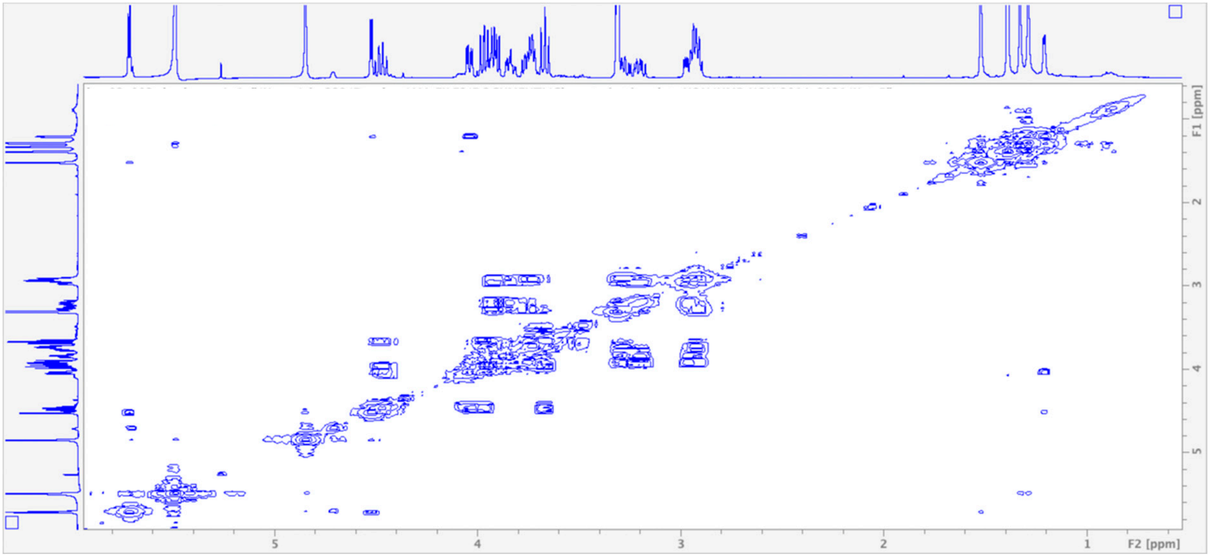

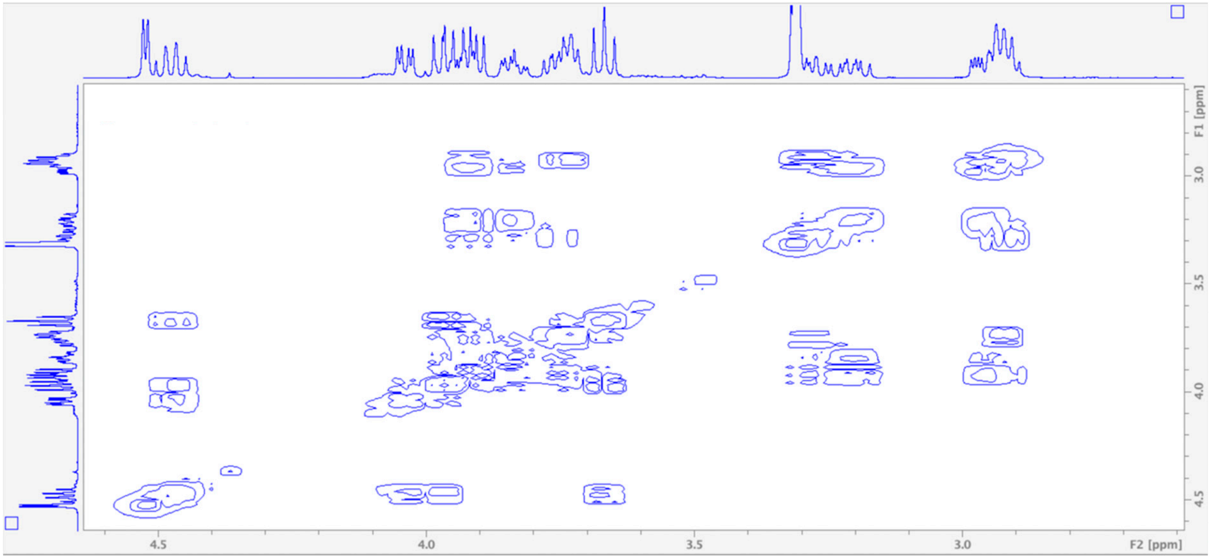

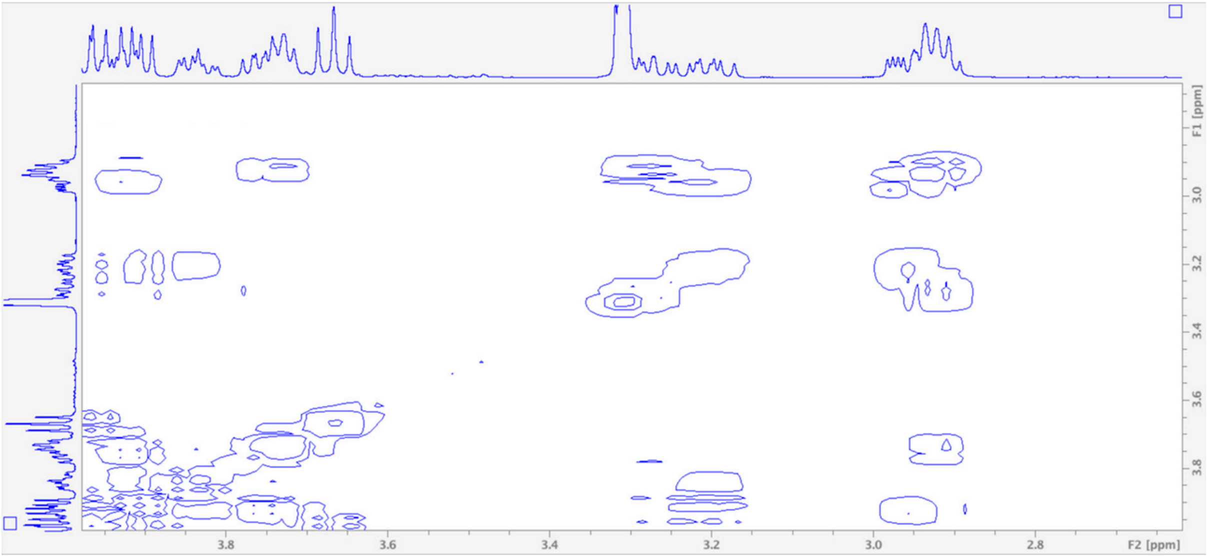

HSQC

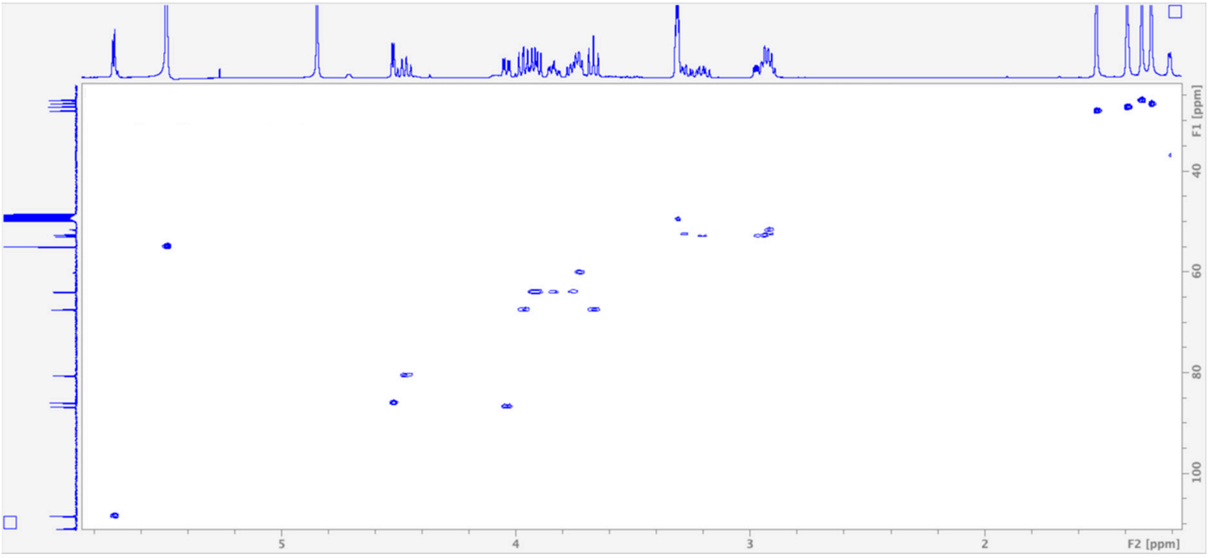

F1 [ppm]
40
60
80
100
5
4
3
2
F2 [ppm]

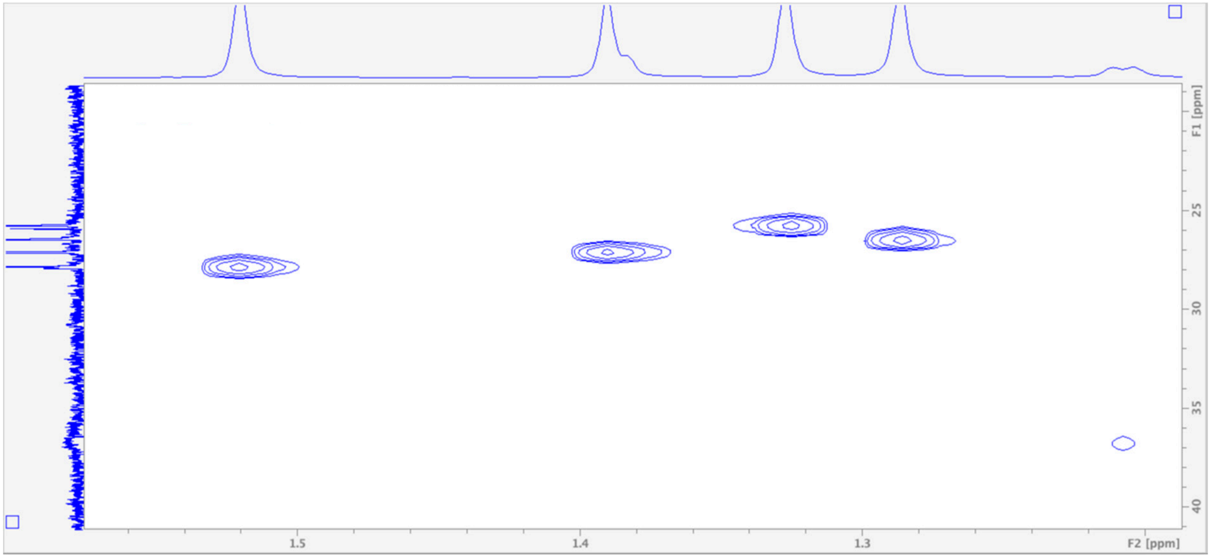

F1 [ppm]
25
30
35
40
1.5
1.4
1.3
F2 [ppm]

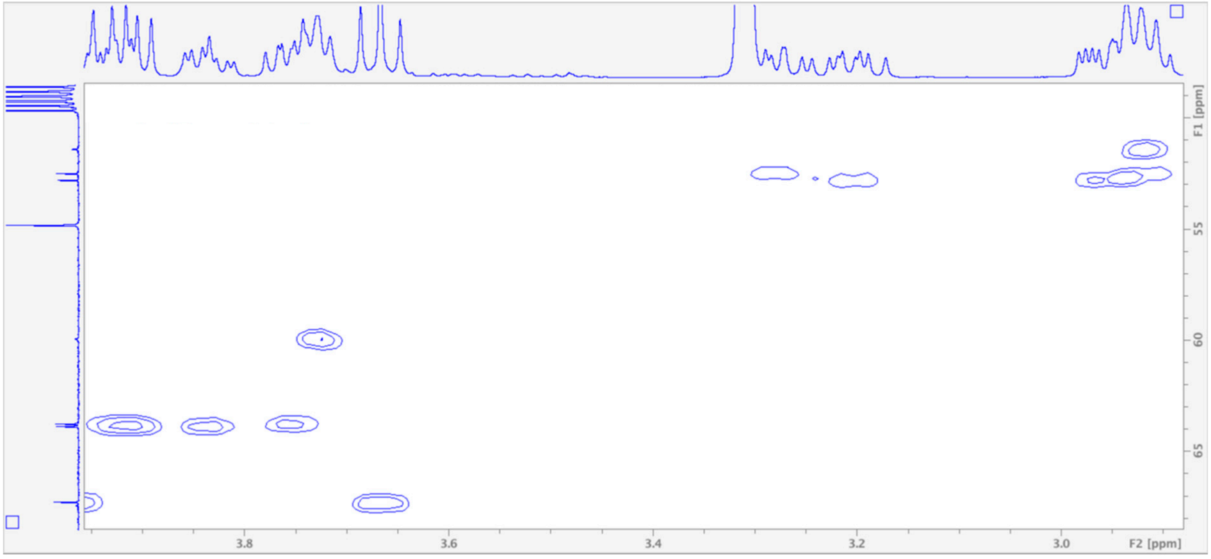

F1 [ppm]
55
60
65
3.8
3.6
3.4
3.2
3.0
F2 [ppm]

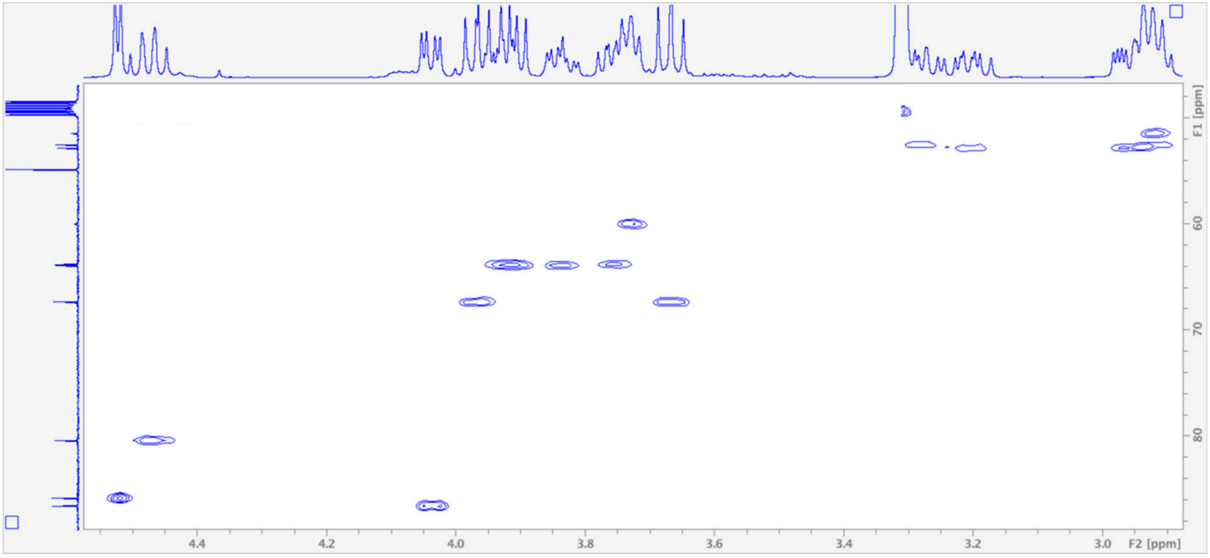
4.4
4.2
4.0
3.8
3.6
3.4
3.2
3.0
F2 [ppm]
F1 [ppm]
60
70
80

HMBC

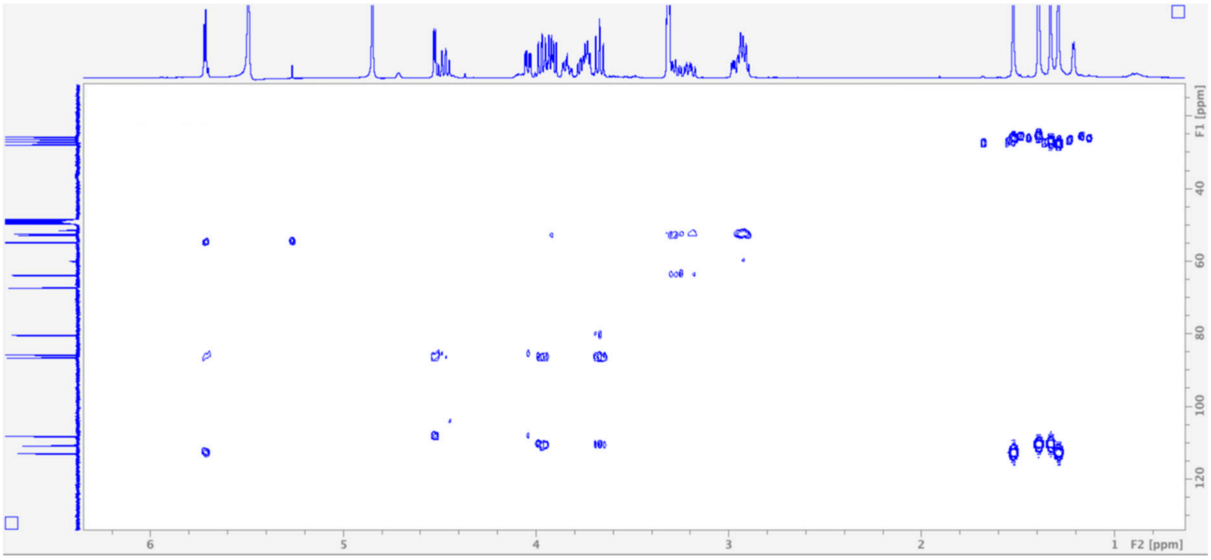
6
5
4
3
2
1
F2 [ppm]
F1 [ppm]
40
60
80
100
120

Figure S10. ^{1}H- (400 MHz), ^{13}C-NMR (100 MHz), ^{11}B-NMR (128 MHz), DEPT (100 MHz), COSY and HSQC spectra of 3-deoxy-3-boronic-D-galactofuranose **7** in D_2O.

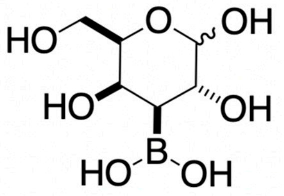

7

D-*Galacto*

^{1}H-NMR

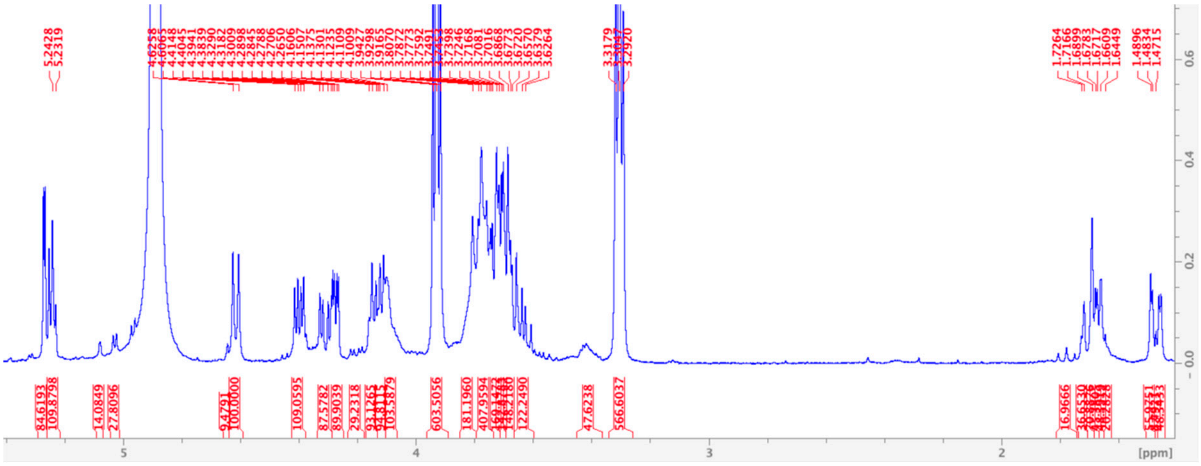

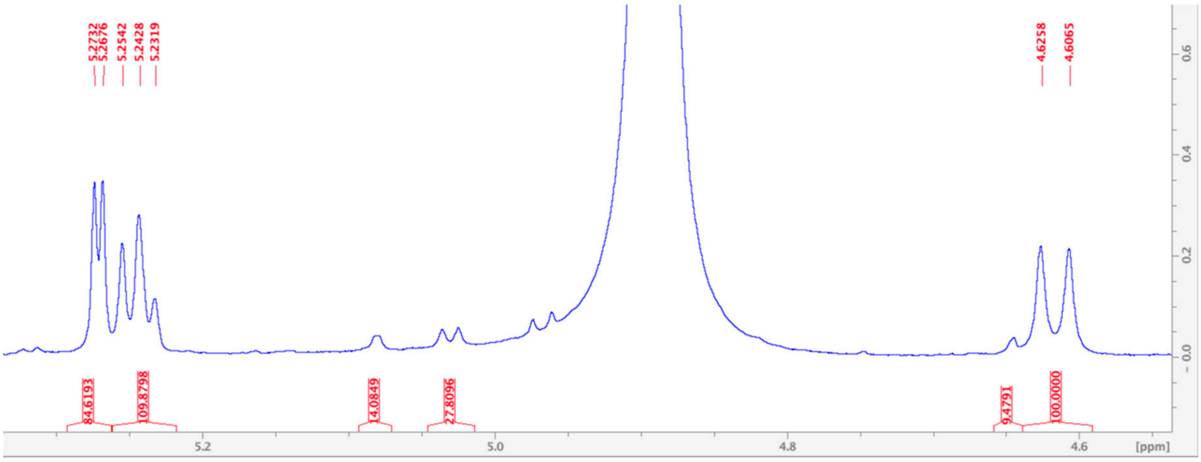

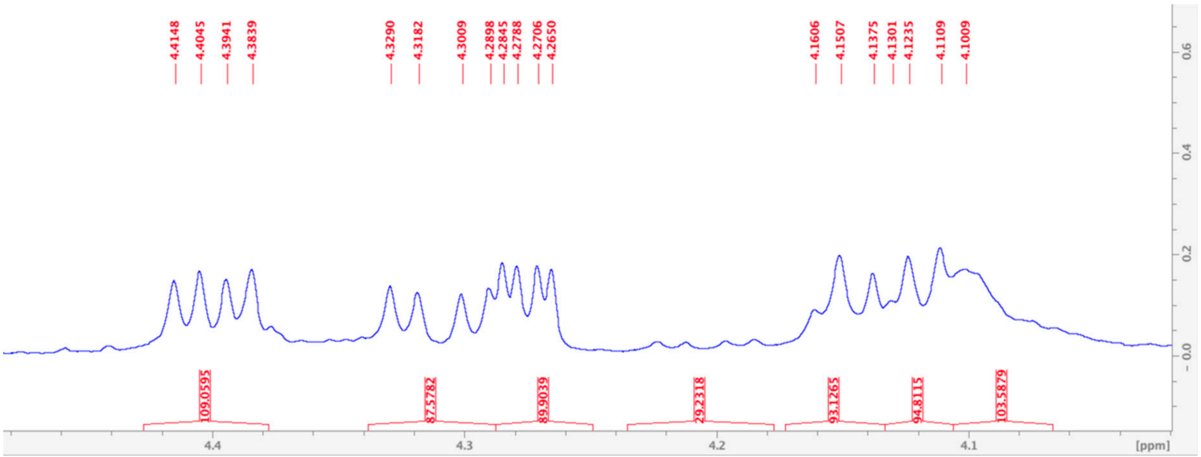
4.4148
4.4045
4.3941
4.3839
4.3290
4.3182
4.3009
4.2898
4.2845
4.2788
4.2706
4.2650
4.1606
4.1507
4.1375
4.1301
4.1235
4.1109
4.1009
109.0595
87.5782
89.9039
29.2318
93.1265
94.8115
103.5879
4.4
4.3
4.2
4.1
[ppm]
0.6
0.4
0.2
0.0

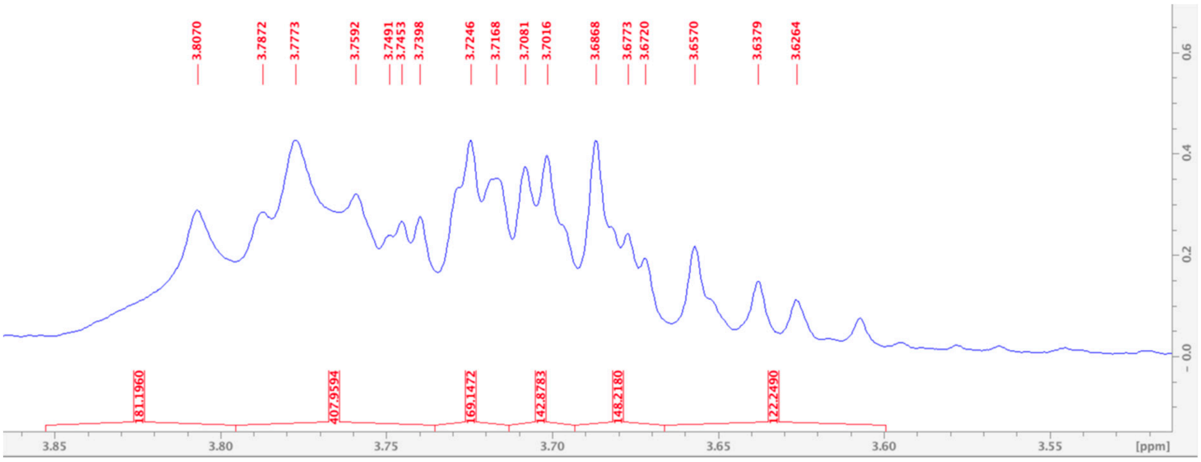
3.8070
3.7872
3.7773
3.7592
3.7491
3.7453
3.7398
3.7246
3.7168
3.7081
3.7016
3.6868
3.6773
3.6720
3.6570
3.6379
3.6264
181.1960
407.9594
169.1472
42.8783
48.2180
122.2490
3.85
3.80
3.75
3.70
3.65
3.60
3.55
[ppm]
0.6
0.4
0.2
0.0

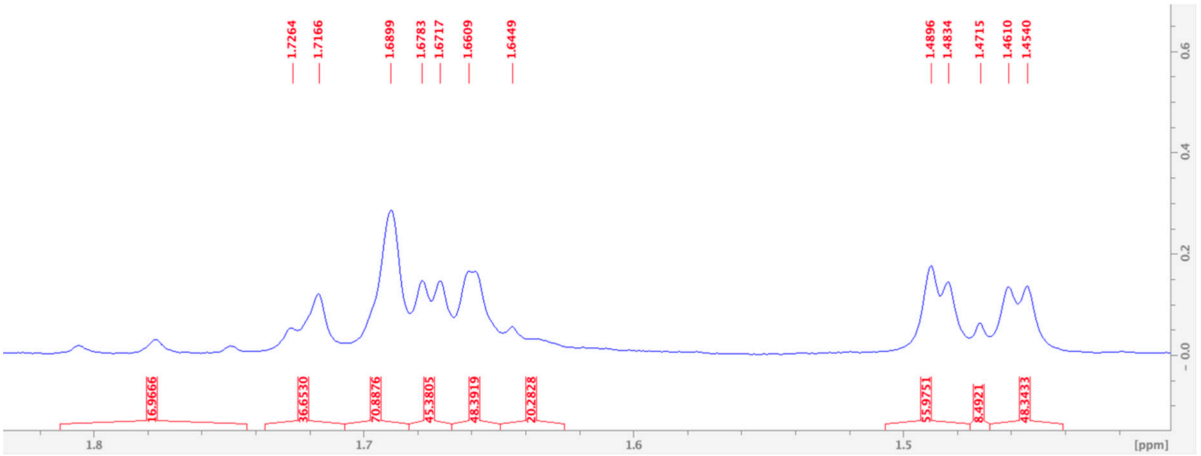
1.7264
1.7166
1.6899
1.6783
1.6717
1.6609
1.6449
1.4896
1.4834
1.4715
1.4610
1.4540
16.9666
36.6530
70.8876
45.3805
48.3919
20.2828
55.9751
8.4921
48.3433
1.8
1.7
1.6
1.5
[ppm]
0.6
0.4
0.2
0.0

^{13}C-NMR

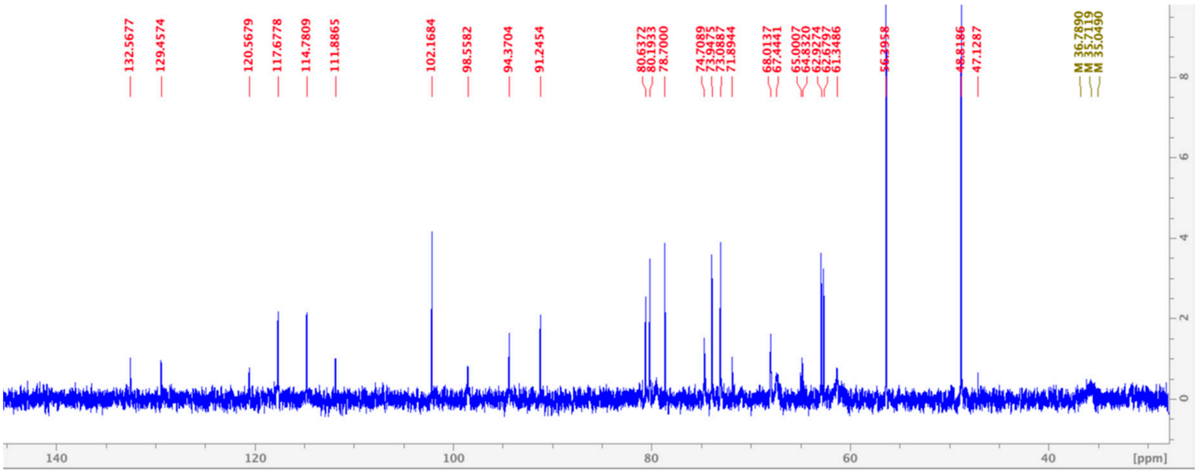
132.5677
129.4574
120.5679
117.6778
114.7809
111.8865
102.1684
98.5582
94.3704
91.2454
80.6372
80.1933
78.7000
74.7089
73.9475
73.0887
71.8944
68.0137
67.4441
65.0007
64.8320
62.9274
61.3486
56.3958
48.8186
47.1287
M 36.7890
M 35.7119
M 35.0490
140
120
100
80
60
40
[ppm]

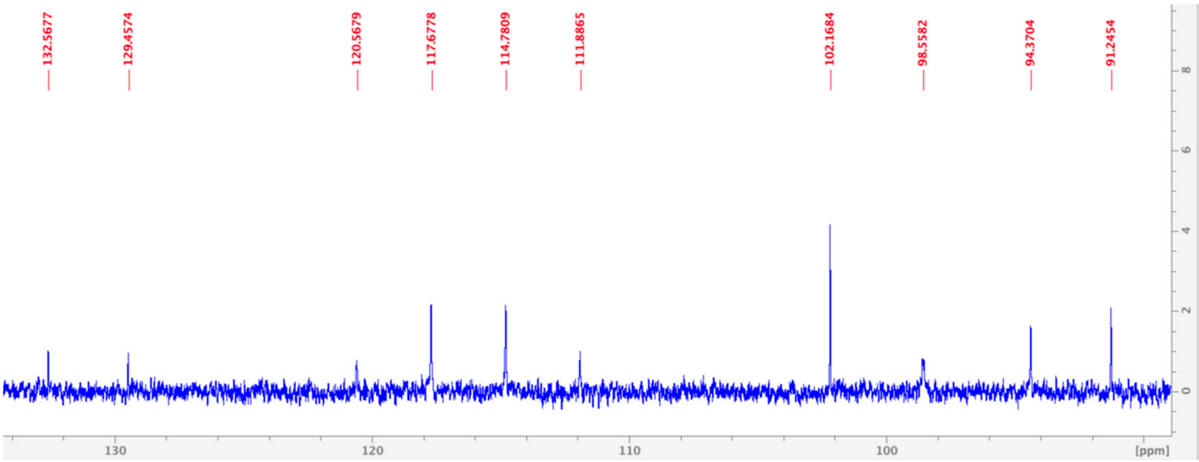
132.5677
129.4574
120.5679
117.6778
114.7809
111.8865
102.1684
98.5582
94.3704
91.2454
130
120
110
100
[ppm]

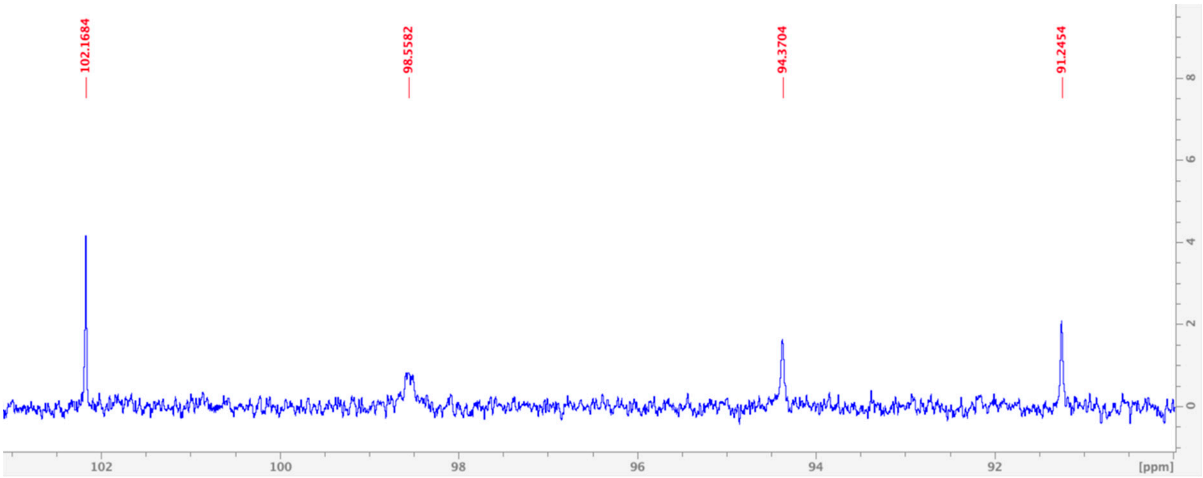
102.1684
98.5582
94.3704
91.2454
102
100
98
96
94
92
[ppm]

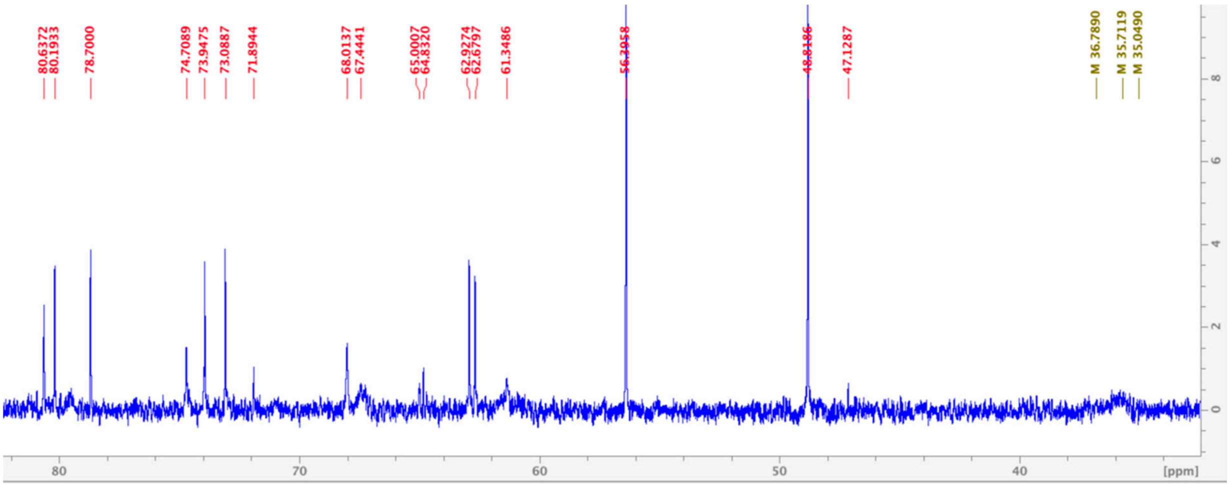
80.6372
80.1933
78.7000
74.7089
73.9475
73.0887
71.8944
68.0137
67.4441
65.0007
64.8320
62.9274
62.6797
61.3486
56.3958
48.8186
47.1287
M 36.7890
M 35.7119
M 35.0490
[ppm]

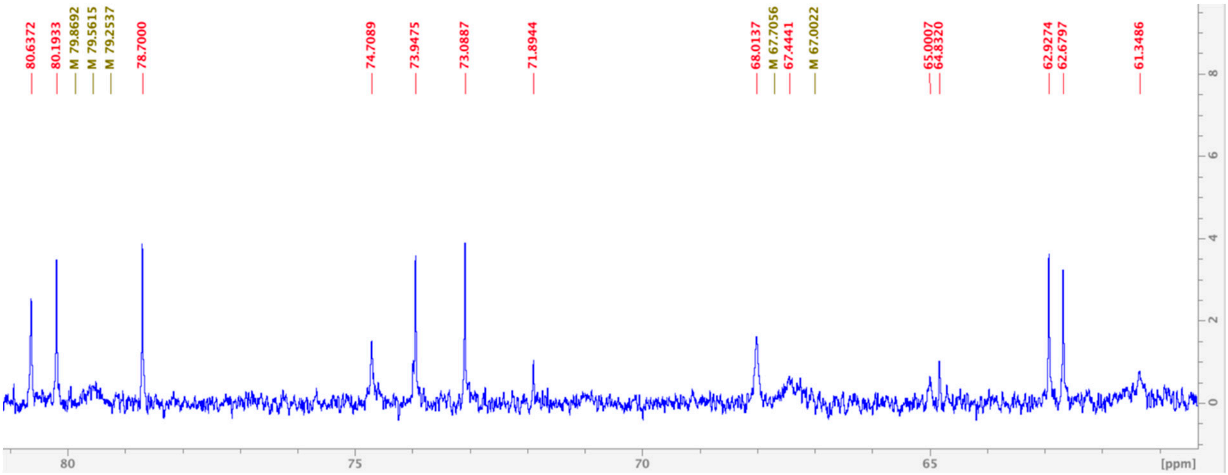
80.6372
80.1933
M 79.8692
M 79.5615
M 79.2537
78.7000
74.7089
73.9475
73.0887
71.8944
68.0137
M 67.7056
67.4441
M 67.0022
65.0007
64.8320
62.9274
62.6797
61.3486
[ppm]

^{11}B-NMR

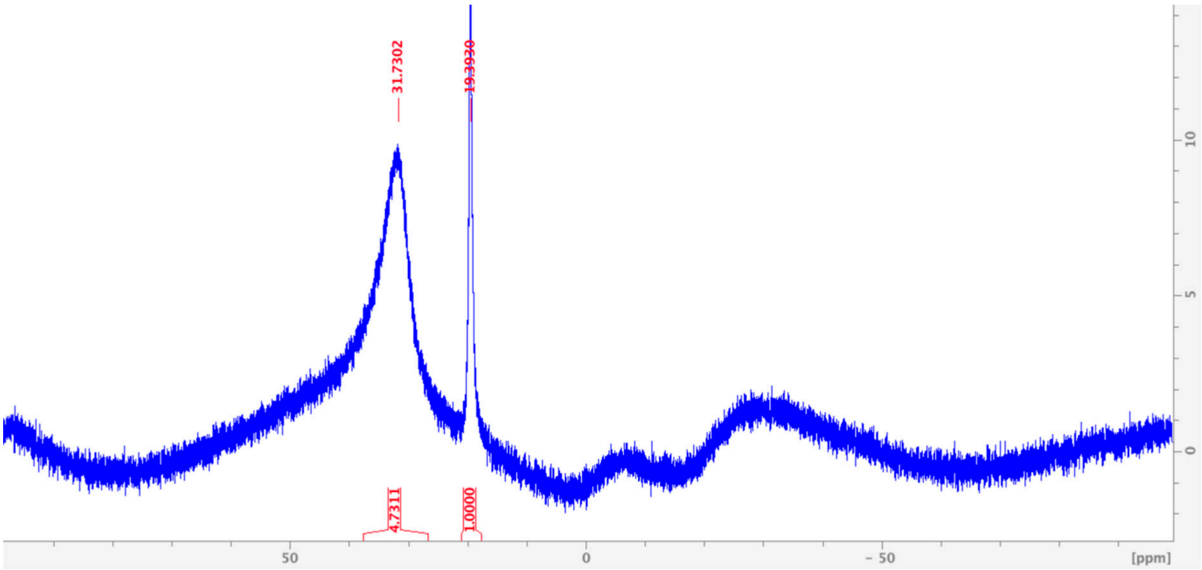
31.7302
19.3930
4.7311
1.0000
[ppm]

DEPT

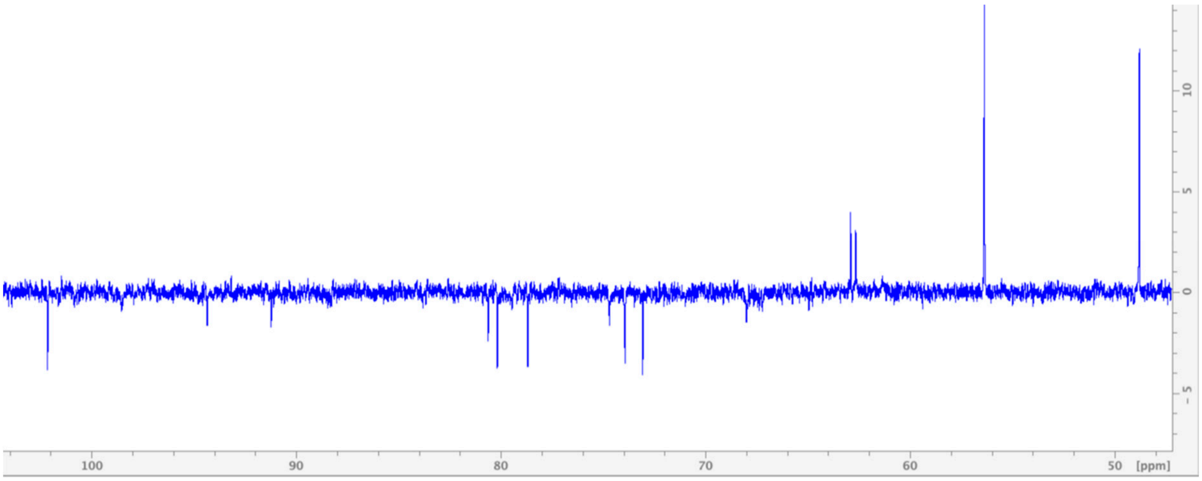

100
90
80
70
60
50
[ppm]
10
5
0
-5

COSY

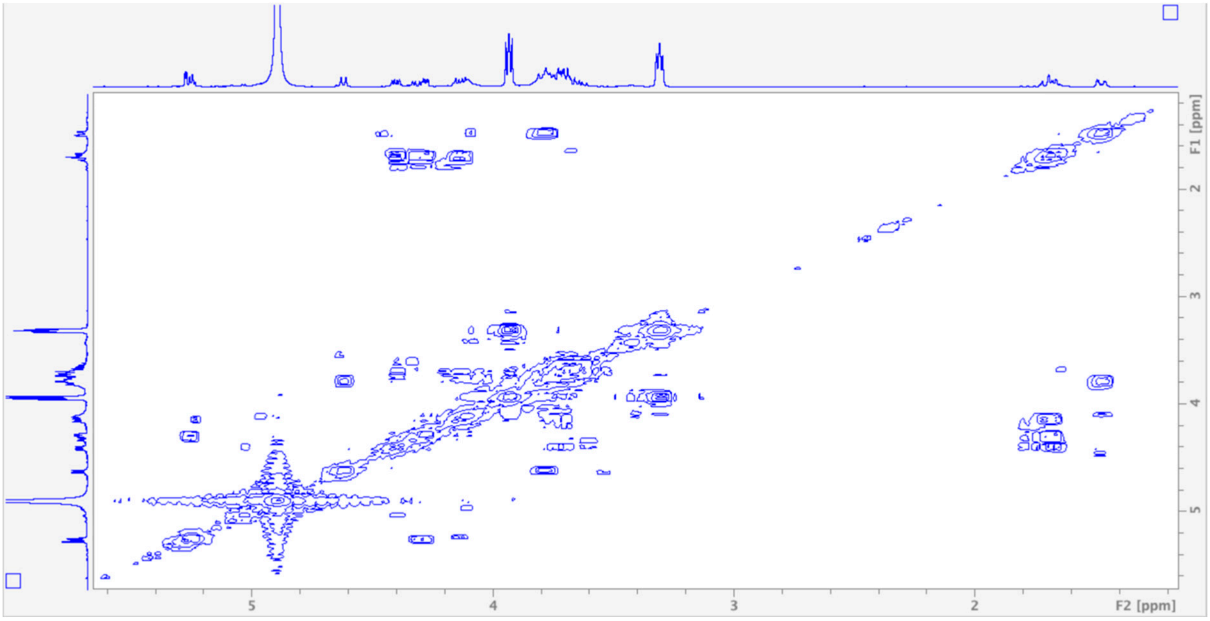

F1 [ppm]
2
3
4
5
5
4
3
2
F2 [ppm]

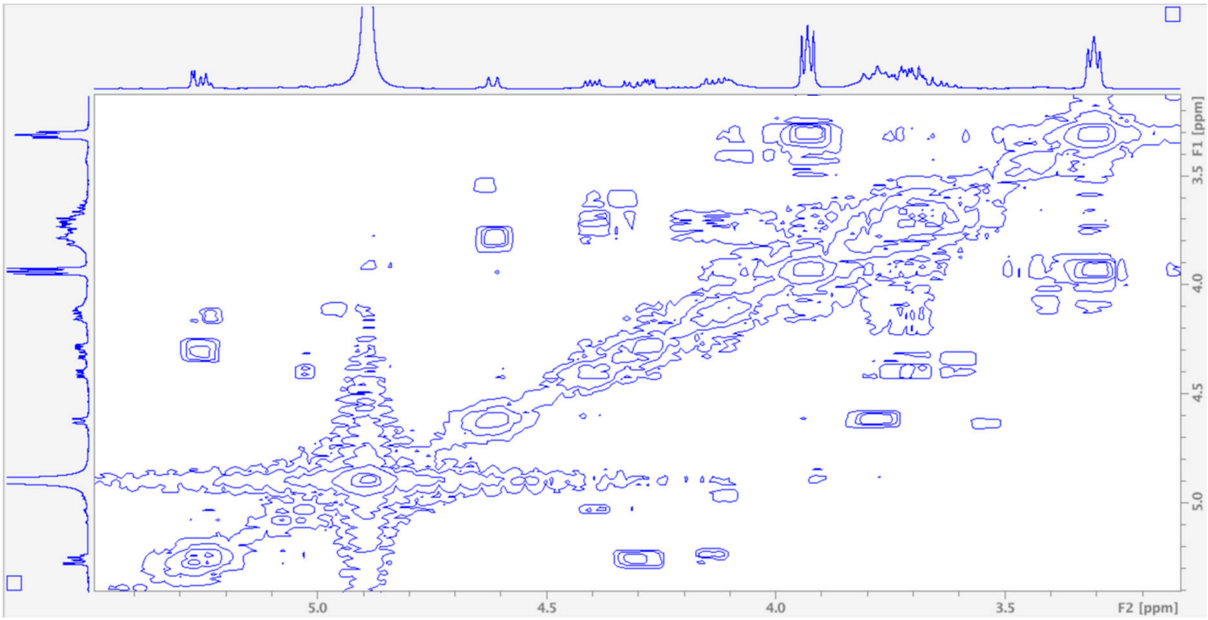

F1 [ppm]
3.5
4.0
4.5
5.0
5.0
4.5
4.0
3.5
F2 [ppm]

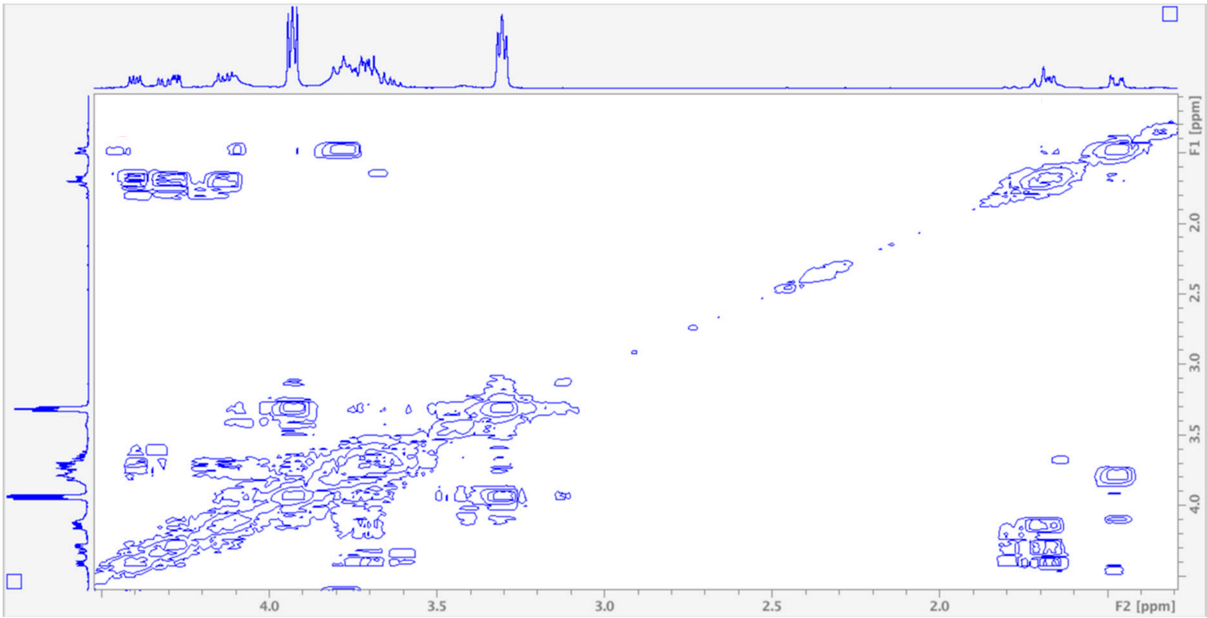

HSQC

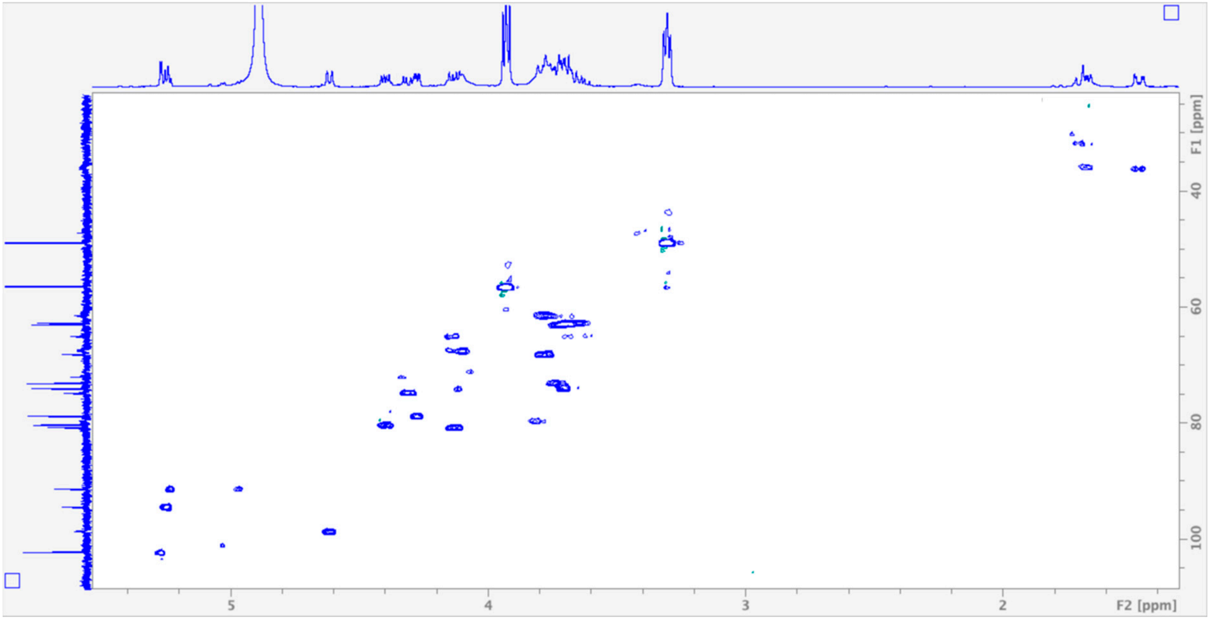

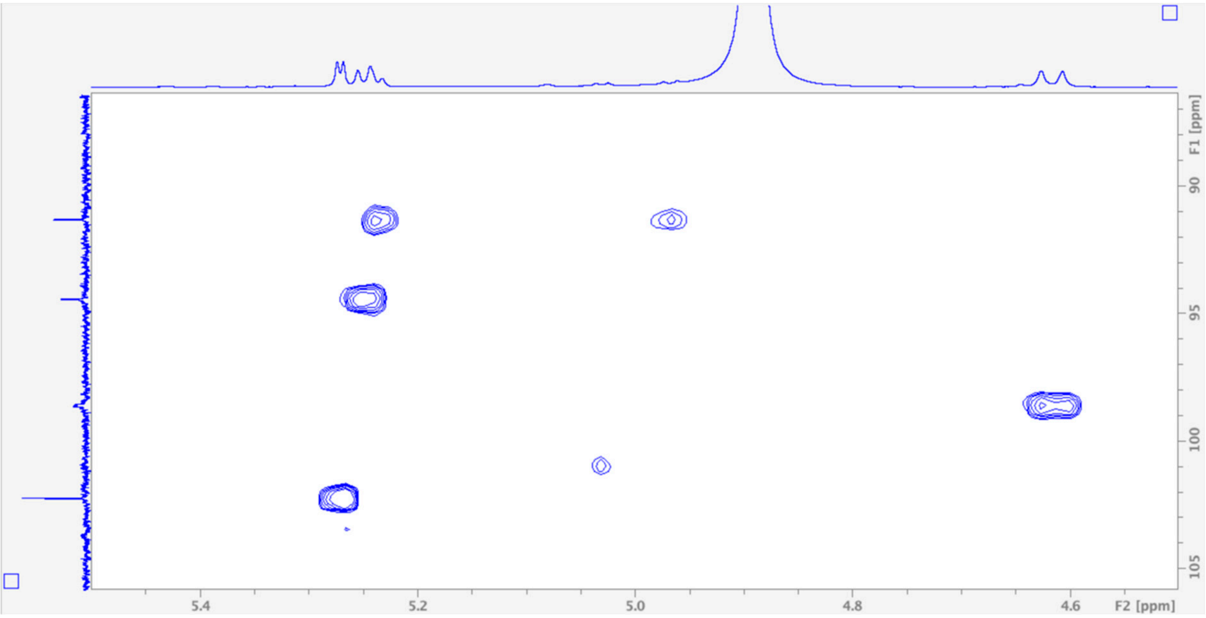

F1 [ppm]
90
95
100
105
5.4
5.2
5.0
4.8
4.6
F2 [ppm]

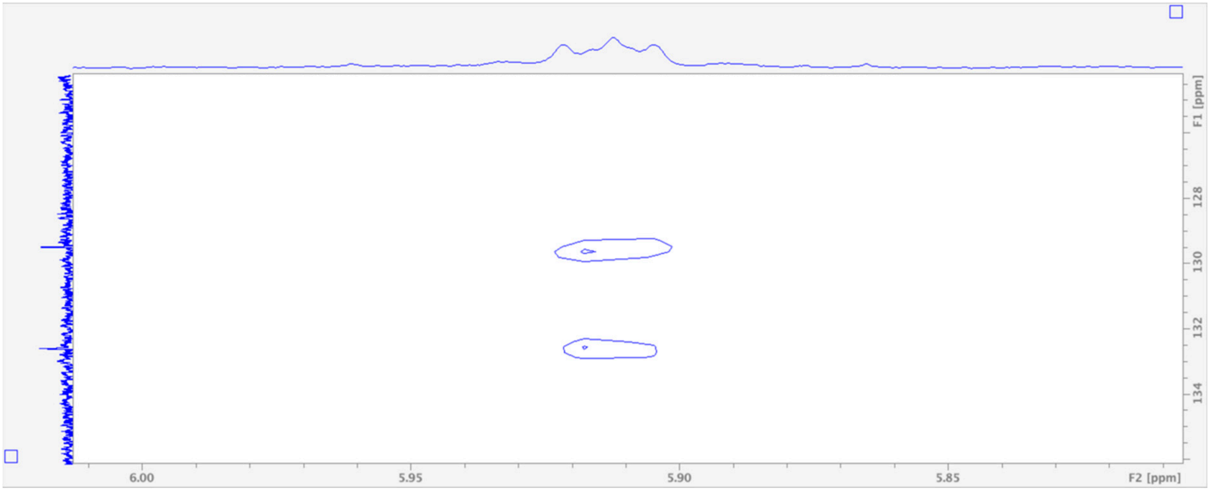

F1 [ppm]
128
130
132
134
6.00
5.95
5.90
5.85
F2 [ppm]

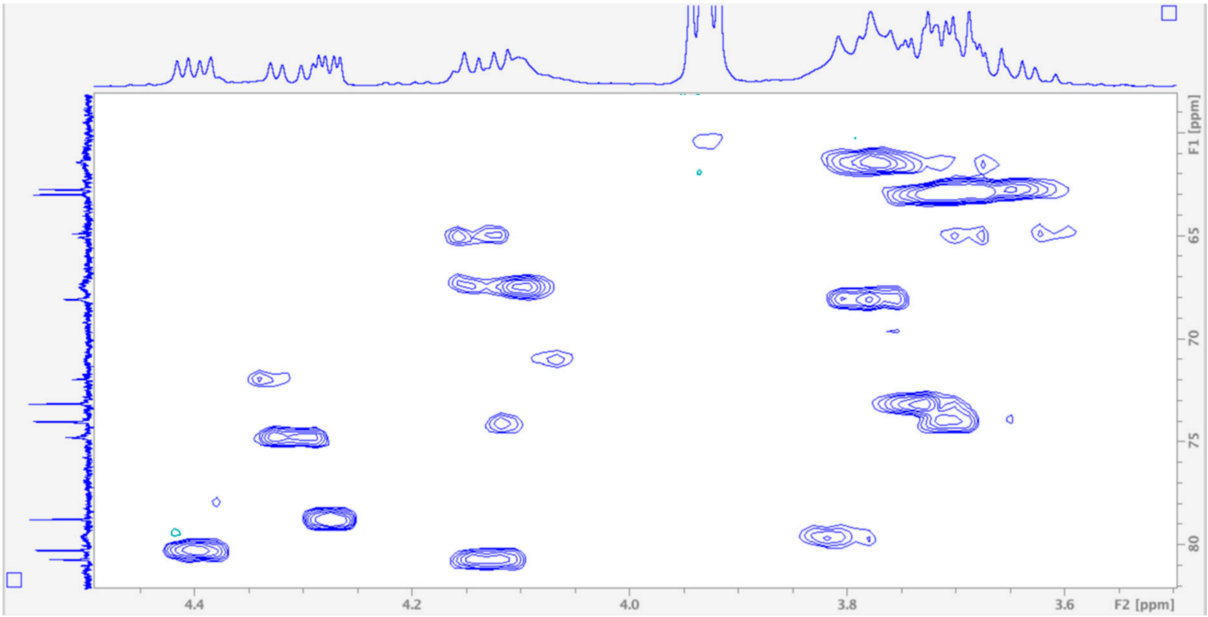

F1 [ppm]
65
70
75
80
4.4
4.2
4.0
3.8
3.6
F2 [ppm]

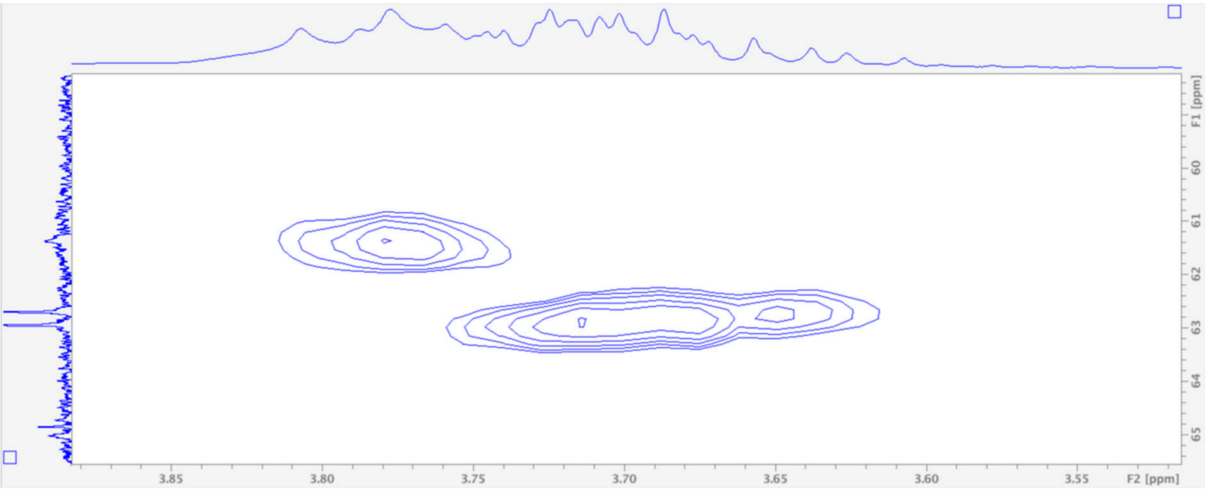

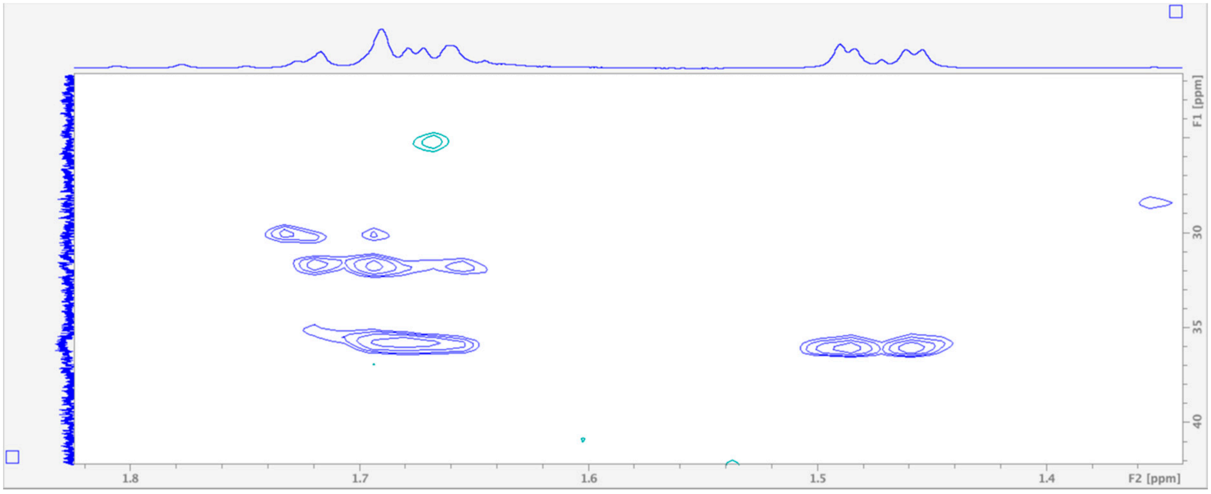

References

24. Rochepeau-Jobron, L.; Jacquinet, J.-C. Diastereoselective hydroboration of substituted exo-glucals revisited. A convenient route for the preparation of L-iduronic acid derivatives *Carbohydr. Res.* **1997**, *303*, 395–406.

25. Paulsen, H.; Behre, H. Umwandlung von 1,2:5,6-Di-O-isopropyliden-3-desoxy-α-D-glucose-3-en in 1,2:5,6-Di-O-isopropyliden-a-d-galactofuranose durch selektive hydroborierung. *Carbohydr. Res.* **1996**, *2*, 80–81.

26. Bonin, H.; Delacroix, T.; Gras, E. Dioxazaborocanes: Old adducts, new tricks. *Org. Biomol. Chem.* **2011**, *9*, 4714–4724.

27. Ni, W.; Fang, H.; Springsteen, G.; Wang, B. The Design of Boronic Acid Spectroscopic Reporter Compounds by Taking Advantage of the pKa-Lowering Effect of Diol Binding: Nitrophenol-Based Color Reporters for Diols. *J. Org. Chem.* **2004**, *69*, 1999–2007.

28. Cao, H.; McGill, T.; Heagy, M.D. Substituent Effects on Monoboronic Acid Sensors for Saccharides Based on N-Phenyl-1,8-naphthalenedicarboximides. *J. Org. Chem.* **2004**, *69*, 2959–2966.

29. Musashi, M.; Matsuo, M.; Oi, T.; Nomura, M. An anion-exchange chromatographic study on boron isotopic fractionation at 2 MPa at 293 K. *J. Chromat. A* **2006**, *1131*, 97–102.

30. Uenishi, J.; Matsui, K.; Wada, A. Trienylboronic acid, a versatile coupling tool for retinoid synthesis; stereospecific synthesis of 13-aryl substituted (11Z)-retinal. *Tetrahedron Lett.* **2003**, *44*, 3093–3096.

31. McGregor, N.; Pardin, C.; Skene, W.G. Using Quenching Kinetics and Thermodynamics of Amino-Fluorophores as Empirical Tools for Predicting Boronic Acid Sensors Suitable for Use in Physiological Conditions. *Aust. J. Chem.* **2011**, *64*, 1438–1446.

32. Hargrove, A.E.; Ellington, A.D.; Anslyn, E.V.; Sessler, J.L. Chemical Functionalization of Oligodeoxynucleotides with Multiple Boronic Acids for the Polyvalent Binding of Saccharides. *Bioconj. Chem.* **2011**, *22*, 388–396.

33. Zhong, Q.; Ngim, K.K.; Sun, M.; Li, J.; Deese, A.; Chetwyn, N.P. Strategies for the analysis of highly reactive pinacolboronate esters. *J. Chromat. A* **2012**, *1229*, 216–222.
34. Wolkenstein, K.; Gross, J.H.; Falk, H. Boron-containing organic pigments from a Jurassic red alga. *Proc. Natl. Acad. Sci. USA* **2010**, *107*, 19374–19378.
35. Claessens, C.G.; Torres, T. Subphthalocyanine enantiomers: First resolution of a C3 aromatic compound by HPLC. *Tetrahedron Lett.* **2000**, *41*, 6361–6365.
36. Nanjappan, P.; Ramallngam, K.; Nowotnik, D.P. Synthesis of the Four Stereoisomers of 1-Azabicyclo[2.2,2]oct-3-yl-α -hydroxy-α-(4-phenylboronic acid)-α-phenylacetate (QNB-Boronic acid), including a preparative HPLC method to separate diastereoisomeric mixtures with high optical purity. *Tetrahedron Asymmetry* **1992**, *3*, 1271–1282.
37. Moriyasu, M.; Endo, M.; Ichimaru, M.; Mizutani, T.; Kato, A. High Performance Liquid Chromatographic Behavior of Difluoroborane Derivatives of β-Diketones. *Anal. Sci.* **1990**, *6*, 45–48.
38. Sun, J.; Perfetti, M.T.; Santos, W.L. A Method for the Deprotection of Alkylpinacolyl Boronate Esters. *J. Org. Chem.* **2011**, *76*, 3571–3575.
39. Prichard, K.; Campkin, D.; O'Brien, N.; Kato, A.; Fleet, G.W.J.; Simone, M.I. Biological Activities of 3,4,5-Trihydroxypiperidines and their O- and N-Alkylated Derivatives. *Chem. Biol. Drug Des.* **2018**, *92*, 1171–1197.
40. Glenister, A.; Simone, M.; Hambley, T.W. A Warburg effect targeting vector designed to increase the uptake of compounds by cancer cells demonstrates glucose and hypoxia dependent uptake. *PLoS ONE* **2019**, *14*, e0217712.
